# 3D CNN-CVAE and LSTM-guided de novo design of rotigotine derivatives targeting dopamine D1 and D3 receptors in restless legs syndrome

**DOI:** 10.1371/journal.pone.0356614

**Published:** 2026-10-09

**Authors:** Sinan Eliaçık, Nouman Ali

**Affiliations:** 1 Department of Neurology, Faculty of Medicine, Hitit University, Çorum, Turkey; 2 Department of Molecular Biosciences, Faculty of Physical and Biological Sciences, Rashid Latif Khan University, Lahore, Punjab, Pakistan; UP Diliman: University of the Philippines Diliman, PHILIPPINES

## Abstract

Restless legs syndrome (RLS) is a chronic neurological disorder associated with altered dopaminergic signaling, while long-term dopamine agonist therapy may be limited by augmentation and reduced clinical benefit. This study applied a three-dimensional convolutional neural network conditional variational autoencoder integrated with long short-term memory molecular generation to design rotigotine-derived candidates targeting dopamine D1 and D3 receptors. Fourteen unique derivatives were generated and screened through pocket prediction, molecular docking, interaction profiling, pharmacophore analysis, density functional theory, ADMET prediction, 500 ns molecular dynamics simulations performed in explicit water without a lipid bilayer, and MM/GBSA calculations. Docking protocol validation using DockRMSD yielded RMSD values of 1.009 Å for Mevidalen in the D1 receptor and 1.359 Å for PD-128907 in the D3 receptor, supporting acceptable reproduction of both experimental binding poses. In docking-based ranking, AI Derivative 1 was placed above rotigotine for D1 (−5.744 vs −5.629 kcal/mol) and D3 (−7.934 vs −7.288 kcal/mol) and formed additional hydrogen-bond and hydrophobic contacts; the D1 difference of approximately 0.1 kcal/mol lies within the intrinsic uncertainty of docking scoring functions and is not interpreted as evidence of improved affinity. Docking scores are used throughout as ranking and prioritization metrics rather than quantitative binding free energies. It also exhibited a smaller HOMO-LUMO gap, a higher predicted LD_50_, and a shift from toxicity class 3 to class 4. Molecular dynamics analyses, including RMSD, RMSF, radius of gyration, solvent-accessible surface area, hydrogen bonding, DCCM, PCA, and free-energy landscapes, are reported as descriptive observations from single non-membrane trajectories and are not used to claim physiological relevance or superior binding stability. MM/GBSA estimates were favorable under the applied computational conditions. AI Derivative 1 therefore represents a computationally prioritized lead requiring experimental validation.

## Introduction

Restless Legs Syndrome (RLS) is a prevalent neurological disorder that poses a significant clinical challenge due to its chronic nature, unclear pathophysiology, and impact on quality of life. It affects up to 10% of the general population and is characterized by an uncontrollable urge to move the legs, often accompanied by uncomfortable sensations that worsen during rest and at night, leading to severe sleep disturbances and associated comorbidities [1]. Despite its high prevalence, the underlying mechanisms of RLS remain incompletely understood, although substantial evidence implicates dysfunction in dopaminergic neurotransmission and associated neural circuits as a central factor in disease progression [2]. Clinically, RLS presents with hallmark symptoms including periodic limb movements during sleep, circadian worsening of discomfort, and temporary relief upon movement, all of which significantly impair sleep quality and daily functioning [3]. Current therapeutic strategies primarily rely on dopaminergic agents, such as pramipexole, ropinirole, and rotigotine, which act as dopamine receptor agonists to alleviate symptoms. While these treatments are initially effective, long-term use is often associated with complications such as augmentation, where symptoms progressively worsen over time, highlighting the need for improved therapeutic strategies [4].

At the molecular level, dopamine receptors, particularly the D1 and D3 subtypes, play a critical role in the pathophysiology and treatment of RLS. The D3 receptor, predominantly expressed in the limbic and spinal sensory-processing regions, is closely associated with inhibitory modulation of sensory input and is a primary target of current dopaminergic therapies [3]. In contrast, the D1 receptor is widely distributed in motor regions and is involved in excitatory signaling pathways that regulate movement. Importantly, D1 and D3 receptors can form functional heteromeric complexes, where their interaction modulates dopaminergic signaling and motor control [5]. Dysregulation of this balance, particularly during prolonged dopaminergic treatment, has been suggested to contribute to the development of augmentation and reduced therapeutic efficacy, making the dual targeting of D1 and D3 receptors a rational and emerging strategy for improved RLS management [5].

Rotigotine, a non-ergoline dopamine agonist, serves as an important therapeutic agent and a suitable reference ligand in this context. It exhibits agonistic activity across multiple dopamine receptor subtypes, including D1, D2, and D3, with relatively higher affinity for the D3 receptor while maintaining functional activity at D1 receptors [6]. Clinically, rotigotine is administered via a transdermal delivery system, providing continuous dopaminergic stimulation and demonstrating efficacy in the management of RLS symptoms [7]. However, similar to other dopaminergic therapies, its long-term use may still be associated with limitations such as tolerance and augmentation, necessitating the exploration of optimized derivatives with improved pharmacological profiles. Pharmacological studies further support the relevance of rotigotine in RLS, demonstrating that dopamine agonists targeting D3 receptors are effective in reducing sensorimotor symptoms, although their prolonged use may alter receptor dynamics and lead to compensatory changes involving D1 receptor activity [8]. This highlights the importance of designing compounds capable of modulating both receptor subtypes in a balanced manner to achieve sustained therapeutic outcomes.

In recent years, structure-based drug design (SBDD) and computer-aided drug design (CADD) approaches have emerged as powerful strategies for identifying and optimizing novel therapeutic candidates [9]. These approaches enable the rational design of ligands based on the three-dimensional structure of target proteins, allowing for detailed analysis of binding interactions, pharmacophore features, and dynamic behavior of protein–ligand complexes [10]. Computational techniques such as molecular docking, molecular dynamics simulation, and free energy calculations have significantly enhanced the efficiency of drug discovery by reducing time, cost, and experimental burden while providing mechanistic insights at the molecular level [11].

Artificial intelligence (AI) has recently emerged as a transformative approach in drug discovery and optimization, enabling the efficient exploration of chemical space and rational design of novel therapeutics [12,13]. AI-driven models, particularly deep learning architectures such as variational autoencoders and recurrent neural networks, have demonstrated the ability to generate chemically valid and structurally diverse compounds while preserving key pharmacophoric features of known drugs [14]. These approaches allow for the optimization of molecular properties, including binding affinity, selectivity, and pharmacokinetic behavior, in a data-driven manner that significantly reduces the time and cost associated with traditional drug development pipelines [15,16]. Moreover, AI-based de novo design frameworks can capture complex structure–activity relationships and propose novel derivatives that may not be readily accessible through conventional medicinal chemistry strategies [17]. Recent studies have highlighted the successful application of AI in generating lead compounds with improved biological activity and drug-like properties, supporting its integration into structure-based drug design workflows [18,19].

Although several computational studies have explored dopamine receptor agonists and AI-assisted drug discovery independently, limited studies have integrated AI-driven de novo optimization specifically for rotigotine derivatives targeting both D1 and D3 dopamine receptors in the context of restless legs syndrome. Furthermore, the combined application of AI-based molecular generation, dual-receptor docking, pharmacophore characterization, DFT analysis, ADMET profiling, long-timescale molecular dynamics simulation, and MM/GBSA binding free energy analysis for rotigotine derivative optimization has not been comprehensively investigated. The study is framed throughout as a computational prioritization exercise: D1 and D3 are treated as separate targets in independent calculations, and functional agonism, receptor selectivity, D1-D3 heteromer engagement, and therapeutic benefit in restless legs syndrome are stated as biological hypotheses motivating the design rather than as outcomes assessed here. Therefore, the present study aims to design and evaluate AI-optimized rotigotine derivatives targeting dopamine D1 and D3 receptors using an integrated computational approach.

## Methodology

### Retrieval of D_1_ and D_3_ Receptors 3D structure and validation

The three-dimensional structures of the human dopamine D1 and D3 receptors were retrieved from the RCSB Protein Data Bank (https://www.rcsb.org/) [10,20]. The cryo-electron microscopy structures corresponding to PDB IDs 7CKZ (D1 receptor) and 7CMV (D3 receptor) were selected on the basis of their reported resolutions (3.10 Å and 2.70 Å, respectively) and their relevance to ligand-bound receptor conformations. Both entries are single-particle cryo-electron microscopy structures and are referred to as such throughout the manuscript; they are not X-ray crystal structures. The structures were downloaded in PDB format and visually inspected to ensure structural completeness and proper chain organization prior to further analysis.

To evaluate the stereochemical quality and structural reliability of the retrieved protein models, validation was performed using the SAVES v6.1 server (https://saves.mbi.ucla.edu/) [21,22]. This platform integrates multiple validation tools, including PROCHECK for Ramachandran plot analysis for overall structural quality assessment [23]. The Ramachandran plot was used to assess backbone dihedral angles and identify residues in favored, allowed, and disallowed regions, ensuring that the protein conformations were suitable for downstream computational studies. This validation step is essential to confirm that the selected receptor structures possess acceptable stereochemical quality, as structural inaccuracies can significantly affect molecular docking accuracy and subsequent interaction analyses.

### Retrieval of rotigotine and 3D CNN-CVAE/LSTM-based de novo generation of derivatives

The three-dimensional conformer of rotigotine was retrieved from the PubChem database (https://pubchem.ncbi.nlm.nih.gov/) using compound identifier CID 59227 and downloaded in SDF format [24,25]. Its canonical SMILES representation was also obtained for computational molecular generation and subsequent cheminformatics analyses. Rotigotine was selected as the reference scaffold because of its established clinical use as a dopamine receptor agonist in restless legs syndrome.

De novo molecular generation was performed using the WADDAICA platform (https://heisenberg.ucam.edu:5000/), which employs a deep generative framework integrating a three-dimensional convolutional neural network conditional variational autoencoder (3D CNN-CVAE) with a long short-term memory (LSTM)-based molecular captioning model [26–28]. The 3D CNN component extracts spatial and molecular features from the input scaffold, whereas the conditional variational autoencoder represents these features within a continuous latent space. Controlled sampling of this latent representation enables the generation of structurally modified molecules while retaining relevant features of the parent compound. The LSTM component supports conversion of the generated molecular representations into chemically interpretable sequences. The WADDAICA platform is provided as a publicly hosted web service and is not distributed as a numbered software release; accordingly, no version identifier is available for citation, and all generation runs reported here were performed using the publicly accessible implementation at the address above. All generation settings were retained at the platform defaults, and the web interface does not expose user-adjustable network hyperparameters, latent-space sampling temperature, or random-seed control. Because latent-space sampling is stochastic and the seed cannot be specified by the user, the generated output is reproducible at the level of the input scaffold and the platform defaults rather than as an exactly reproducible list of structures. For this reason, the complete set of generated outputs and the retained final structures have been deposited in machine-readable format to permit direct reuse and independent re-evaluation (see Data Availability).

The SMILES representation of rotigotine was submitted as the reference input, and 30 candidate structures were initially generated. Duplicate and chemically inconsistent outputs were removed, resulting in 14 unique rotigotine-derived molecules. Filtering was performed in three sequential steps. First, canonical SMILES were generated for all 30 outputs and exact duplicates were removed. Second, structures that failed automated sanitization and valence checking, or that contained undefined or chemically unstable valence states, were rejected as chemically inconsistent. Third, outputs that no longer retained the minimal rotigotine-like framework, defined as an aromatic ring connected through an aliphatic linker to a basic secondary or tertiary amine, were excluded because such structures fall outside the intended scaffold-constrained design space. Fourteen structures satisfied all three criteria and were retained for subsequent analysis. The retained derivatives were inspected for structural validity and converted into MOL2, PDB, and SDF formats for molecular docking, interaction analysis, pharmacophore characterization, ADMET prediction, and molecular dynamics simulations. This procedure enabled controlled exploration of chemical space around the rotigotine scaffold but did not establish the synthetic accessibility or biological activity of the generated compounds. It should be stated explicitly that AI Derivative 1 was selected for the subsequent analyses on the basis of its docking rank alone. The remaining thirteen derivatives were not subjected to density functional theory, detailed ADMET profiling, molecular dynamics simulation, or MM/GBSA analysis. The results reported for AI Derivative 1 therefore characterize the docking-selected compound and do not demonstrate that it possesses the best overall multi-parameter profile among the fourteen generated structures. A consistent multi-parameter screen applied to the full set was outside the scope of this study, and previous work has shown that receptor-based [56] and ADMET analyses can substantially reorder a ranking obtained from an initial computational model [29]. It should be emphasized that chemical validity and scaffold retention were the only criteria applied at the generation stage. No synthetic-accessibility scoring, retrosynthetic analysis, or medicinal-chemistry liability filter was used to select the 14 retained derivatives. The retained structures are therefore reported as computationally proposed molecules whose practical synthesizability has not been assessed, and this is stated explicitly as a limitation of the present design workflow.

### Prediction of D_1_ and D_3_ receptors active sites

The active binding sites of the D_1_ and D_3_ dopamine receptors were predicted using the PrankWeb server (https://prankweb.cz/), a machine learning-based tool designed for ligand binding site identification [29,30]. The prepared receptor structures in PDB format were uploaded to the server, and pocket prediction analysis was performed using default parameters. Among the predicted pockets, Pocket 1 was selected for subsequent molecular docking studies based on its highest score and probability, indicating a better likelihood of being a biologically relevant binding site. For the D1 receptor (7CKZ), Pocket 1 is lined by residues 59, 60, 62, 63, 117, 120, 121, 124, 125, 217, 221, 224, 225, 270, 273, 277, 330, 331, 334 and 337 of chain R. These residues lie in the intracellular halves of TM1, TM3, TM5, TM6 and TM7 and in the region immediately adjacent to intracellular loop 2, and therefore correspond to the inner-surface allosteric region of the D1 receptor in which the positive allosteric modulator LY3154207 (mevidalen) is resolved in 7CKZ, rather than to the extracellular orthosteric pocket occupied by dopamine. This assignment is supported empirically by the redocking validation described below: mevidalen re-docked into this grid reproduced its experimentally resolved pose with a symmetry-corrected heavy-atom RMSD of 1.009 Å, which would not be attainable if the grid did not encompass the experimental allosteric binding site. The ligand used for docking validation is therefore native to the same site that was used for compound screening, as required for a meaningful validation. Accordingly, all D1 results reported in this manuscript describe engagement of this allosteric region only, and are not presented as orthosteric binding, receptor activation, or agonism at the D1 receptor. For the D3 receptor (7CMV), Pocket 1 corresponds to the orthosteric binding pocket. This is supported by the interaction analysis, in which the docked ligands contact ASP110 and SER196, the canonical orthosteric anchor residues of the D3 receptor, and by the redocking of the orthosteric native ligand PD-128907, which reproduced its experimental pose with an RMSD of 1.359 Å.

The server also provided detailed information, including pocket coordinates, residue composition, and structural features, which were used to define the docking grid parameters. Although co-resolved ligands were present in the receptor structures, active site prediction was performed to independently validate the binding region and ensure unbiased docking analysis. The identification of accurate binding pockets is a critical step in structure-based drug design, as it ensures that molecular docking is performed within biologically relevant regions, thereby improving the reliability and interpretability of ligand–receptor interaction results.

### Preparation of receptor protein

Prior to molecular docking and molecular dynamics simulations, the retrieved D_1_ and D_3_ dopamine receptor structures were prepared to obtain chemically accurate and energetically stable models. Initial preprocessing was performed using BIOVIA Discovery Studio Visualizer to remove all non-essential heteroatoms, including experimentally resolved water molecules and co-resolved ligands, in order to eliminate potential steric hindrance and expose the predicted active binding sites for ligand interaction [31]. Both deposited entries correspond to active-state signalling complexes rather than isolated receptors. Accordingly, only the receptor polypeptide was retained for all subsequent calculations, corresponding to chain R in both 7CKZ and 7CMV, while the heterotrimeric G protein subunits, the stabilising nanobody and single-chain antibody fragments, and all remaining non-receptor polypeptides were deleted. Bound ions, lipids, detergent molecules, and other additives present in the deposited models were also removed. All experimentally resolved water molecules were deleted for three reasons: at the reported resolutions of 3.10 Å and 2.70 Å comparatively few ordered water molecules are modelled, and none were located within the Pocket 1 volume subsequently used to define the docking grid; the docking calculations treated the receptor as rigid and without explicit structural water, so retaining a partial and unequally resolved water network would have introduced an inconsistency between the D1 and D3 systems; and explicit hydration was reintroduced for both complexes during the molecular dynamics simulations. We acknowledge that this treatment cannot reproduce water-bridged ligand-receptor contacts that may be present in the experimental models, and this is noted in the Limitations section.

Further structural refinement was conducted using PDBFixer to identify and correct missing atoms, incomplete side chains, and other structural inconsistencies commonly present in experimentally resolved protein structures [32]. In practical terms, missing heavy atoms and incomplete side chains within the retained receptor chain were rebuilt, and hydrogen atoms were added and protonation states were assigned at pH 7.4 within the SAMSON Connect environment. Under these conditions aspartate and glutamate residues were treated as deprotonated, lysine and arginine residues as protonated, and histidine residues as neutral, and no non-standard protonation state was imposed on any binding-site residue. Short disordered segments and unresolved terminal residues lying outside the transmembrane bundle were not rebuilt de novo, since ab initio reconstruction of such regions would introduce structural uncertainty without affecting the binding site under investigation. No mutations, fusion partners, or engineered thermostabilising substitutions present in the deposited constructs were reverted, and the receptor sequences were used as deposited. Following correction, restrained energy minimization was performed using Swiss-PDB Viewer to relieve steric clashes and optimize bond geometries while maintaining the overall structural integrity of the receptors [33]. Protein preparation is a crucial step in computational studies, as incomplete or improperly optimized structures can lead to inaccurate docking poses and unreliable interaction profiles. Generating refined receptor models ensures greater reliability and reproducibility in all subsequent structure-based analyses.

### Molecular docking and interaction analysis of D_1_ and D_3_ receptors with CNN-generated derivatives

Molecular docking studies were performed using the AutoDock Vina docking extension implemented within the SAMSON Connect platform to evaluate the binding affinity of Rotigotine and its AI-generated derivatives toward the D_1_ and D_3_ dopamine receptors [34]. Prior to docking, polar hydrogen atoms were added to both receptor and ligand structures, and ligand geometries were energy-minimized to obtain stable conformations suitable for docking analysis. Ligand structures were prepared within the same SAMSON Connect environment and at the same pH of 7.4 used for receptor preparation. Under these conditions the basic aliphatic amine present in rotigotine and in the AI-generated derivatives is protonated, corresponding to a net formal charge of +1 for each ligand. The stereochemistry of rotigotine was retained as retrieved from PubChem, and the configuration generated for each derivative was preserved without enantiomeric conversion or racemization. All rotatable bonds were assigned automatically and the ligands were treated as fully flexible during docking, whereas the receptor was held rigid. The same ligand geometries and ionization states were subsequently parameterized with the CHARMM General Force Field for the molecular dynamics simulations, so that the docking and simulation stages describe the same chemical species.

The docking grid was defined based on the coordinates of the predicted active site (Pocket 1) obtained from PrankWeb. For the D1 receptor, the grid center was set at X = 7.7 Å, Y = −2.0 Å, and Z = 4.0 Å, while for the D3 receptor, the grid center was defined at X = 93.8 Å, Y = 122.2 Å, and Z = 117.8 Å. In both cases, the grid box dimensions were set to 20 Å × 20 Å × 20 Å along the X, Y, and Z axes to adequately encompass the binding pocket. A grid spacing of 1.0 Å and an exhaustiveness value of 8 were used to balance computational efficiency with sufficient conformational sampling. Docking was carried out using the default Vina search settings exposed by this interface, namely a maximum of nine output poses per ligand and an energy range of 3 kcal/mol relative to the best-scoring pose. The random seed is generated internally by the interface and is neither user-specifiable nor reported, so the stochastic search cannot be reproduced exactly. A single docking run was performed for each ligand and the stability of the resulting rank order was therefore not assessed by repeated runs. For this reason, and as stated above, the docking scores are used only as a coarse prioritization metric, and the small score difference obtained for the D1 receptor is not treated as a reliable rank separation.

The docking protocol was evaluated through re-docking of the native co-resolved ligand into its experimentally defined receptor-binding pocket using the same grid coordinates and docking parameters applied during compound screening. The experimentally resolved and re-docked ligand conformations were exported in MOL2 format and compared using DockRMSD (https://zhanggroup.org/DockRMSD/) [35]. This algorithm identifies graph-isomorphic atom mappings and accounts for chemically equivalent and symmetric atoms when calculating the minimum heavy-atom root mean square deviation between two ligand poses. The resulting RMSD was used as a quantitative measure of the ability of the docking procedure to reproduce the experimentally observed binding orientation. An RMSD below 2.0 Å was considered indicative of acceptable pose reproduction. It should be emphasized that this validation demonstrates only that the docking protocol is able to reproduce an experimentally observed binding pose within the selected pocket. It does not validate the predicted binding affinities, does not establish that the score difference between two ligands is quantitatively meaningful, and provides no evidence of agonist activity at either receptor. PyMOL was used only for visual inspection and graphical representation of the superimposed experimental and re-docked ligand poses and was not used to calculate the reported RMSD.

For each ligand, multiple binding poses were generated, and the conformation with the lowest binding energy was selected for further analysis. Post-docking interaction studies were conducted using BIOVIA Discovery Studio Visualizer to generate two-dimensional interaction diagrams, enabling detailed visualization of hydrogen bonding, hydrophobic interactions, and other non-covalent interactions [31]. Additionally, the Protein–Ligand Interaction Profiler (PLIP) (https://plip-tool.biotec.tu-dresden.de/plip-web/plip/index) was used to systematically identify and categorize 3D interactions, while PyMOL was employed to visualize docking poses and analyze binding orientations within the receptor active site [36–38]. Molecular docking provides an initial estimation of binding affinity and interaction patterns between ligands and target receptors, serving as a fundamental step in identifying potential lead compounds for further computational and experimental evaluation. Throughout this study, docking scores are used exclusively as ranking and prioritization metrics for pose selection and lead triage. They are empirical scoring-function outputs and are not interpreted as quantitative binding free energies, and score differences of the order of a few tenths of a kilocalorie per mole are treated as lying within the intrinsic uncertainty of the scoring function.

### Pharmacophore characterization and Density functional theory (DFT) of lead drug candidate

Pharmacophore characterization of the lead compound and reference molecule was performed using the Pharmit server (https://pharmit.csb.pitt.edu/), an interactive platform for identifying key pharmacophoric features responsible for molecular recognition [39,40]. The optimized ligand structures were uploaded to the server, and essential features such as hydrogen bond donors, hydrogen bond acceptors, aromatic rings, and hydrophobic regions were identified and mapped. This analysis enabled a descriptive comparison of ligand feature composition and geometry between the parent compound and the AI-generated derivative. Because pharmacophore features are computed from the ligand alone, they are reported as molecular descriptors and are not used to infer receptor affinity or selectivity.

For quantum chemical evaluation, Density Functional Theory (DFT) calculations were conducted using Gaussian 09W software [9,41]. The geometries of both rotigotine and the selected lead compound were optimized at the B3LYP/6-31G(d,p) level of theory. Both molecules were treated in the gas phase, with no implicit or explicit solvent model applied, and each was computed as the neutral closed-shell species with a net molecular charge of 0 and a spin multiplicity of 1; the protonated forms used for docking and simulation were therefore not the species used for the electronic-structure calculations. Geometry optimization used the default Gaussian convergence criteria for maximum force, root-mean-square force, maximum displacement, and root-mean-square displacement. Each optimized geometry was confirmed as a true local minimum by a subsequent harmonic frequency calculation at the same level of theory, which returned no imaginary frequencies. Following optimization, frontier molecular orbitals, including the highest occupied molecular orbital (HOMO) and lowest unoccupied molecular orbital (LUMO), were calculated to assess the electronic properties and reactivity of the molecule. The HOMO–LUMO energy gap was determined as the difference between the LUMO and HOMO energies and used as an indicator of chemical stability and charge transfer potential.

Additionally, molecular electrostatic potential (MEP) mapping was performed to visualize the distribution of electron density across the molecule and to identify regions susceptible to electrophilic and nucleophilic interactions. The pharmacophore and DFT calculations are reported as descriptive characterizations of the isolated ligands. Because both are computed in the absence of the receptor, neither is used in this manuscript to infer binding affinity, selectivity, or biological activity.

### Comparative insilco AMDET analysis

The pharmacokinetic and toxicity profiles of Rotigotine and the selected AI-generated derivative were evaluated using *in silico* prediction tools. Absorption, distribution, metabolism, and excretion (ADME) properties were analyzed using the SwissADME web server (https://www.swissadme.ch/) [42,43]. The SMILES representations of the compounds were used as input, and key parameters including physicochemical properties, lipophilicity, water solubility, gastrointestinal absorption, blood–brain barrier permeability, and drug-likeness rules were assessed using default settings.

Toxicity profiling was carried out using the ProTox 3.0 server (https://tox.charite.de/protox3/), an AI- and QSAR-based platform for predicting various toxicity endpoints [18,44]. The compounds were evaluated for acute oral toxicity (LD_50_), toxicity class, organ toxicity, and multiple biological endpoints including hepatotoxicity, neurotoxicity, mutagenicity, and carcinogenicity. Additionally, interactions with nuclear receptor signaling pathways and stress response pathways were analyzed to further understand the safety profile of the compounds.

The use of *in silico* ADMET and toxicity prediction tools provides an early assessment of pharmacokinetic behavior and potential safety risks, which is essential for prioritizing lead compounds and reducing the likelihood of failure in later stages of drug development.

### Molecular dynamics simulation of lead drug candidate

Molecular dynamics (MD) simulations were performed to investigate the conformational stability and dynamic behavior of the D1 and D3 dopamine receptor complexes with the selected lead compound and reference ligand in an explicitly solvated aqueous environment. All simulations were carried out using the GROMACS 2024.3 simulation package [45]. The CHARMM36m force field was employed for receptor parameterization [46]. Ligand topologies and parameters for rotigotine and AI Derivative 1 were generated with the CHARMM General Force Field (CGenFF) using the CHARMM-GUI Ligand Reader and Modeler, which assigns atom types, bonded terms, and Lennard-Jones parameters by analogy to existing CGenFF entries and derives partial atomic charges using the CGenFF charge-assignment scheme. The assigned atom types, net charges, bonded parameters, and molecular connectivity were inspected before system assembly, and the CGenFF penalty scores were checked to confirm that no parameter required manual reoptimization. Both ligands were carried into the simulations in the same protonation and stereochemical state used for the docking calculations, so that the receptor-ligand starting geometries were internally consistent across all four systems.

Each protein–ligand complex was placed in a cubic simulation box with a minimum distance of 10 Å between the protein surface and the box edges to avoid artificial interactions with periodic images. The systems were solvated using the CHARMM-modified TIP3P explicit water model, consistent with the CHARMM force-field framework [47]. Sodium (Na⁺) and chloride (Cl^-^) ions were added to neutralize the net system charge and to reach a physiological ionic concentration of 0.15 M. It should be stated explicitly that the receptors were simulated as solvated protein constructs and that no explicit lipid bilayer was included in any of the simulation systems. D1 and D3 are class A G protein-coupled receptors whose transmembrane helical bundles are natively stabilized by a lipid environment, and a bulk-water box does not reproduce that stabilization. This setup was adopted solely to obtain a uniform and internally consistent comparison of the two ligands within the same receptor construct under identical conditions, and not as a physiologically faithful model of receptor behavior. Because the transmembrane bundle is not laterally supported in bulk water, solvent-driven relaxation and partial expansion of the helical bundle are expected, and any such structural drift reflects the simulation setup rather than a ligand-dependent effect. Accordingly, the molecular dynamics analyses reported in this manuscript are treated throughout as descriptive, exploratory observations of the individual trajectories. They are not used to support claims of physiological relevance, receptor activation, comparative binding stability, or mechanistic interpretation, and this restriction is stated in detail in the Limitations section.

Energy minimization of each system was performed using the steepest descent algorithm for 5,000 steps to remove unfavorable steric clashes and achieve a stable initial conformation. Following minimization, equilibration was conducted in two sequential phases. First, the system was equilibrated under constant number of particles, volume, and temperature (NVT ensemble) to stabilize the temperature at 310 K. Temperature coupling was maintained using the Berendsen thermostat [45]. Subsequently, equilibration was continued under constant number of particles, pressure, and temperature (NPT ensemble) to stabilize system density at 1 atm pressure using the Parrinello–Rahman barostat [46]. The NVT and NPT equilibration phases were each carried out for 100 ps using a 2 fs time step. Position restraints applied to the receptor and ligand heavy atoms during equilibration were released before the production run. Production simulations were conducted in the NPT ensemble at 310 K and 1 atm, with temperature maintained by the Berendsen thermostat and pressure by the Parrinello-Rahman barostat using isotropic coupling, and with the receptor-ligand complex and the solvent-plus-ion group coupled separately.

During the simulation, all covalent bonds involving hydrogen atoms were constrained using the LINCS algorithm, allowing the use of a 2 fs integration time step [50]. Long-range electrostatic interactions were calculated using the particle mesh Ewald (PME) method with a real-space cutoff of 1.2 nm, while short-range nonbonded interactions were handled using the Verlet cutoff scheme [51]. Lennard-Jones interactions were evaluated using a force-switching function between 1.0 and 1.2 nm, in accordance with the recommended CHARMM36 simulation settings. Periodic boundary conditions were applied in all three spatial dimensions to simulate an infinite system.

After successful equilibration, production MD simulations were performed for 500 ns for each protein–ligand complex. Atomic coordinates were written every 100 ps, and all reported analyses were performed on this saved trajectory interval. Trajectories were analyzed using standard GROMACS tools to evaluate structural stability, flexibility, and compactness. Root mean square deviation (RMSD) was calculated to assess the stability of the protein backbone over time, while root mean square fluctuation (RMSF) was used to evaluate residue-level flexibility. The radius of gyration (Rg) was determined to examine the compactness of the protein structure, and solvent-accessible surface area (SASA) was calculated to assess the exposure of the protein to the solvent environment. Hydrogen bond analysis was performed to evaluate the stability and persistence of intermolecular interactions throughout the simulation.

Dynamic cross-correlation matrix (DCCM) analysis was conducted to investigate correlated and anti-correlated motions between residues, providing insight into collective protein dynamics. Principal component analysis (PCA) was performed to capture dominant motions and conformational transitions during the simulation. Free energy landscape (FEL) analysis was generated based on principal components to identify stable conformational states of the complexes. All trajectory analyses were carried out with the standard GROMACS analysis tools on trajectories that had first been corrected for periodic boundary effects, using whole-molecule reconstruction and receptor centering followed by least-squares rotational and translational fitting of the receptor backbone to the starting structure. RMSD and radius of gyration were computed on the receptor backbone, RMSF on Cα atoms on a per-residue basis, solvent-accessible surface area on the receptor-ligand complex, and hydrogen bonds between ligand and receptor using the default geometric criteria implemented in the GROMACS hydrogen-bond tool. Dynamic cross-correlation matrices were computed from Cα coordinates over the fitted trajectory. Principal component analysis was performed by constructing and diagonalizing the covariance matrix of the Cα coordinates after the same least-squares fit, and the trajectory was projected onto the first two eigenvectors. Free energy landscapes were constructed from the probability distribution P of these first two principal components using the relation G = -kBT ln(P/Pmax), with energies expressed in kJ/mol and normalized so that the most populated state is assigned a value of zero. The eigenvalue spectra, the fraction of total variance captured by the retained components, and the numerical data underlying all reported trajectory plots are provided in the public repository listed under Data Availability. A comparable methodological treatment, in which RMSD is examined together with centre-of-mass distance in order to identify changes in ligand binding mode, has been described previously [47].

To further quantify binding affinity, Molecular Mechanics Generalized Born Surface Area (MM/GBSA) calculations were performed using representative frames extracted from the simulation trajectories. The binding free energy was calculated as the difference between the free energy of the protein–ligand complex and the sum of the free energies of the isolated protein and ligand. Additionally, per-residue energy decomposition analysis was carried out to identify key amino acid residues contributing significantly to ligand binding and stabilization. Binding free-energy calculations were performed with gmx_MMPBSA (version 1.6.5) using a single-trajectory protocol. A total of 500 frames were extracted from each production trajectory at a uniform spacing of 1 ns across the full 500 ns simulation, and the identical interval and frame count were used for all four receptor-ligand systems so that the estimates remain directly comparable. The Generalized Born OBC1 implicit solvent model (igb = 2) was applied with an internal dielectric constant of 1.0, an external dielectric constant of 80.0, and an ionic strength of 0.15 M. The non-polar solvation contribution was estimated from the solvent-accessible surface area using the default LCPO surface-area model implemented in gmx_MMPBSA. Per-residue free-energy decomposition was carried out on the same set of frames. The free energy of binding was calculated by subtracting the sum of the ligand and receptor free energies from that of the complex (Equation 1). The per-residue contribution to the binding free energy was assessed via Equation 2 to identify key interacting residues. Entropic contributions were not computed and are not included in any reported total. The MM/GBSA values reported in this manuscript are therefore enthalpic effective binding energies rather than absolute binding free energies. The configurational free-energy landscapes derived from principal component analysis are presented separately as descriptive characterizations of the sampled conformational space and do not provide, and are not used as, a solute-entropy term for the MM/GBSA calculations. Molecular dynamics simulations provide a time-resolved understanding of protein–ligand interactions beyond static docking, allowing the evaluation of stability, flexibility, and binding energetics under dynamic conditions, thereby strengthening the reliability of computational predictions.


$$\begin{array}{c} \Delta \text{Gbind }=\text{ Gcomplex }-\;(\text{Gprotein }+\text{ Gligand}) \end{array}$$ (1)

$$\begin{array}{c} \Delta \text{Ginhibitor}-\text{residue }=\;\Delta \text{GvdW }+\;\Delta \text{Gele }+\;\Delta \text{Gele},\text{ sol }+\;\Delta \text{Gnon}-\text{pol},\text{ sol} \end{array}$$ (2)

### Use of generative artificial intelligence in manuscript preparation

Generative artificial intelligence was used during manuscript preparation for language editing only. ChatGPT (GPT-5.6 Thinking; https://chatgpt.com/) was applied to refine wording, correct grammar, adjust sentence structure, and improve the overall readability of the text. No generative artificial intelligence tool was used to generate, fabricate, modify, or analyze any experimental or computational data, figures, tables, scientific interpretations, or conclusions, and none of the computational procedures described above depended on such tools. All AI-assisted language edits were subsequently reviewed and verified by the authors, who take full responsibility for the accuracy, integrity, and content of the submitted manuscript.

## Results

### Retrieval of D1 and D3 Receptors 3D structure and validation

The three-dimensional structures of the D1 and D3 dopamine receptors were successfully obtained in high-resolution cryo-electron microscopy formats, providing structurally reliable templates for subsequent computational analyses (Fig 1A and 1B). The D1 receptor (7CKZ) exhibited a resolution of 3.10 Å, while the D3 receptor (7CMV) demonstrated a higher resolution of 2.70 Å, indicating slightly improved structural clarity for the latter. Both receptors were confirmed as membrane proteins with experimentally resolved conformations suitable for ligand interaction studies.

**Fig 1:**
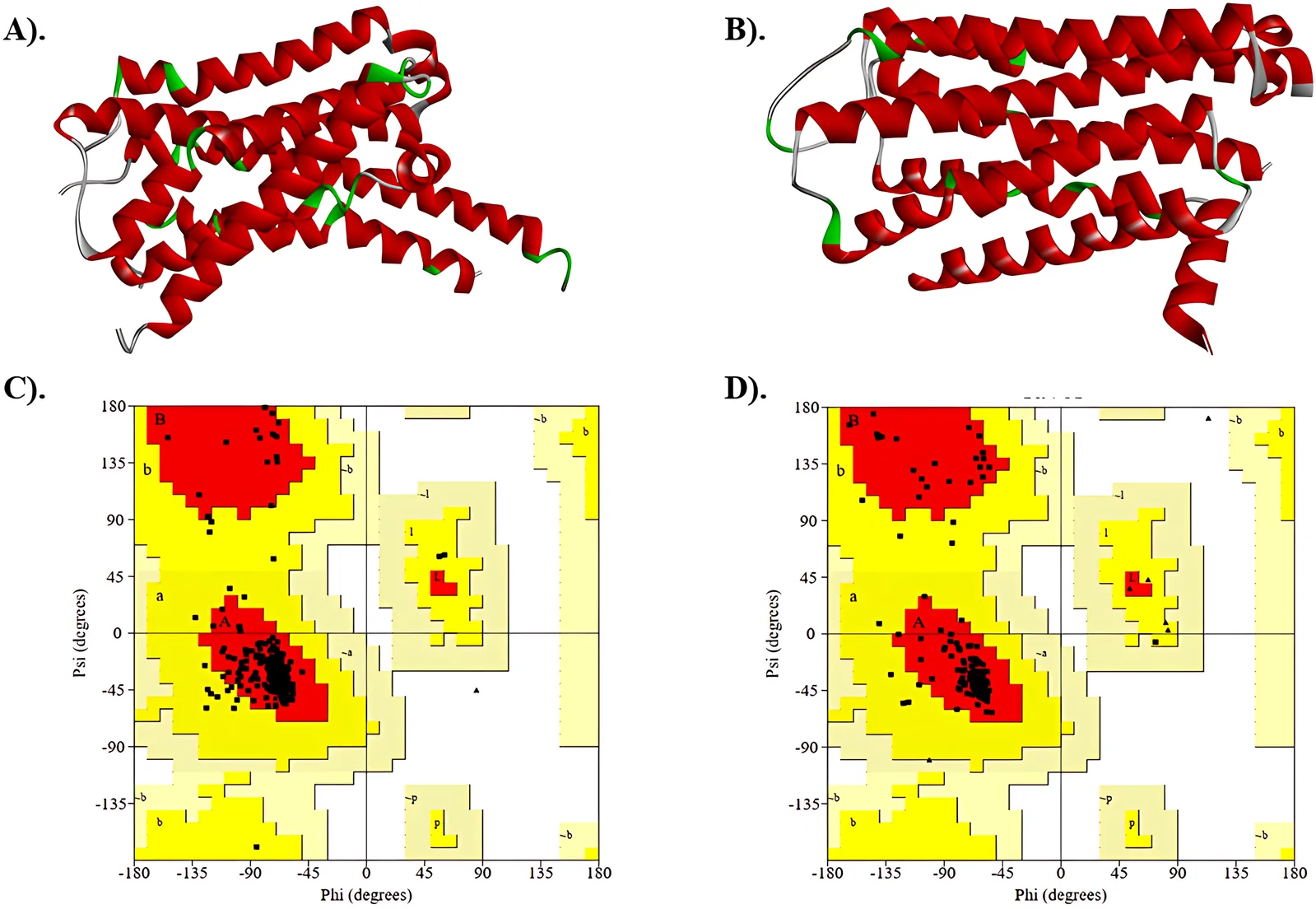
(A). Three-dimensional cryo-electron microscopy structure of the dopamine D1 receptor (PDB ID: 7CKZ) showing overall protein architecture used for computational analysis. **(B).** Three-dimensional cryo-electron microscopy structure of the dopamine D3 receptor (PDB ID: 7CMV) illustrating the receptor conformation and binding environment. **(C).** Ramachandran plot of the D1 receptor and **(D).** Ramachandran plot of the D3 receptor demonstrating acceptable stereochemical quality with most residues positioned in energetically favorable regions, supporting its suitability for subsequent docking and simulation studies.

Structural validation through Ramachandran plot analysis revealed that the D1 receptor had 92.5 percent of residues located within the most favored regions, with the remaining 7.5 percent in additionally allowed regions and no residues in disallowed regions (Fig 1C). Similarly, the D3 receptor demonstrated 95.6 percent of residues in the most favored regions and 4.4 percent in additionally allowed regions, also with no residues in disallowed regions (Fig 1D). These findings suggest that both receptor structures possess acceptable stereochemical quality suitable for subsequent computational analyses.

Both structures meet the accepted thresholds for high-quality protein models, supporting their suitability for molecular docking and simulation studies. The acceptable structural quality of these receptors supports the reliability of subsequent computational docking and simulation analyses. Any structural deviation at this stage could propagate errors into downstream analyses; therefore, the validated quality of both receptors strengthens the confidence in all following computational predictions.

### 3D CNN-CVAE/LSTM-based generation of rotigotine derivative

The AI-driven de novo design process successfully generated a diverse set of Rotigotine-derived compounds, expanding the chemical space beyond the parent scaffold while retaining key structural features relevant to dopaminergic activity. From the initially generated pool, a total of fourteen unique derivatives were obtained after removing duplicate and chemically inconsistent structures, ensuring that only valid and structurally distinct molecules were considered for further analysis (S1 Table).

The generated derivatives exhibited notable structural diversity, particularly in terms of aromatic substitutions, alkyl chain modifications, and variations in amine functional groups. Despite these modifications, the core pharmacophoric elements of Rotigotine, such as the presence of aromatic rings and amine linkages, were largely preserved across the derivatives. This balance between structural conservation and diversification may facilitate exploration of new binding interactions while retaining compatibility with known dopamine receptor binding features.

Among the generated compounds, certain derivatives, particularly AI Derivative 1, displayed structural features that may support favorable receptor interaction. These include modified hydrophobic moieties and spatial arrangements that may support favorable interactions within the receptor pocket. Such modifications may be relevant when considered alongside the predicted docking affinities and interaction profiles observed in subsequent analyses.

The generation of chemically valid and structurally diverse derivatives provides a suitable foundation for subsequent computational screening. By systematically modifying the parent scaffold, this approach increases the likelihood of identifying compounds with improved binding affinity, stability, and pharmacokinetic properties. These derivatives were therefore carried forward for detailed docking, interaction, and dynamic analyses to evaluate their predicted biological relevance.

### Prediction of D_1_ and D_3_ receptors active sites

The binding site prediction analysis identified multiple potential ligand-binding pockets within both the D1 and D3 dopamine receptor structures, with Pocket 1 consistently emerging as the most significant site based on its highest score and probability (Fig 2A and 2B). This pocket was characterized by an optimal spatial arrangement of residues capable of supporting ligand accommodation and stabilizing intermolecular interactions.

**Fig 2:**
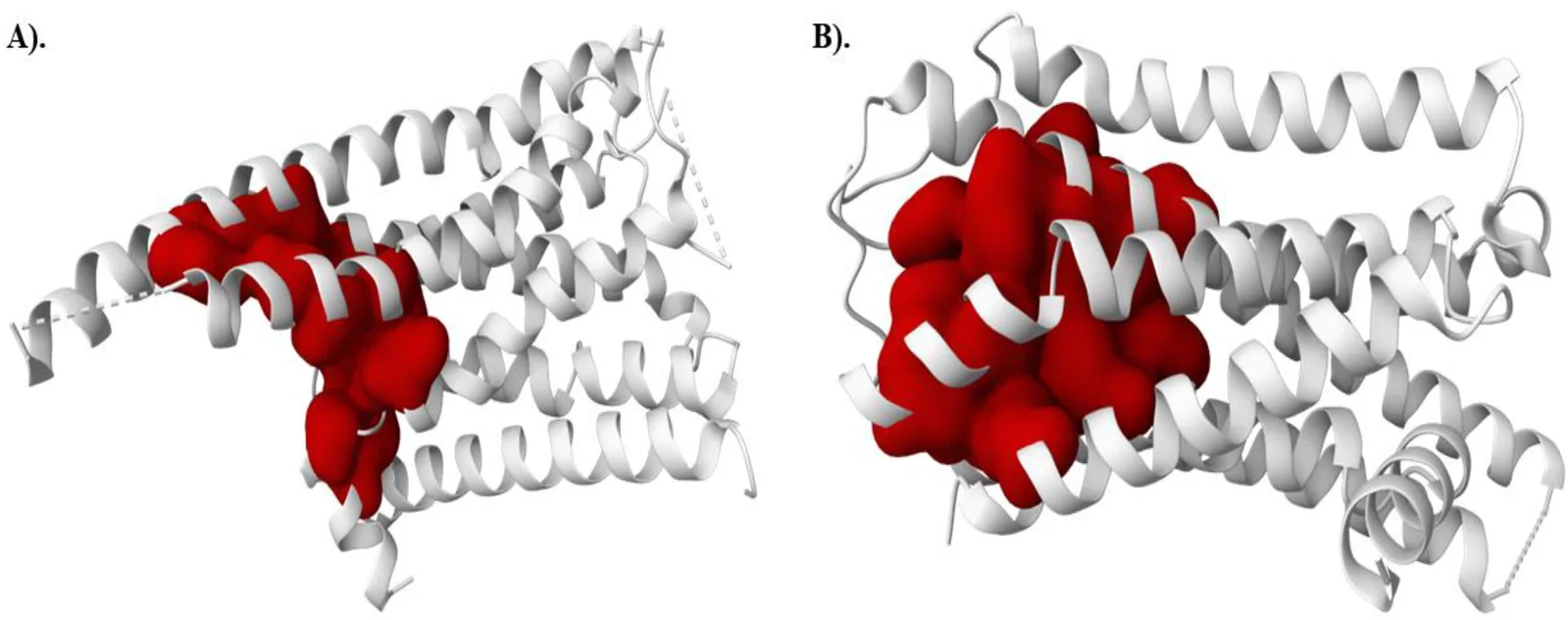
Active site identification of dopamine receptors. **(A).** Predicted active site of the dopamine D1 receptor (7CKZ), with Pocket 1 highlighted in red indicating the primary ligand-binding region. **(B).** Predicted active site of the dopamine D3 receptor (7CMV), with Pocket 1 highlighted in red corresponding to the co-resolved ligand binding site.

Importantly, the location of Pocket 1 corresponded closely with the region where the co-resolved ligands were originally bound in the experimental structures of both receptors. This overlap suggests that the predicted binding pocket corresponds to the biologically relevant active site, thereby validating the reliability of the pocket prediction approach. The agreement between predicted and experimentally observed binding regions strengthens confidence in the accuracy of subsequent docking simulations performed within this site. For the D1 receptor this region corresponds to the inner-surface allosteric site occupied by LY3154207 (mevidalen) in 7CKZ rather than to the extracellular orthosteric pocket occupied by dopamine, whereas for the D3 receptor it corresponds to the orthosteric pocket occupied by PD-128907 in 7CMV. The D1 analyses presented in this study therefore characterize engagement of the allosteric region, and no orthosteric binding or agonist activity at the D1 receptor is claimed.

In addition to its positional relevance, Pocket 1 contained a sufficient number of residues with appropriate physicochemical properties, including hydrophobic and polar amino acids, which are essential for stabilizing ligand binding through a combination of hydrogen bonding and non-covalent interactions. The dimensions and coordinates of this pocket further supported its suitability for accommodating Rotigotine and its derivatives. Beside the pocket 1 other pockets were also identified which can be seen in Table 1.

**Table 1:** Active site and binding pocket information. Details of predicted active sites for D1 and D3 receptors, including Pocket 1 residues corresponding to the co-resolved ligand binding region.

No.	Score	Probability	No. of Residues	Avg Conservation	Dimensions		
					X	Y	Z
**D**_**1**_ **dopamine receptor (7CKZ)**							
**1**	8.33	0.489	20	1.915	7.7717	−2.0154	4.0452
**2**	3.56	0.137	14	0.647	4.1762	−2.3073	−22.8882
**3**	2.99	0.099	9	1.735	−5.7539	6.716	−8.9142
**4**	2.29	0.058	7	1.587	−6.5258	4.0509	−17.3827
**5**	2.25	0.055	9	1.788	−5.6364	−10.7837	−1.2645
**6**	2.05	0.044	8	1.166	−10.8155	−2.323	−25.616
**7**	1.73	0.03	7	0.895	8.1211	11.7738	−0.0096
**8**	1.66	0.027	10	1.341	8.4914	8.7378	−8.9406
**D**_**3**_ **dopamine receptor (7CMV)**							
**1**	23.19	0.87	26	0.726	93.8727	122.2154	117.8138
**2**	9.41	0.548	20	2.068	95.9131	112.2774	92.1244
**3**	2.65	0.079	10	1.700	82.5999	109.8115	101.866
**4**	2.12	0.048	10	1.454	105.9539	115.2881	105.841
**5**	1.67	0.027	6	1.666	91.7311	116.564	111.7908
**6**	1.58	0.024	6	2.936	96.6766	116.339	98.7636
**7**	1.23	0.012	6	1.038	90.5864	112.0011	126.6313

The confirmation that the predicted binding pocket aligns with the native ligand-binding region is a critical factor in structure-based drug design, as it ensures that docking studies are conducted within a functionally relevant site, thereby enhancing the biological relevance and reliability of subsequent interaction and binding affinity predictions.

### Molecular docking of D_1_ and D_3_ receptors with AI rotigotine derivatives

The molecular docking analysis revealed differential binding affinity patterns among Rotigotine and its AI-generated derivatives toward both the D1 and D3 dopamine receptors, highlighting the impact of structural modifications on receptor interaction. All predicted binding affinities are summarized in Table 2, providing a comprehensive comparison across the evaluated compounds.

**Table 2:** Predicted molecular docking binding affinities. Binding affinity values (kcal/mol) of rotigotine and AI-generated derivatives against D1 and D3 receptors.

Sr.	Compound	Binding Affinity (kcal/mol)
**D1 dopamine receptor (7CKZ)**		
**1.**	Rotigotine	−5.629
**2.**	AI Derivative 1	−5.744
**3.**	AI Derivative 2	−4.757
**4.**	AI Derivative 3	−5.489
**5.**	AI Derivative 4	−5.167
**6.**	AI Derivative 5	−5.631
**7.**	AI Derivative 6	−5.355
**8.**	AI Derivative 7	−5.486
**9.**	AI Derivative 8	−5.634
**10.**	AI Derivative 9	−5.011
**11.**	AI Derivative 10	−4.818
**12.**	AI Derivative 11	−5.458
**13.**	AI Derivative 12	−5.184
**14.**	AI Derivative 13	−5.132
**15.**	AI Derivative 14	−5.555
**16.**	Mevidalen	−7.285
**D3 dopamine receptor (7CMV)**		
**17.**	Rotigotine	−7.288
**18.**	AI Derivative 1	−7.934
**19.**	AI Derivative 2	−7.275
**20.**	AI Derivative 3	−6.789
**21.**	AI Derivative 4	−7.109
**22.**	AI Derivative 5	−7.196
**23.**	AI Derivative 6	−7.813
**24.**	AI Derivative 7	−6.979
**25.**	AI Derivative 8	−7.524
**26.**	AI Derivative 9	−7.046
**27.**	AI Derivative 10	−6.704
**28.**	AI Derivative 11	−7.127
**29.**	AI Derivative 12	−7.544
**30.**	AI Derivative 13	−6.965
**31.**	AI Derivative 14	−6.837
**32.**	PD-128907	−7.266

For the D1 dopamine receptor, AI Derivative 1 obtained the top-ranked docking score of −5.744 kcal/mol, compared with −5.629 kcal/mol for the reference compound Rotigotine. This difference of approximately 0.1 kcal/mol lies well within the intrinsic uncertainty of empirical docking scoring functions and is therefore not regarded as evidence of improved affinity; for the D1 receptor the two compounds are more appropriately described as indistinguishable in docking-based ranking. In contrast, AI Derivative 2 demonstrated the lowest predicted binding affinity at −4.757 kcal/mol, indicating comparatively weaker interaction and potentially reduced binding stability within the receptor site.

A similar trend was observed for the D3 dopamine receptor, where AI Derivative 1 again emerged as the top-performing compound with a predicted binding affinity of −7.934 kcal/mol, compared with −7.288 kcal/mol for Rotigotine. This larger score separation provides a comparatively stronger ranking signal for the D3 receptor, although it likewise reflects relative pose scoring rather than a quantitative difference in binding free energy. Conversely, AI Derivative 10 showed the lowest binding affinity among the evaluated compounds for the D3 receptor, with a value of −6.704 kcal/mol, indicating weaker predicted interaction strength.

To assess the reliability of the molecular docking workflow, re-docking validation of the native co-resolved ligands was performed for both dopamine receptor structures. The experimentally resolved ligands Mevidalen (D1 receptor, 7CKZ) and PD-128907 (D3 receptor, 7CMV) were re-docked into their respective binding pockets using the same docking parameters and grid coordinates applied during docking of Rotigotine and all AI-generated derivatives. The re-docked Mevidalen complex with the D1 receptor demonstrated a predicted binding affinity of −7.285 kcal/mol, while the re-docked PD-128907 complex with the D3 receptor showed a binding affinity of −7.266 kcal/mol.

The reliability of the molecular docking workflow was evaluated by re-docking the native co-resolved ligands into their experimentally defined binding pockets using the same grid coordinates, exhaustiveness, and sampling parameters applied during compound screening. Mevidalen was re-docked into the dopamine D1 receptor structure, whereas PD-128907 was re-docked into the dopamine D3 receptor structure. The predicted poses were compared with their corresponding experimentally resolved conformations using DockRMSD, which accounts for chemically equivalent and symmetric atoms during heavy-atom RMSD calculation. DockRMSD analysis produced an RMSD of 1.009 Å for Mevidalen and 1.359 Å for PD-128907. Both values were below the commonly applied 2.0 Å threshold, indicating acceptable reproduction of the experimentally observed ligand orientations. Visual superimposition further confirmed that both re-docked ligands occupied their respective native binding regions with orientations comparable to the experimental poses. These findings support the suitability of the selected grid coordinates and docking parameters for comparative screening of rotigotine and the CNN-generated derivatives against the D1 and D3 receptors. Nevertheless, successful pose reproduction validates the internal consistency of the docking protocol but does not independently confirm the predicted binding affinities or pharmacological activities of the screened compounds.

When comparing AI Derivative 1 with Rotigotine across both receptors, the derivative is ranked above the reference compound in both systems, with a larger score separation for D3 than for D1. Throughout this work, docking scores are used only as ranking and prioritization metrics for pose selection and lead triage; they are empirical scoring-function outputs rather than quantitative estimates of binding free energy, and small score differences, in particular the approximately 0.1 kcal/mol difference obtained for D1, are therefore not interpreted as evidence of improved affinity or optimized receptor compatibility. The better predicted affinity toward the D3 receptor compared to D1 across both compounds may suggest a potential receptor preference. The predicted binding affinities of all compounds can be seen in Table 2.

It is important to note that these binding affinities are computational predictions derived from docking simulations and should be interpreted as indicative rather than definitive measures of binding strength. Nevertheless, the observed trends provide a better basis for further analysis through interaction studies, pharmacophore evaluation, and molecular dynamics simulations to better understand the stability and behavior of these complexes under dynamic conditions.

### Molecular docking of D1 and D3 receptors with CNN-generated rotigotine derivatives

The molecular interaction analysis provided deeper insight into the binding behavior of the top-performing compound, AI Derivative 1, in comparison with the reference compound Rotigotine within the active sites of both D1 and D3 dopamine receptors. While visual representations of these interactions are illustrated in the 2D and 3D interaction diagrams and docking poses (Fig 3A**-**3D, Fig 4A**-**4D, and Fig 5A**-**5D), the detailed interaction profiles are more accurately interpreted from the interaction data summarized in **Table 3**.

**Table 3:** Molecular interaction details of ligand–receptor complexes. Summary of interacting residues, bond lengths, bond categories, and interaction types for docked complexes. For each interaction the interacting partner is given as the ligand atom (for contacts mapped to the ligand) or the receptor residue and atom name (for contacts mapped to the protein), using Discovery Studio Visualizer atom nomenclature. Hydrogen-bond rows specify the donor or acceptor atom involved; rows labelled as carbon hydrogen bonds denote weak C-H...O contacts rather than conventional hydrogen bonds.

Residue	Interacting atom / partner	Bond Category	Bond Type	Bond Length (Å)
**Derivative 1 with (D1 dopamine receptor 7CKZ)**				
**PHE333**	PHE333 (HN)...AI Derivative 1 (O)	Hydrogen Bond	Conventional Hydrogen Bond	2.49
**ILE217**	ILE217...AI Derivative 1	Hydrophobic	Alkyl	5.20
**ALA221**	ALA221...AI Derivative 1	Hydrophobic	Alkyl	5.46
**VAL270**	VAL270...AI Derivative 1	Hydrophobic	Alkyl	5.06
**LEU274**	LEU274...AI Derivative 1	Hydrophobic	Alkyl	4.76
**ARG121**	AI Derivative 1 (C)...ARG121	Hydrophobic	Alkyl	3.81
**Rotigotine with (D1 dopamine receptor 7CKZ)**				
**ILE125**	ILE125...Rotigotine	Hydrophobic	Alkyl	5.37
**ALA221**	ALA221...Rotigotine	Hydrophobic	Alkyl	4.36
**ILE225**	ILE225...Rotigotine	Hydrophobic	Alkyl	5.45
**ARG266**	ARG266...Rotigotine	Hydrophobic	Alkyl	4.9
**LYS269**	LYS269...Rotigotine	Hydrophobic	Alkyl	5.17
**VAL270**	VAL270...Rotigotine	Hydrophobic	Alkyl	5.24
**VAL270**	VAL270...Rotigotine	Hydrophobic	Alkyl	5.40
**VAL270**	VAL270...Rotigotine	Hydrophobic	Alkyl	4.95
**ILE217**	Rotigotine...ILE217	Hydrophobic	Alkyl	5.28
**LEU274**	Rotigotine...LEU274	Hydrophobic	Alkyl	4.59
**Derivative 1 with (D3 dopamine receptor 7CMV)**				
**ASP110**	AI Derivative 1 (H)...ASP110 (OD1)	Hydrogen Bond	Carbon Hydrogen Bond	2.38
**SER196**	SER196 (HB2)...AI Derivative 1 (O)	Hydrogen Bond	Carbon Hydrogen Bond	2.58
**CYS114**	AI Derivative 1 (C)...CYS114	Hydrophobic	Alkyl	4.22
**CYS114**	AI Derivative 1 (C)...CYS114	Hydrophobic	Alkyl	4.34
**VAL107**	VAL107...AI Derivative 1	Hydrophobic	Alkyl	4.93
**VAL111**	VAL111...AI Derivative 1	Hydrophobic	Alkyl	4.19
**PHE106**	PHE106...AI Derivative 1	Hydrophobic	Pi-Alkyl	5.48
**TRP342**	TRP342...AI Derivative 1 (C)	Hydrophobic	Pi-Alkyl	4.85
**PHE345**	PHE345...AI Derivative 1 (C)	Hydrophobic	Pi-Alkyl	4.08
**PHE345**	PHE345...AI Derivative 1 (C)	Hydrophobic	Pi-Alkyl	4.15
**PHE346**	PHE346...AI Derivative 1 (C)	Hydrophobic	Pi-Alkyl	4.33
**TYR373**	TYR373...AI Derivative 1 (C)	Hydrophobic	Pi-Alkyl	4.69
**Rotigotine with (D3 dopamine receptor 7CMV)**				
**ASP110**	Rotigotine (H31)...ASP110 (OD2)	Hydrogen Bond	Carbon Hydrogen Bond	2.93
**TYR373**	Rotigotine (H30)...TYR373 (OH)	Hydrogen Bond	Carbon Hydrogen Bond	3.07
**TYR365**	Rotigotine (S1)...TYR365	Other	Pi-Sulfur	5.61
**VAL107**	Rotigotine (C13)...VAL107	Hydrophobic	Alkyl	4.02
**ILE183**	Rotigotine (C13)...ILE183	Hydrophobic	Alkyl	4.72
**VAL111**	Rotigotine...VAL111	Hydrophobic	Alkyl	4.56
**VAL86**	VAL86...Rotigotine	Hydrophobic	Alkyl	4.71
**VAL111**	VAL111...Rotigotine	Hydrophobic	Alkyl	4.98
**CYS114**	CYS114...Rotigotine	Hydrophobic	Alkyl	5.38
**HIS349**	HIS349...Rotigotine	Hydrophobic	Pi-Alkyl	5.36

**Fig 3:**
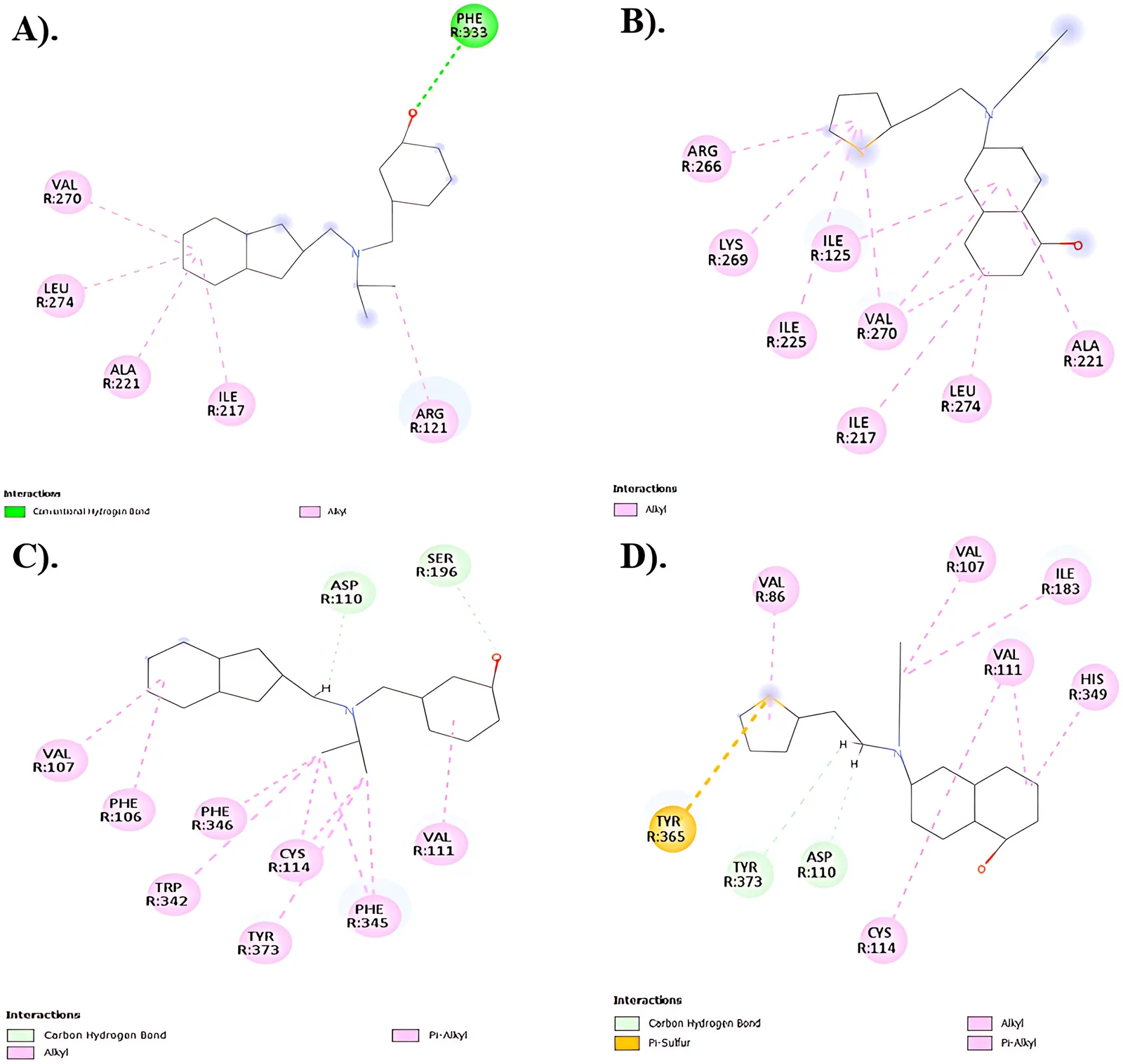
2D interaction analysis of ligand–receptor complexes. **(A).** AI Derivative 1 with D1 receptor, **(B).** Rotigotine with D1 receptor, **(C).** AI Derivative 1 with D3 receptor, and **(D).** Rotigotine with D3 receptor illustrating spatial binding interactions.

**Fig 4:**
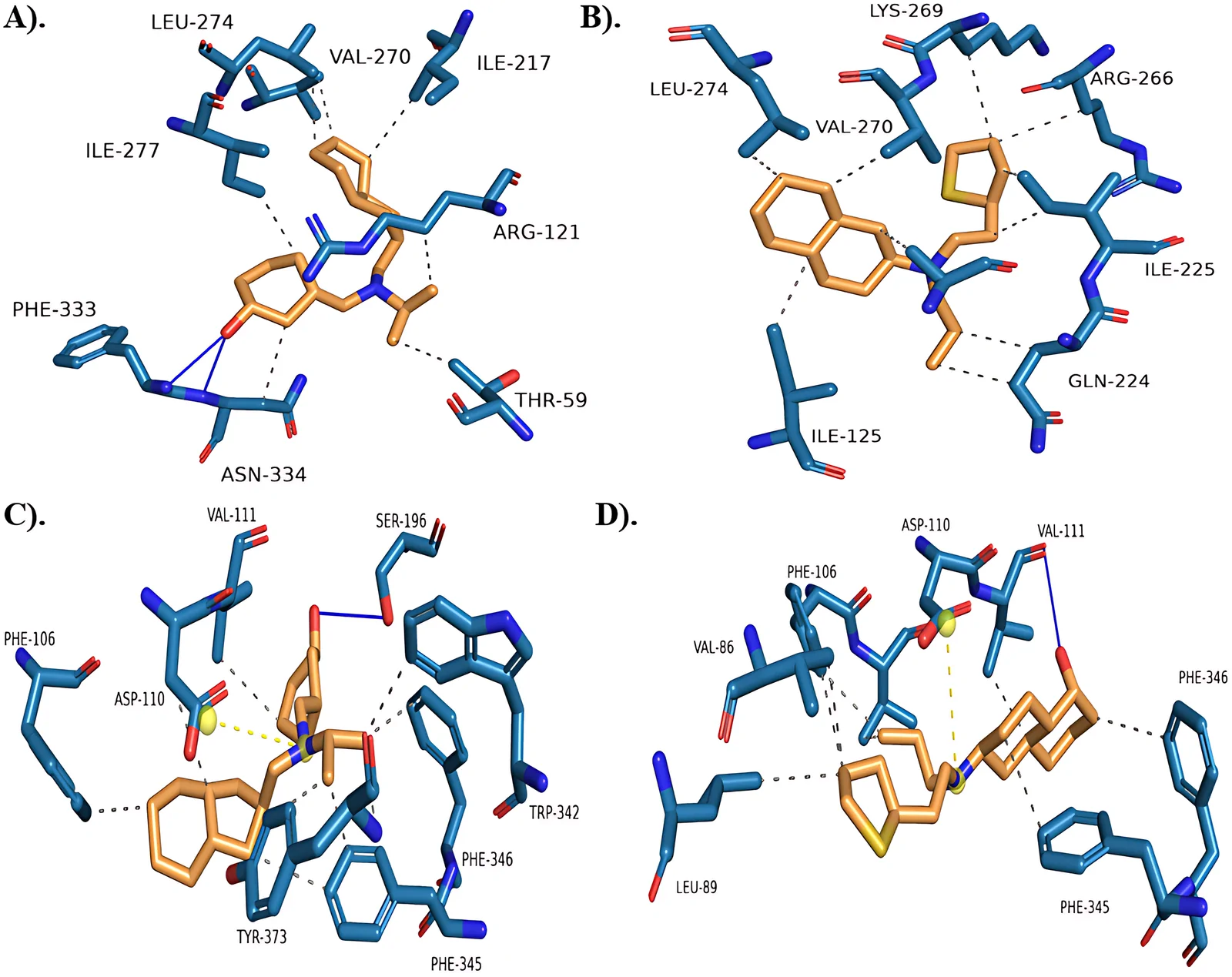
3D interaction analysis of ligand–receptor complexes. **(A).** AI Derivative 1 with D1 receptor, **(B).** Rotigotine with D1 receptor, **(C).** AI Derivative 1 with D3 receptor, and **(D).** Rotigotine with D3 receptor illustrating spatial binding interactions.

**Fig 5:**
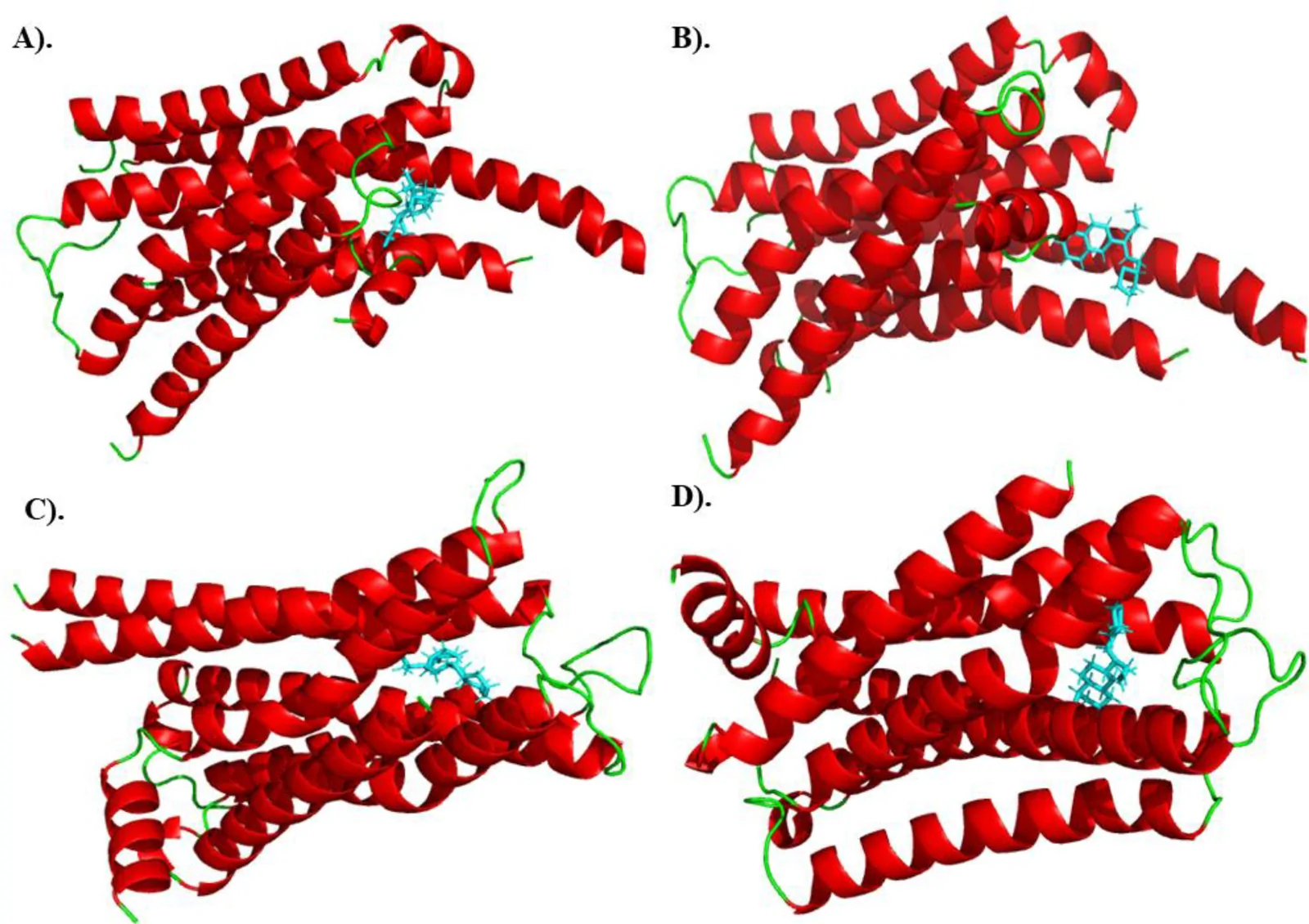
Docking poses of ligand–receptor complexes. **(A).** AI Derivative 1 with D1 receptor, **(B).** Rotigotine with D1 receptor, **(C).** AI Derivative 1 with D3 receptor, and **(D).** Rotigotine with D3 receptor showing binding orientation within the active site.

In the D1 dopamine receptor, AI Derivative 1 demonstrated a more diverse and potentially stabilizing interaction profile compared to Rotigotine. Notably, AI Derivative 1 formed a conventional hydrogen bond with the backbone amide of PHE333 (2.49 Å), the only conventional hydrogen bond observed across the four docked complexes. In addition to this polar interaction, multiple hydrophobic interactions were observed with residues such as ILE217, ALA221, VAL270, LEU274, and ARG121. These hydrophobic contacts may contribute to ligand stabilization within the binding pocket by enhancing van der Waals interactions and improving ligand accommodation. In contrast, Rotigotine predominantly exhibited hydrophobic interactions within the D1 receptor binding site, involving residues such as ILE125, ALA221, ILE225, ARG266, LYS269, VAL270, ILE217, and LEU274. While these interactions contribute to binding stabilization, the absence of a strong hydrogen bond interaction, as observed with AI Derivative 1, may explain its slightly lower predicted binding affinity. The repeated involvement of residues such as VAL270 and ILE217 in both ligands suggests that these residues play a central role in ligand anchoring within the D1 receptor.

For the D3 dopamine receptor, AI Derivative 1 again exhibited a more complex interaction pattern compared to Rotigotine. It formed carbon-hydrogen bond interactions with ASP110 and SER196. These are weak C-H...O contacts rather than conventional hydrogen bonds, and they are reported as directional polar contacts observed in the docked pose rather than as strong specificity determinants. Additionally, extensive hydrophobic interactions were observed with residues including CYS114, VAL107, VAL111, PHE106, TRP342, PHE345, PHE346, and TYR373. The presence of multiple π-alkyl interactions with aromatic residues may support improved ligand stabilization within the receptor cavity. Rotigotine, on the other hand, also formed hydrogen bond interactions with ASP110 and TYR373, but these were fewer in number and slightly longer in bond distance compared to those observed with AI Derivative 1. Its hydrophobic interactions involved residues such as VAL107, ILE183, VAL111, VAL86, CYS114, and HIS349. Additionally, a π-sulfur interaction with TYR365 was observed, which contributes to binding stabilization but may not compensate for the reduced number of hydrogen bonds compared to AI Derivative 1.

Overall, the interaction profile of AI Derivative 1 across both receptors suggests a potentially favorable balance of hydrogen bonding and hydrophobic interactions. The consistency of key interacting residues across both ligands indicates that the derivatives retain essential binding characteristics of the parent compound while introducing additional interactions that may enhance binding strength. The agreement between docking affinity trends and interaction profiles strengthens the reliability of these predictions. However, these findings remain computational in nature and should be interpreted as indicative of potential binding behavior, warranting further validation through dynamic simulations and experimental studies.

### Comparative pharmacophore characterization analysis

The pharmacophore characterization of Rotigotine and the selected lead compound, AI Derivative 1, revealed a conserved arrangement of key functional features, including two aromatic centers, one hydrogen bond donor, two hydrogen bond acceptors, and three hydrophobic regions (Fig 6A and 6B). This similarity indicates that the pharmacophoric framework of the parent scaffold is retained in the AI-generated derivative. The pharmacophore analysis is presented as a descriptive comparison of ligand feature composition and geometry; it is a ligand-based description and does not by itself establish receptor affinity, selectivity, or binding compatibility.

**Fig 6:**
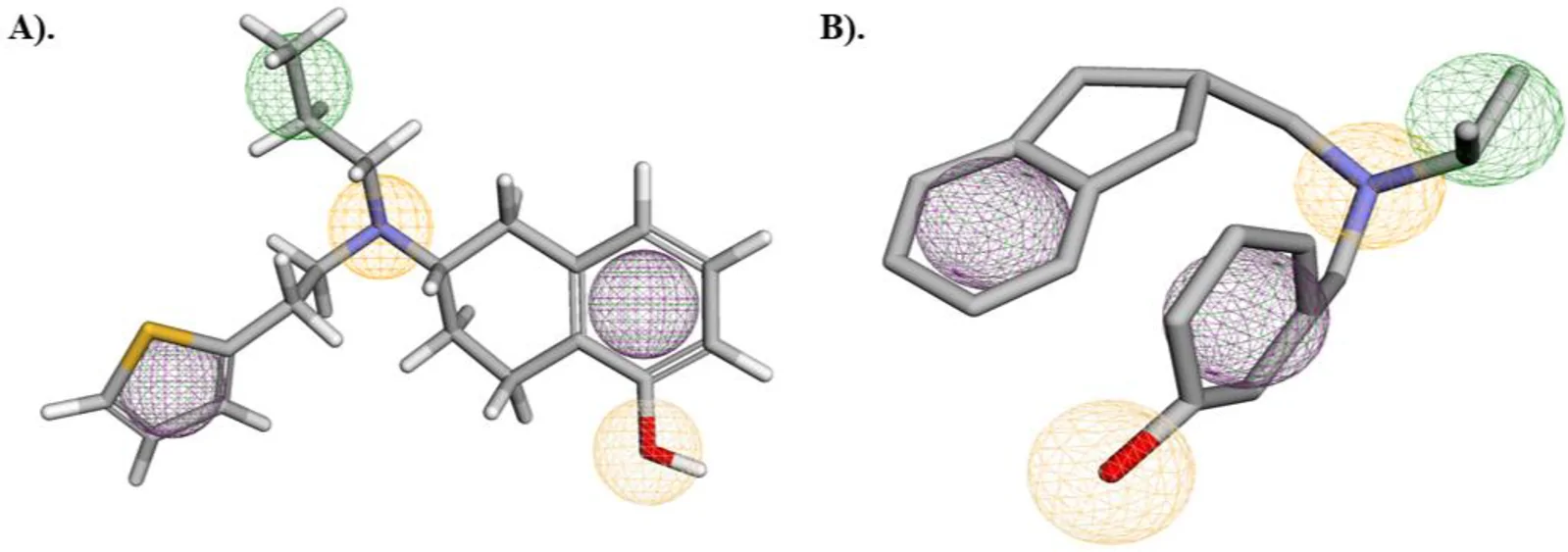
Pharmacophore characterization of ligands. **(A).** Rotigotine and **(B).** AI Derivative 1 showing key pharmacophoric features including hydrogen bond donors, acceptors, aromatic, and hydrophobic regions.

The two ligands carry an identical number and type of pharmacophoric features, and differ only in the spatial arrangement of those features. This is evident when correlating pharmacophore features with the molecular interaction profiles observed in both D1 and D3 receptors. The hydrogen-bond donor and acceptor features of AI Derivative 1 are compatible with the hydrogen bonds observed in the docked poses, namely with PHE333 in the D1 receptor and with ASP110 and SER196 in the D3 receptor, but an equal count of pharmacophoric features cannot be used to infer that these contacts are stronger than those formed by rotigotine. These interactions are consistent with the docking-based ranking of the derivative, although the pharmacophore features alone do not establish enhanced binding specificity or improved affinity.

In comparison, although Rotigotine possesses the same pharmacophoric elements, its interaction profile is dominated primarily by hydrophobic contacts, with fewer and less prominent hydrogen bonding interactions. This difference in the docked interaction profile is reported as an observation from the docking poses; no conclusion regarding binding efficiency is drawn from the pharmacophore comparison itself. The hydrophobic features identified in both compounds also correlate well with the extensive hydrophobic interactions observed in the docking analysis, particularly involving residues such as VAL, ILE, LEU, and aromatic residues in both receptors. Such hydrophobic contacts are commonly observed in aminergic receptor pockets and are reported here descriptively.

The consistency between pharmacophore features and observed interaction patterns supports the structural validity of the AI-generated derivative. It should be emphasized, however, that pharmacophore features are computed from the ligand alone and provide no measure of interaction strength. The pharmacophore results are therefore presented as descriptive molecular characterization and are not offered as evidence that AI Derivative 1 has improved receptor compatibility relative to the reference compound. However, as these findings are based on computational predictions, further validation through dynamic simulations and experimental studies is required to confirm the functional relevance of these interactions.

### Comparative DFT analysis

The density functional theory analysis was performed to characterize the intrinsic electronic properties of Rotigotine and AI Derivative 1 as isolated molecules, and is presented here as descriptive molecular characterization rather than as evidence relating to receptor binding. In the present study, AI Derivative 1 exhibited a dipole moment of 2.6370 Debye, whereas Rotigotine showed a lower dipole moment of 1.3727 Debye. This indicates that AI Derivative 1 has a more polarized ground-state charge distribution than Rotigotine, a property of the isolated molecule that does not by itself indicate stronger or more productive interactions within the receptor binding pocket. The dipole moments reported here were computed for the free ligands in the absence of the receptor and are therefore presented as descriptive electronic descriptors only.

The frontier molecular orbital analysis further distinguished the two compounds. Rotigotine showed a HOMO-LUMO energy gap of 0.20407 a.u., which corresponds to approximately 5.55 eV, whereas AI Derivative 1 exhibited a smaller energy gap of 0.18019 a.u., equivalent to approximately 4.90 eV. The smaller HOMO-LUMO gap indicates that AI Derivative 1 is the electronically softer and more polarizable of the two isolated molecules (Fig 7A and 7B). A smaller frontier orbital gap reflects intramolecular electronic softness and does not, by itself, demonstrate greater receptor affinity. No mechanistic relationship between the frontier orbital gap and the docking-based ranking is therefore claimed, and the two analyses are reported independently. A comparable descriptive combination of molecular docking and DFT in CNS-oriented computational screening has recently been reported [48].

**Fig 7:**
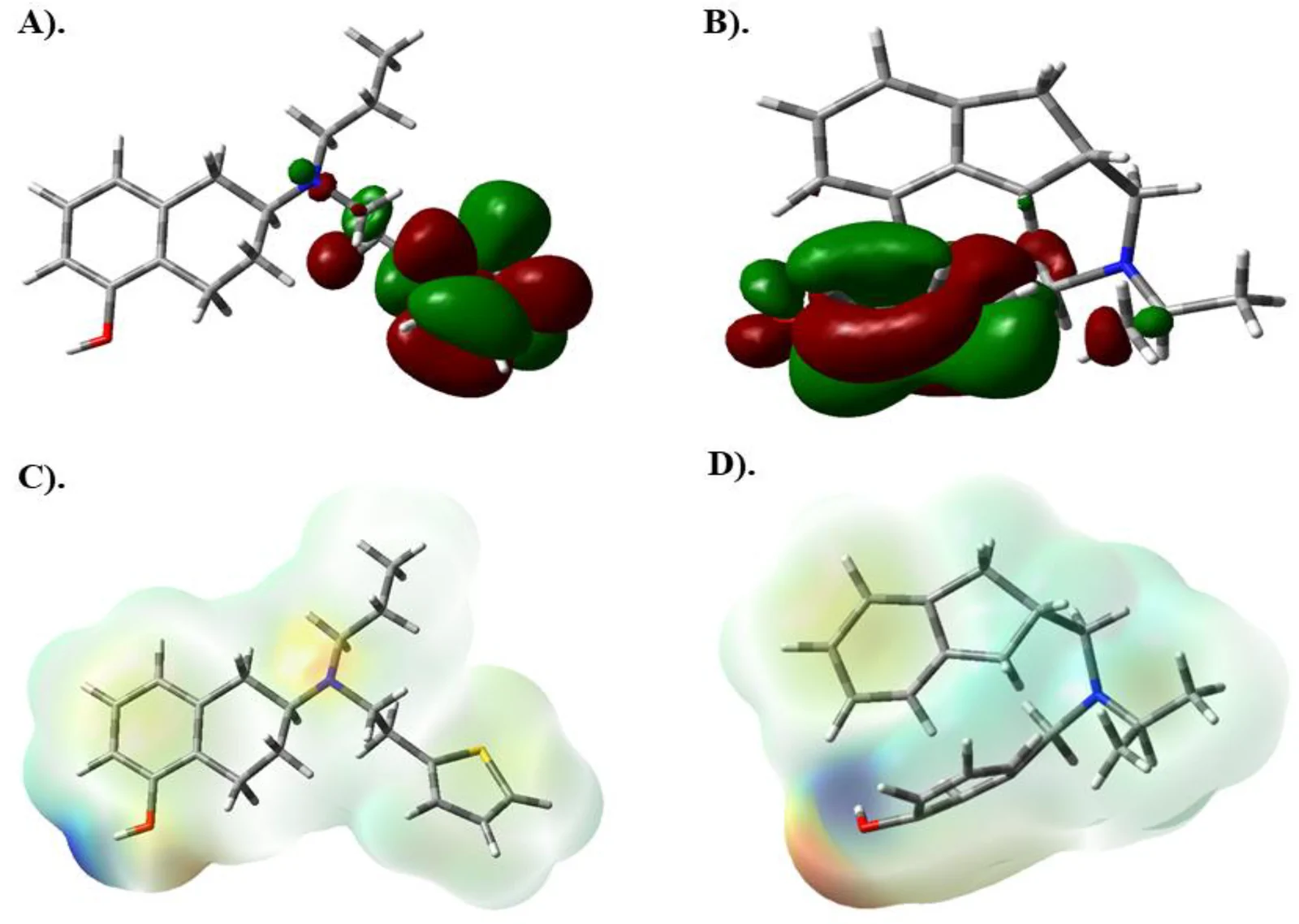
Density functional theory (DFT) analysis. **(A).** HOMO–LUMO gap of Rotigotine, **(B).** HOMO–LUMO gap of AI Derivative 1, **(C).** molecular electrostatic potential (MEP) of Rotigotine, and **(D).** MEP of AI Derivative 1.

The electronic descriptors and the docking results are reported separately and are not combined into a mechanistic explanation of binding. AI Derivative 1 was ranked above Rotigotine in docking and formed a more diverse predicted interaction profile, including hydrogen bonds in addition to hydrophobic contacts. The smaller HOMO-LUMO gap is not interpreted as a cause of these interaction differences, since frontier orbital energies computed for the free ligand carry no information about the receptor environment. Accordingly, no link is drawn between the electronic descriptors and the specific contacts observed with PHE333 in the D1 receptor or with ASP110 and SER196 in the D3 receptor. The larger energy gap of Rotigotine is likewise reported as an intrinsic electronic property and is not related here to its docking score or interaction pattern.

The molecular electrostatic potential maps are presented as a further descriptive characterization of the two isolated ligands. The MEP surface reflects the distribution of electron-rich and electron-deficient regions across the ligand and therefore helps identify sites that may participate in electrophilic or nucleophilic interactions during receptor binding. In Rotigotine, the electrostatic distribution appears relatively less polarized, suggesting a more moderate separation of reactive zones. (Fig 7C) In AI Derivative 1, however, the MEP pattern indicates a different distribution of electron-rich and electron-deficient surface regions (Fig 7D). Because these surfaces were computed for the isolated ligands in the absence of the receptor, they cannot be used to infer electrostatic complementarity with the D1 or D3 binding pockets.

Taken together, these calculations establish that the two ligands differ in polarity, frontier orbital gap, and electrostatic surface distribution. These are intrinsic molecular properties of the free ligands, and no inference is drawn from them regarding receptor recognition or accommodation. Accordingly, the DFT results are not presented as an additional line of evidence supporting a binding advantage for AI Derivative 1.

In summary, the DFT calculations describe AI Derivative 1 as having electronic properties that differ from those of Rotigotine, specifically a higher dipole moment, a smaller frontier orbital gap, and an altered electrostatic surface. These descriptors characterize the molecule but do not provide evidence of receptor affinity, selectivity, or biological activity. However, these interpretations remain predictive and should be considered supportive computational evidence rather than definitive proof of biological activity.

### Comparative in silico ADMET

The comparative ADMET analysis revealed that AI Derivative 1 retains an overall drug-like profile comparable to Rotigotine while showing several predicted improvements in physicochemical and safety-related parameters. Both compounds satisfied the major drug-likeness filters, including Lipinski, Ghose, Veber, Egan, and Mueggee criteria, with no violations, and both showed the same predicted bioavailability score of 0.55. This indicates that the AI-generated optimization did not compromise the general drug-like character of the parent scaffold and that the derivative remains within an acceptable range for oral drug-likeness from a computational perspective.

At the physicochemical level, AI Derivative 1 showed a lower molecular weight than Rotigotine, decreasing from 315.47 g/mol to 295.42 g/mol, while maintaining the same number of heavy atoms. It also displayed fewer rotatable bonds, decreasing from six to five, which may suggest slightly reduced conformational flexibility and potentially improved binding stability within a confined receptor pocket. A notable change was observed in topological polar surface area, which decreased from 51.71 Å2 in Rotigotine to 23.47 Å2 in AI Derivative 1. This reduction, together with a slightly lower consensus log Po/w value of 3.90 compared with 4.30 for Rotigotine, may indicate preservation of lipophilicity within a potentially favorable range while reducing overall polarity. Such a balance may be relevant for central nervous system-directed compounds because it could support membrane permeation without substantially compromising predicted solubility.

The solubility-related predictions also remained favorable. Both molecules were classified as moderately soluble, although AI Derivative 1 showed a slightly improved predicted solubility, with Log S improving from −4.85 to −4.55. Pharmacokinetic predictions further indicated high gastrointestinal absorption for both compounds and returned a binary “BBB permeant: Yes” classification for each. This binary output is an early-stage classification generated from simple physicochemical descriptors and is not evidence of adequate central nervous system exposure; predictive BBB models are strongly dependent on the applicability domain and on TPSA, lipophilicity, and ionization state, and require experimental confirmation [49]. Both were predicted to be non-substrates of P-glycoprotein, suggesting that active efflux may not strongly limit their central exposure. In the cytochrome-related predictions, AI Derivative 1 retained inhibition predictions for CYP1A2 and CYP2D6, similar to Rotigotine, but showed an improvement in CYP2C9 liability, where Rotigotine was predicted as an inhibitor whereas AI Derivative 1 was predicted as a non-inhibitor. This is a favorable shift because reduced CYP inhibition may suggest a lower risk of metabolic drug-drug interactions. Predictions for CYP2C19 and CYP3A4 remained unchanged between the two compounds. The detailed comparative ADME properties can be seen in Table 4.

**Table 4:** SwissADME analysis of Rotigotine and AI Derivative 1. Predicted physicochemical properties, pharmacokinetic parameters, and drug-likeness profiles of Rotigotine and AI Derivative 1.

ADME parameters	Rotigotine	AI Derivative 1
**Physicochemical Properties**		
**Formula**	C19H25NOS	C20H25NO
**Molecular weight**	315.47 g/mol	295.42 g/mol
**Num. heavy atoms**	22	22
**Num. arom. heavy atoms**	11	12
**Fraction Csp3**	0.47	0.40
**Num. rotatable bonds**	6	5
**Num. H-bond acceptors**	2	2
**Num. H-bond donors**	1	1
**Molar Refractivity**	95.19	92.51
**TPSA**	51.71 Å^2^	23.47 Å^2^
**Lipophilicity**		
**Consensus Log** ***P***_**o/w**_	4.30	3.90
**Water Solubility**		
**Log** ***S*** **(ESOL)**	−4.85	−4.55
**Class**	Moderately soluble	Moderately soluble
**Pharmacokinetics**		
**GI absorption**	High	High
**BBB permeant**	Yes	Yes
**P-gp substrate**	No	No
**CYP1A2 inhibitor**	Yes	Yes
**CYP2C19 inhibitor**	No	No
**CYP2C9 inhibitor**	Yes	No
**CYP2D6 inhibitor**	Yes	Yes
**CYP3A4 inhibitor**	No	No
**Log** ***K***_**p**_ **(skin permeation)**	−4.75 cm/s	−4.94 cm/s
**Drug-likeness**		
**Lipinski**	Yes; 0 violation	Yes; 0 violation
**Ghose**	Yes	Yes
**Veber**	Yes	Yes
**Egan**	Yes	Yes
**Muegge**	Yes	Yes
**Bioavailability Score**	0.55	0.55

The acute toxicity predictions differed between the two compounds. Rotigotine was predicted to have an LD_50_ of 250 mg/kg and to fall within toxicity class 3, whereas AI Derivative 1 was predicted to have a substantially higher LD_50_ of 753 mg/kg and to fall within toxicity class 4. In comparative toxicology terms, the higher LD_50_ and movement from class 3 to class 4 suggest a lower predicted acute oral toxicity for the AI-derived lead. The LD50 estimate and toxicity class are the ProTox outputs that carry information about predicted toxicity severity, and they are reported here as the primary acute-toxicity result.

The endpoint-specific ProTox predictions are summarized in Table 5. An important methodological point applies to their interpretation: the probability value returned by ProTox is a confidence estimate for the active or inactive classification, and it is not a measure of toxicity severity. A lower probability accompanying an “active” prediction therefore indicates lower confidence in that classification, not a weaker toxic effect, and the endpoint predictions are reported below on that basis. For hepatotoxicity, nephrotoxicity, cardiotoxicity, carcinogenicity, immunotoxicity, cytotoxicity, and nutritional toxicity, both compounds were predicted to be inactive with generally acceptable probabilities. The most important difference concerns neurotoxicity. Rotigotine was predicted inactive (probability 0.73) whereas AI Derivative 1 was predicted active (probability 0.64). This is an active neurotoxicity alert for the derivative and is reported here as such. The associated probability reflects only the model confidence in the active classification and says nothing about the severity of the predicted effect; the alert therefore cannot be discounted on the basis of its probability value. Both compounds were predicted active for respiratory toxicity, with classification confidence values of 0.88 for AI Derivative 1 and 0.97 for Rotigotine. Both are active alerts, and the lower value for the derivative indicates lower confidence in the classification, not a weaker toxic effect. For mutagenicity, both compounds were predicted active, with confidence values close to the decision threshold, 0.52 for Rotigotine and 0.51 for AI Derivative 1. Because these values lie close to the decision threshold, the classification itself is uncertain for both compounds, and neither ligand can be described as less mutagenic than the other.

**Table 5:** ProTox-3.0 oral toxicity prediction. Toxicity classes, and toxicity endpoints of Rotigotine and AI Derivative 1.

Category	Target	Rotigotine		AI Derivative 1	
		Prediction	Probability	Prediction	Probability
**Organ toxicity**	**Hepatotoxicity**	**Inactive**	**0.92**	**Inactive**	**0.91**
	**Neurotoxicity**	**Inactive**	**0.73**	**Active**	**0.64**
	**Nephrotoxicity**	**Inactive**	**0.78**	**Inactive**	**0.84**
	**Respiratory toxicity**	**Active**	**0.97**	**Active**	**0.88**
	**Cardiotoxicity**	**Inactive**	**0.75**	**Inactive**	**0.73**
**Toxicity end points**	**Carcinogenicity**	**Inactive**	**0.61**	**Inactive**	**0.66**
	**Immunotoxicity**	**Inactive**	**0.97**	**Inactive**	**0.86**
	**Mutagenicity**	**Active**	**0.52**	**Active**	**0.51**
	**Cytotoxicity**	**Inactive**	**0.57**	**Inactive**	**0.65**
	**BBB-barrier**	**Active**	**0.88**	**Active**	**0.88**
	**Ecotoxicity**	**Active**	**0.65**	**Active**	**0.64**
	**Clinical toxicity**	**Active**	**0.56**	**Active**	**0.55**
	**Nutritional toxicity**	**Inactive**	**0.59**	**Inactive**	**0.74**
**Tox21-Nuclear receptor signaling pathways**	**Aryl hydrocarbon Receptor (AhR)**	**Inactive**	**0.90**	**Inactive**	**0.65**
	**Androgen Receptor (AR)**	**Inactive**	**0.98**	**Inactive**	**0.86**
	**Androgen Receptor Ligand Binding Domain (AR-LBD)**	**Inactive**	**0.94**	**Inactive**	**0.97**
	**Aromatase**	**Inactive**	**0.90**	**Inactive**	**0.85**
	**Estrogen Receptor Alpha (ER)**	**Inactive**	**0.91**	**Inactive**	**0.77**
	**Estrogen Receptor Ligand Binding Domain (ER-LBD)**	**Inactive**	**0.97**	**Inactive**	**0.93**
	**Peroxisome Proliferator Activated Receptor Gamma (PPAR-Gamma)**	**Inactive**	**0.98**	**Inactive**	**0.95**
	**Nuclear factor (erythroid-derived 2)-like 2/ antioxidant responsive element (nrf2/ARE)**	**Inactive**	**0.94**	**Inactive**	**0.87**
	**Heat shock factor response element (HSE)**	**Inactive**	**0.94**	**Inactive**	**0.87**
	**Mitochondrial Membrane Potential (MMP)**	**Inactive**	**0.73**	**Inactive**	**0.72**
	**Phosphoprotein (Tumor Supressor) p53**	**Inactive**	**0.91**	**Inactive**	**0.84**
	**ATPase family AAA domain-containing protein 5 (ATAD5)**	**Inactive**	**0.98**	**Inactive**	**0.97**
	**Thyroid hormone receptor alpha (THRα)**	**Inactive**	**0.71**	**Inactive**	**0.69**
**Molecular Initiating**	Thyroid hormone receptor beta (THRβ)	Inactive	0.73	Inactive	0.71
	Transtyretrin (TTR)	Inactive	0.89	Inactive	0.80
	Ryanodine receptor (RYR)	Inactive	0.62	Inactive	0.90
	GABA receptor (GABAR)	Inactive	0.73	Inactive	0.52
	Glutamate N-methyl-D-aspartate receptor (NMDAR)	Inactive	0.84	Inactive	0.75
	alpha-amino-3-hydroxy-5-methyl-4-isoxazolepropionate receptor (AMPAR)	Inactive	1.00	Inactive	0.99
	Kainate receptor (KAR)	Inactive	1.00	Inactive	1.00
	Achetylcholinesterase (AChE)	Active	0.53	Inactive	0.56
	Constitutive androstane receptor (CAR)	Inactive	0.99	Inactive	0.99
	Pregnane X receptor (PXR)	Inactive	0.77	Inactive	0.74
	NADH-quinone oxidoreductase (NADHOX)	Inactive	0.84	Inactive	0.86
	Voltage gated sodium channel (VGSC)	Inactive	0.84	Inactive	0.84
	Na+/I- symporter (NIS)	Inactive	0.91	Inactive	0.96
**Metabolism**	Cytochrome CYP1A2	Inactive	0.80	Inactive	0.62
	Cytochrome CYP2C19	Inactive	0.83	Inactive	0.72
	Cytochrome CYP2C9	Inactive	0.70	Inactive	0.65
	Cytochrome CYP2D6	Active	0.93	Active	0.85
	Cytochrome CYP3A4	Inactive	0.82	Inactive	0.86
	Cytochrome CYP2E1	Inactive	0.99	Inactive	0.99

Blood-brain barrier activity was predicted active for both compounds with identical classification confidence values of 0.88. This is an early-stage binary classification only and does not establish adequate central nervous system exposure for either compound, Ecotoxicity and clinical toxicity were also predicted active for both molecules. The confidence values were slightly lower for AI Derivative 1, which indicates lower certainty in the active classification and cannot be read as a reduced toxicity risk; both compounds carry active alerts for these endpoints. Within the molecular initiating event panel, Rotigotine was predicted active against acetylcholinesterase with a probability of 0.53, whereas AI Derivative 1 was predicted inactive with a probability of 0.56. This is a favorable change because it suggests that the AI derivative may have a lower predicted tendency for off-target cholinergic interaction. In the metabolism-related predictions, both compounds were predicted active for CYP2D6, with a somewhat lower classification confidence for AI Derivative 1; both are active predictions and no difference in metabolic liability is inferred.

Taken together, the ADMET and toxicity comparisons suggest that AI Derivative 1 preserves the desirable central nervous system-oriented pharmacokinetic profile of Rotigotine while showing several computationally favorable shifts, including lower molecular weight, fewer rotatable bonds, improved solubility, loss of predicted CYP2C9 inhibition, and a markedly improved acute toxicity profile with a higher LD_50_ and a less severe toxicity class. These favorable shifts must, however, be read alongside the active alerts that both compounds retain. AI Derivative 1 carries active predictions for neurotoxicity, respiratory toxicity, mutagenicity, ecotoxicity, and clinical toxicity, and the associated probability values express confidence in those classifications rather than the severity of the predicted effects. The derivative is therefore not described as generally safer than rotigotine; the defensible statement is that it has a higher predicted oral LD50 and a less severe predicted acute toxicity class, while retaining several active toxicity alerts, including an active neurotoxicity alert that rotigotine does not carry. It should also be noted that rotigotine is administered clinically as a transdermal formulation, whereas the endpoints reported here, including gastrointestinal absorption, oral LD50, and oral toxicity class, describe oral administration. These predictions are therefore reported as generic physicochemical and toxicity descriptors for compound triage rather than as a pharmacokinetic assessment of the intended clinical route, which for a rotigotine-derived candidate would most plausibly remain transdermal. Considered together with the docking-based ranking, the ADMET predictions are consistent with continued computational interest in AI Derivative 1, without establishing an improved safety profile. However, these remain predictive *in silico* findings and require experimental pharmacokinetic and toxicological validation before any firm conclusion can be made about real biological safety or therapeutic suitability.

### Comparative molecular dynamics simulation of AI lead and Rotigotine

#### Root Mean Square Deviation (RMSD) analysis.

The RMSD analysis was performed to evaluate the structural stability of the protein–ligand complexes over the 500 ns simulation period and to determine the equilibration behavior of each system (Fig 8A and 8B). Before analysis, all trajectories were post-processed using standard GROMACS utilities for whole-molecule reconstruction, removal of periodic-image jumps, centering of the receptor within the box, and least-squares fitting of the receptor backbone to the starting structure, so that the reported RMSD, RMSF, and radius-of-gyration values are not generated by molecules crossing periodic boundaries. For the AI Derivative 1–D1 receptor (7CKZ) complex, the RMSD increased from approximately 0.2 nm to 0.5 nm within the initial 80–100 ns, indicating early structural adaptation. A significant fluctuation phase was observed between 100 ns and 180 ns, where RMSD values increased up to approximately 1.6–1.7 nm. After this transitional phase, the system stabilized around 200 ns and maintained a consistent RMSD range of approximately 0.8–0.9 nm until the end of the simulation. This behavior suggests that although the system underwent an initial conformational rearrangement, it achieved a stable binding conformation thereafter.

**Fig 8:**
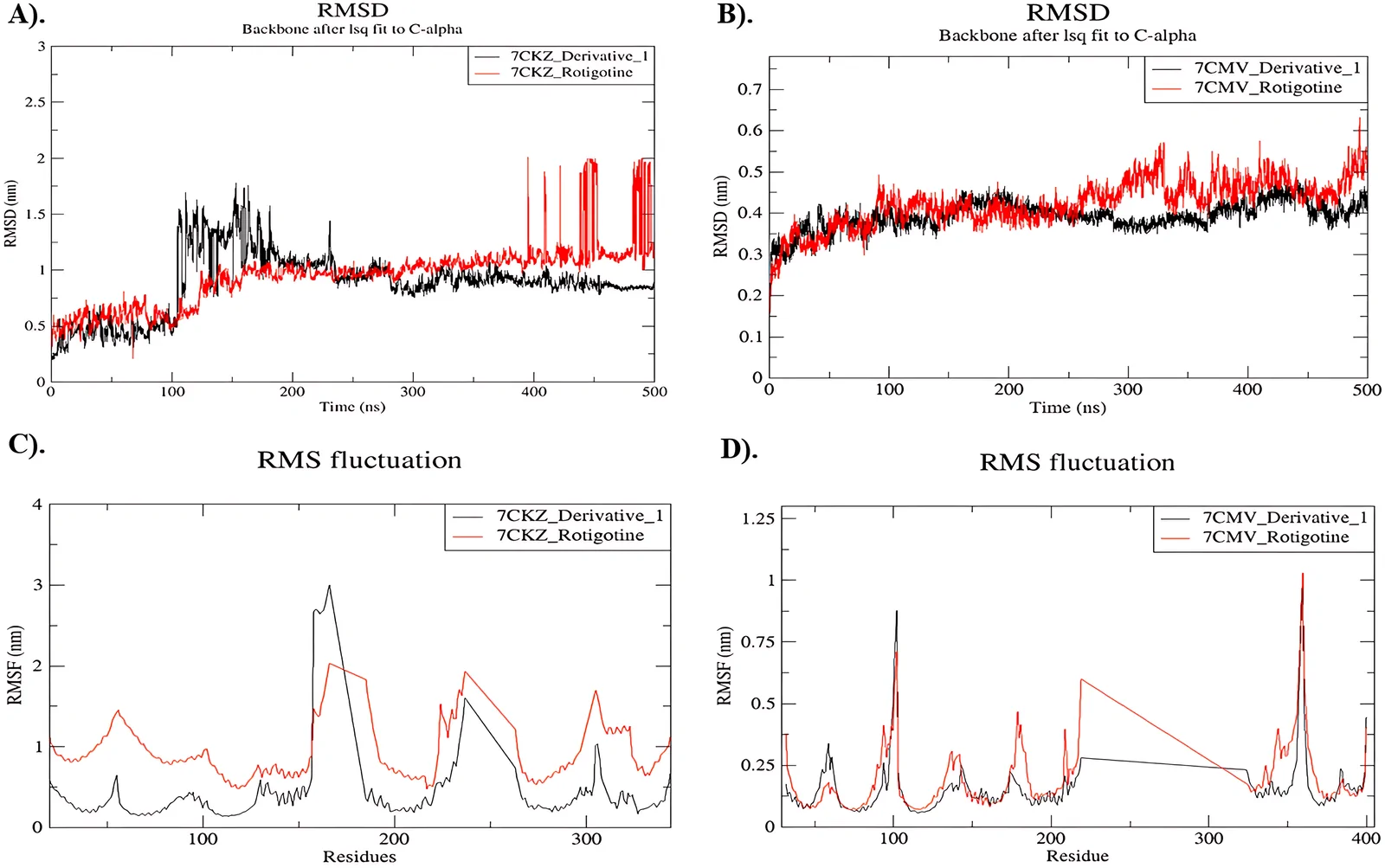
Comparative RMSD and RMSF analysis of protein–ligand complexes. **(A).** Comparative RMSD of AI Derivative 1 and Rotigotine with D1 receptor, **(B).** comparative RMSD of AI Derivative 1 and Rotigotine with D3 receptor, **(C).** comparative RMSF of AI Derivative 1 and Rotigotine with D1 receptor, and **(D).** comparative RMSF of AI Derivative 1 and Rotigotine with D3 receptor.

In comparison, the Rotigotine–D1 receptor (7CKZ) complex showed a gradual increase in RMSD from approximately 0.5 nm to around 1.0 nm within the first 150 ns. The system appeared relatively stable between 150 ns and 350 ns, maintaining RMSD values around 1.0–1.1 nm. However, after approximately 400 ns, notable fluctuations were observed, with RMSD spikes reaching up to approximately 2.0 nm. Deviations of this magnitude are too large to be interpreted as a ligand-dependent difference in binding stability. An RMSD approaching 2.0 nm for a seven-transmembrane receptor implies a substantial rearrangement of the helical bundle itself, which is the expected behavior of a transmembrane domain simulated in bulk water without a lipid bilayer. This feature is therefore reported as a property of the simulation environment rather than as evidence of weaker rotigotine binding. For the AI Derivative 1–D3 receptor (7CMV) complex, the RMSD profile demonstrated better stability throughout the simulation. The RMSD increased slightly from approximately 0.3 nm to 0.4 nm within the first 100 ns and remained consistently stable within the range of 0.35–0.45 nm for the entire 500 ns duration. This trajectory therefore showed the smallest structural deviation of the four systems examined.

The Rotigotine–D3 receptor (7CMV) complex also exhibited relatively stable behavior; however, it showed slightly higher deviations compared to the AI derivative. The RMSD increased from approximately 0.25 nm to 0.4 nm in the early phase and gradually rose to approximately 0.5–0.6 nm toward the end of the simulation. Minor fluctuations persisted throughout the trajectory. Overall, both D3 trajectories remained within a comparable and relatively narrow deviation range. These differences are reported descriptively; because only a single trajectory was generated for each system, no comparative claim of improved structural stability is made.

#### Root Mean Square Fluctuation (RMSF) analysis.

The RMSF analysis was performed to assess residue-level flexibility and to identify regions of structural mobility within the receptor during simulation (Fig 8C and 8D). For the AI Derivative 1–D1 receptor (7CKZ) complex, most residues exhibited low fluctuations, generally below 0.5 nm, indicating stable backbone behavior. A fluctuation peak of approximately 3.0 nm was observed around residues 160–180. A per-residue fluctuation of this magnitude greatly exceeds the range expected for a flexible loop in a folded receptor and instead indicates that this segment became largely unstructured during the trajectory, consistent with the absence of a stabilizing lipid environment. Outside this region the fluctuations remained low, but this observation is reported descriptively and is not interpreted as evidence of ligand-mediated stabilization.

In contrast, the Rotigotine–D1 receptor (7CKZ) complex displayed higher overall fluctuations across multiple regions. RMSF values frequently ranged between 0.8 and 2.0 nm. Fluctuations of this magnitude across multiple regions indicate extensive loss of the folded receptor architecture in this trajectory rather than a ligand-specific difference in binding. They are therefore not interpreted as evidence of less stable rotigotine binding. For the AI Derivative 1–D3 receptor (7CMV) complex, the RMSF values were consistently low, with most residues fluctuating below 0.3 nm. A moderate peak was observed around residues 90–100, reaching approximately 0.85 nm, indicating localized flexibility. Overall, the receptor maintained a stable conformation with minimal structural perturbation. The Rotigotine–D3 receptor (7CMV) complex exhibited comparatively higher fluctuations, particularly around residues 90–100 and near residue 350, where RMSF values reached approximately 1.0 nm. These differences are reported descriptively and are not used to rank the two ligands.

#### Radius of Gyration (Rg) analysis.

The radius of gyration analysis was performed to evaluate the compactness and overall structural integrity of the protein–ligand complexes throughout the simulation (Fig 9A and 9B). For the AI Derivative 1–D1 receptor (7CKZ) complex, the Rg values remained relatively stable around 2.1–2.2 nm for the majority of the simulation. A slight increase was observed between approximately 100 ns and 170 ns, where Rg reached around 2.3–2.4 nm, corresponding to the structural adjustment phase also observed in RMSD. After approximately 200 ns, the system stabilized and maintained a consistent compact structure without significant deviations until 500 ns. This indicates that the protein retained its folded conformation and structural integrity in the presence of the AI derivative.

**Fig 9:**
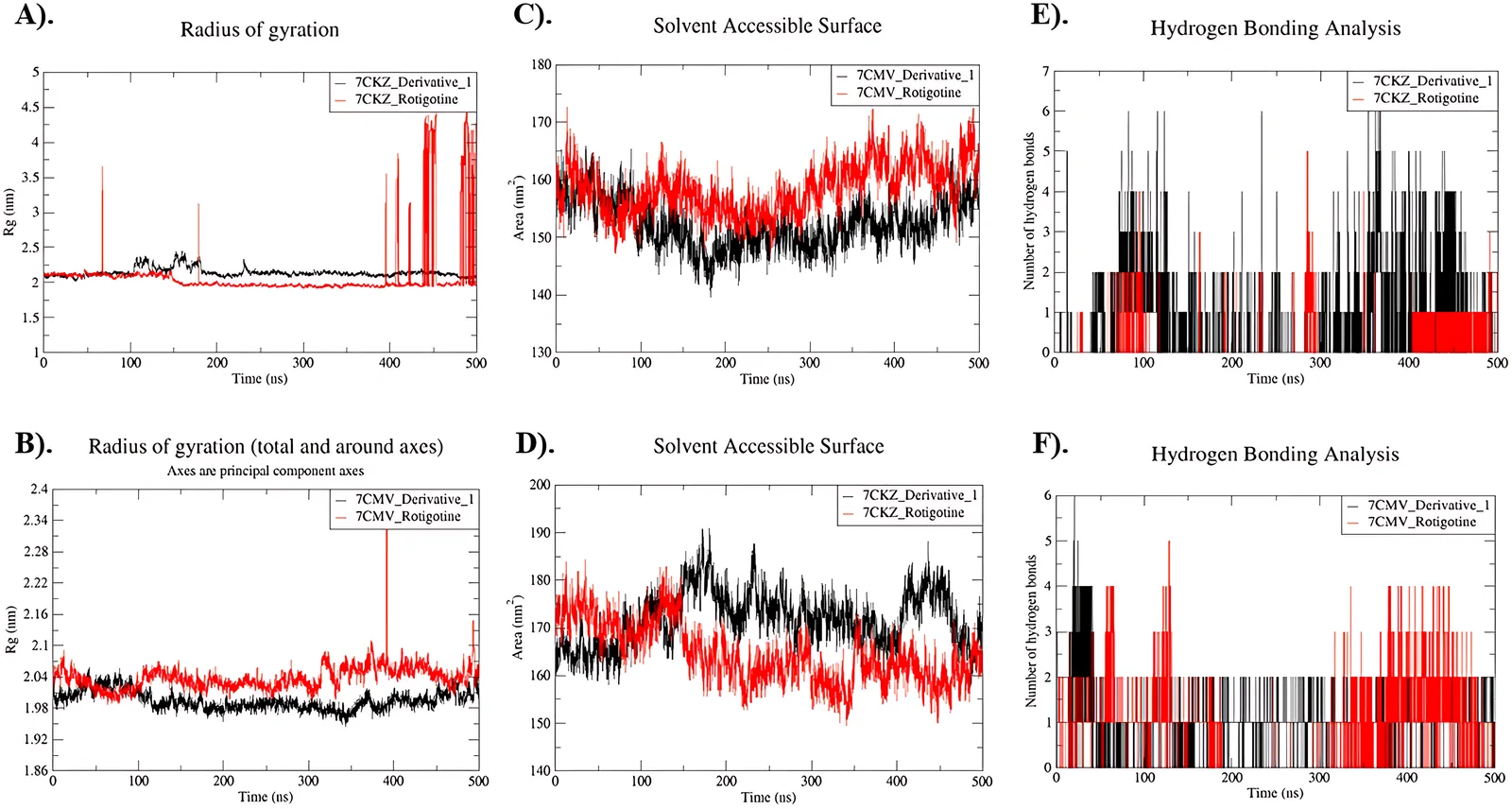
Rg, SASA, and hydrogen bond analysis of protein–ligand complexes. **(A).** Radius of gyration (Rg) of AI Derivative 1 and Rotigotine with the D1 receptor, **(B).** Rg of AI Derivative 1 and Rotigotine with the D3 receptor, **(C).** solvent accessible surface area (SASA) of AI Derivative 1 and Rotigotine with the D1 receptor, **(D).** SASA of AI Derivative 1 and Rotigotine with the D3 receptor, **(E).** hydrogen bonds of AI Derivative 1 and Rotigotine with the D1 receptor, and **(F).** hydrogen bonds of AI Derivative 1 and Rotigotine with the D3 receptor. Each panel compares the two ligands within the same receptor system.

In contrast, the Rotigotine–D1 receptor (7CKZ) complex showed lower Rg values overall, stabilizing around 1.95–2.0 nm for a large portion of the simulation. However, after approximately 400 ns, substantial fluctuations were observed, with Rg values abruptly increasing up to approximately 4.0–4.4 nm. An increase in the radius of gyration from approximately 2.0 nm to 4.0–4.4 nm corresponds to an approximate doubling of the overall dimensions of the complex and cannot represent a physiologically meaningful conformational change of a folded G protein-coupled receptor. It is consistent with partial unfolding or separation of the transmembrane helices in bulk water, and a residual trajectory-processing artifact affecting the later part of this single trajectory cannot be excluded. This behavior is therefore reported as a feature of the non-membrane simulation setup, and no conclusion regarding rotigotine binding stability is drawn from it.

For the AI Derivative 1–D3 receptor (7CMV) complex, the Rg remained consistently stable throughout the simulation, fluctuating within a narrow range of approximately 1.97–2.02 nm. This indicates a highly compact and stable protein structure with minimal conformational deviation. No major fluctuations were observed, suggesting that the ligand maintained a stable binding conformation without disturbing the overall protein architecture.

The Rotigotine–D3 receptor (7CMV) complex also showed relatively stable Rg values, ranging between approximately 2.02 and 2.08 nm. However, a noticeable spike was observed around 380 ns, where Rg briefly increased to approximately 2.35 nm before returning to baseline values. Although this fluctuation is less severe than that observed in the D1 system, it is reported here as a feature of this single non-membrane trajectory. The Rg profiles are therefore described individually for each system and are not used to rank the two ligands by structural compactness or dynamic stability.

#### Solvent Accessible Surface Area (SASA) analysis.

The solvent accessible surface area analysis was used to evaluate the exposure of the protein surface to the solvent environment and to assess structural compactness and folding behavior during the simulation (Fig 9C and 9D). For the AI Derivative 1–D1 receptor (7CKZ) complex, SASA values initially ranged around 162–165 nm^2^ and gradually increased to approximately 175–180 nm^2^ between 100 ns and 180 ns, indicating increased solvent exposure during the structural adjustment phase. After approximately 200 ns, the system stabilized with SASA values fluctuating around 170–175 nm^2^, suggesting a stable balance between compactness and solvent exposure.

In comparison, the Rotigotine–D1 receptor (7CKZ) complex showed lower SASA values overall, generally ranging between 155 and 170 nm^2^. However, significant fluctuations were observed, particularly after 350–400 ns, where the system displayed irregular variations in surface exposure. These fluctuations correspond with the instability observed in RMSD and Rg analyses, indicating structural inconsistency and possible partial unfolding or rearrangement in the later stages of simulation. For the AI Derivative 1–D3 receptor (7CMV) complex, SASA values remained consistently lower and stable, fluctuating between approximately 145 and 155 nm^2^ throughout the simulation. A slight decrease was observed around 150–200 ns, followed by stabilization, indicating a well-maintained compact structure with minimal solvent exposure changes.

The Rotigotine–D3 receptor (7CMV) complex exhibited higher SASA values compared to the AI derivative, generally ranging between 150 and 165 nm^2^. Gradual increases in SASA were observed after approximately 300 ns, indicating increased solvent exposure and reduced compactness relative to the AI derivative complex. Overall, the SASA profiles differed between the four systems; because each system was sampled in a single non-membrane trajectory, these differences are reported descriptively and are not interpreted as evidence that either ligand maintains a more stable or more compact protein environment.

#### Hydrogen bond analysis.

Hydrogen bond analysis was performed to evaluate the stability and persistence of intermolecular interactions between the ligand and receptor throughout the simulation (Fig 9E and 9F). For the AI Derivative 1–D1 receptor (7CKZ) complex, the number of hydrogen bonds varied dynamically throughout the simulation, typically ranging between 1 and 4 bonds, with occasional peaks reaching up to 5–6 hydrogen bonds. Notably, after approximately 350 ns, the system maintained a relatively consistent hydrogen bonding pattern with 2–4 hydrogen bonds, indicating stable ligand–receptor interactions over time.

In contrast, the Rotigotine–D1 receptor (7CKZ) complex showed fewer and less consistent hydrogen bonds. The number of hydrogen bonds generally fluctuated between 0 and 2, with occasional transient peaks reaching up to 4–5 bonds. After approximately 400 ns, the system exhibited reduced hydrogen bonding persistence, often maintaining only 0–1 hydrogen bond, indicating weaker and less stable interactions compared to the AI derivative.

For the AI Derivative 1–D3 receptor (7CMV) complex, hydrogen bond formation was relatively stable, with the system consistently maintaining 1–3 hydrogen bonds throughout the simulation. Occasional early peaks of up to 4–5 hydrogen bonds were observed within the first 50 ns, followed by stabilization, indicating sustained interaction with the receptor. The Rotigotine–D3 receptor (7CMV) complex exhibited more variability in hydrogen bonding, with values fluctuating between 0 and 3 bonds and intermittent peaks reaching up to 4 hydrogen bonds, particularly after 350 ns. However, these interactions were less consistent over time compared to the AI derivative, indicating weaker interaction persistence. The hydrogen bond counts are reported as descriptive observations from single non-membrane trajectories. Hydrogen-bond occupancy was not quantified and replicate trajectories were not performed, so these profiles are not interpreted as evidence of improved binding stability for either compound under dynamic conditions.

#### Dynamic Cross-Correlation Matrix (DCCM) analysis.

The dynamic cross-correlation matrix analysis was performed to investigate the correlated and anti-correlated motions between residues within the protein during the simulation (Fig 10A-10D). Positive correlation values indicate coordinated movements in the same direction, whereas negative values represent opposite or anti-correlated motions, which are often associated with structural flexibility or instability.

**Fig 10:**
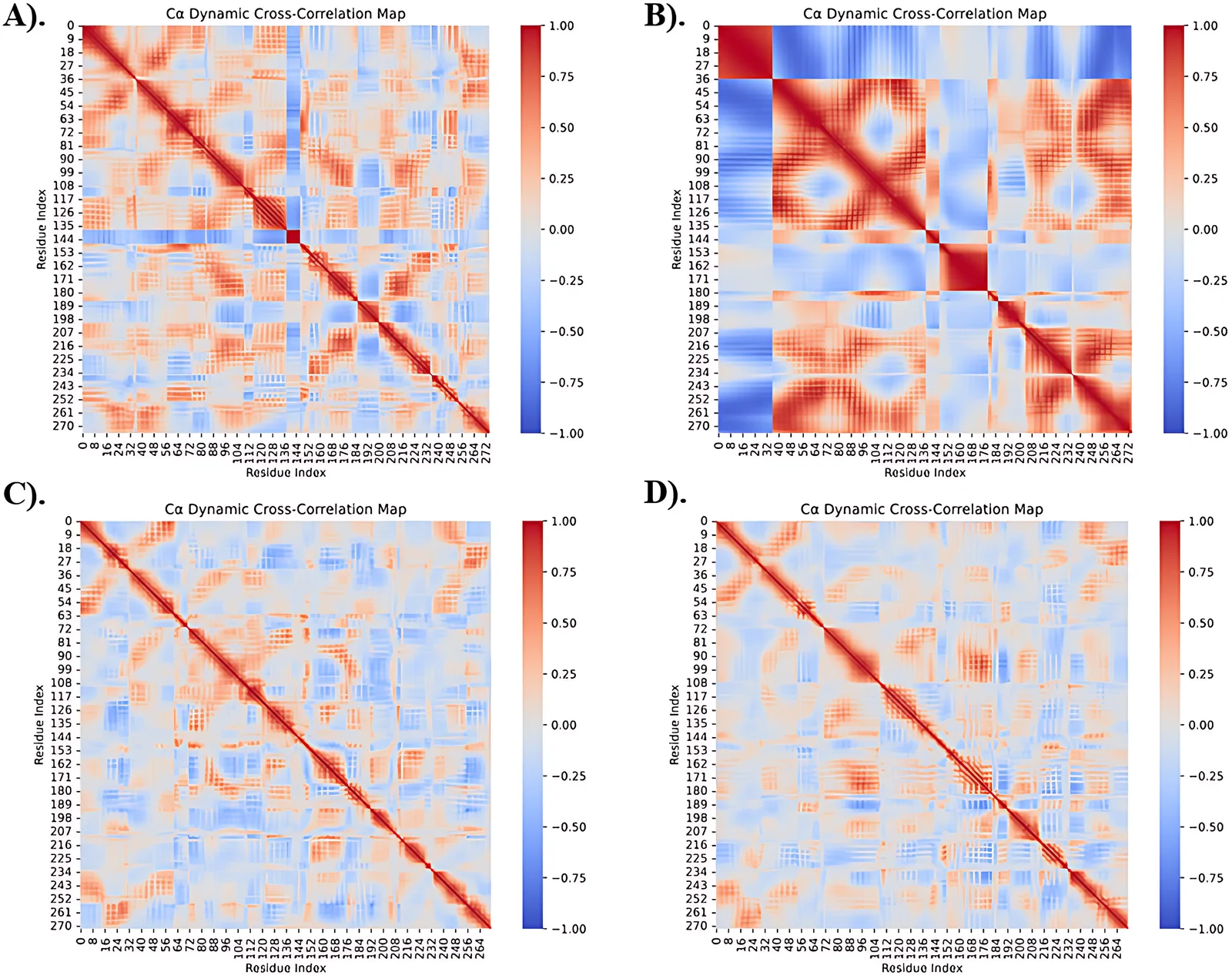
Dynamic cross-correlation matrix (DCCM) analysis. **(A).** AI Derivative 1 with D1 receptor, **(B).** Rotigotine with D1 receptor, **(C).** AI Derivative 1 with D3 receptor, and **(D)**. Rotigotine with D3 receptor showing correlated and anti-correlated residue motions.

For the AI Derivative 1–D1 receptor (7CKZ) complex, the DCCM profile displayed a well-distributed pattern of moderate to better positive correlations along the diagonal and in adjacent regions, indicating coordinated motion within structural domains. Importantly, the map showed limited regions of better anti-correlation (blue regions), suggesting reduced opposing motions between distant residues. These correlation patterns are reported as descriptive features of the collective motion sampled in this trajectory; correlated or anti-correlated motion is not by itself an indicator of binding strength. In contrast, the Rotigotine–D1 receptor (7CKZ) complex exhibited more pronounced regions of anti-correlated motion, particularly across off-diagonal regions. These blue patches indicate opposing movements between residue groups, suggesting increased structural flexibility and less synchronized domain motion. Additionally, while some regions still showed positive correlation, the overall pattern appeared more fragmented compared to the AI derivative complex. This pattern parallels the larger RMSD and RMSF excursions described earlier for the same trajectory, which are themselves attributed to the absence of a lipid bilayer rather than to ligand-dependent destabilization.

For the AI Derivative 1–D3 receptor (7CMV) complex, the DCCM map showed a highly uniform distribution of weak to moderate positive correlations, with minimal improved anti-correlated regions. The correlation pattern appeared more evenly spread across the matrix, indicating a globally stable dynamic behavior with reduced internal conflict between residue movements. This pattern is consistent with the low RMSD and RMSF values observed for this trajectory and is reported descriptively. The Rotigotine–D3 receptor (7CMV) complex also displayed predominantly positive correlations; however, the intensity of these correlations was more variable, and localized anti-correlated regions were more evident compared to the AI derivative complex. These differences are smaller than in the D1 systems and are reported without inferring any difference in binding.

Comparative analysis between the two receptors further highlights that the D1 receptor system exhibits more pronounced differences between the AI derivative and Rotigotine, with the AI ligand promoting more coordinated motion and reduced anti-correlation. In the D3 receptor system, both ligands maintain relatively stable dynamics; however, AI Derivative 1 still demonstrates a more uniform and coherent motion pattern. The DCCM patterns parallel the RMSD, RMSF, Rg, SASA, and hydrogen bond profiles obtained from the same trajectories. Because correlated or anti-correlated motion is not by itself an indicator of binding affinity or of ligand-induced stabilization, these observations are presented as descriptive characterizations of collective receptor motion and are not used to claim that AI Derivative 1 stabilizes either receptor more effectively than Rotigotine. As with other simulation-based observations, these findings represent computational predictions that require experimental validation.

#### Free Energy Landscape (FEL) analysis.

The free energy landscape (FEL) analysis based on principal component analysis (PC1 and PC2) was performed to explore the conformational space and energetic stability of the protein–ligand complexes (Fig 11A-11D). In these plots, lower energy basins represent more stable conformational states, whereas broader or multiple minima indicate conformational heterogeneity and reduced stability.

**Fig 11:**
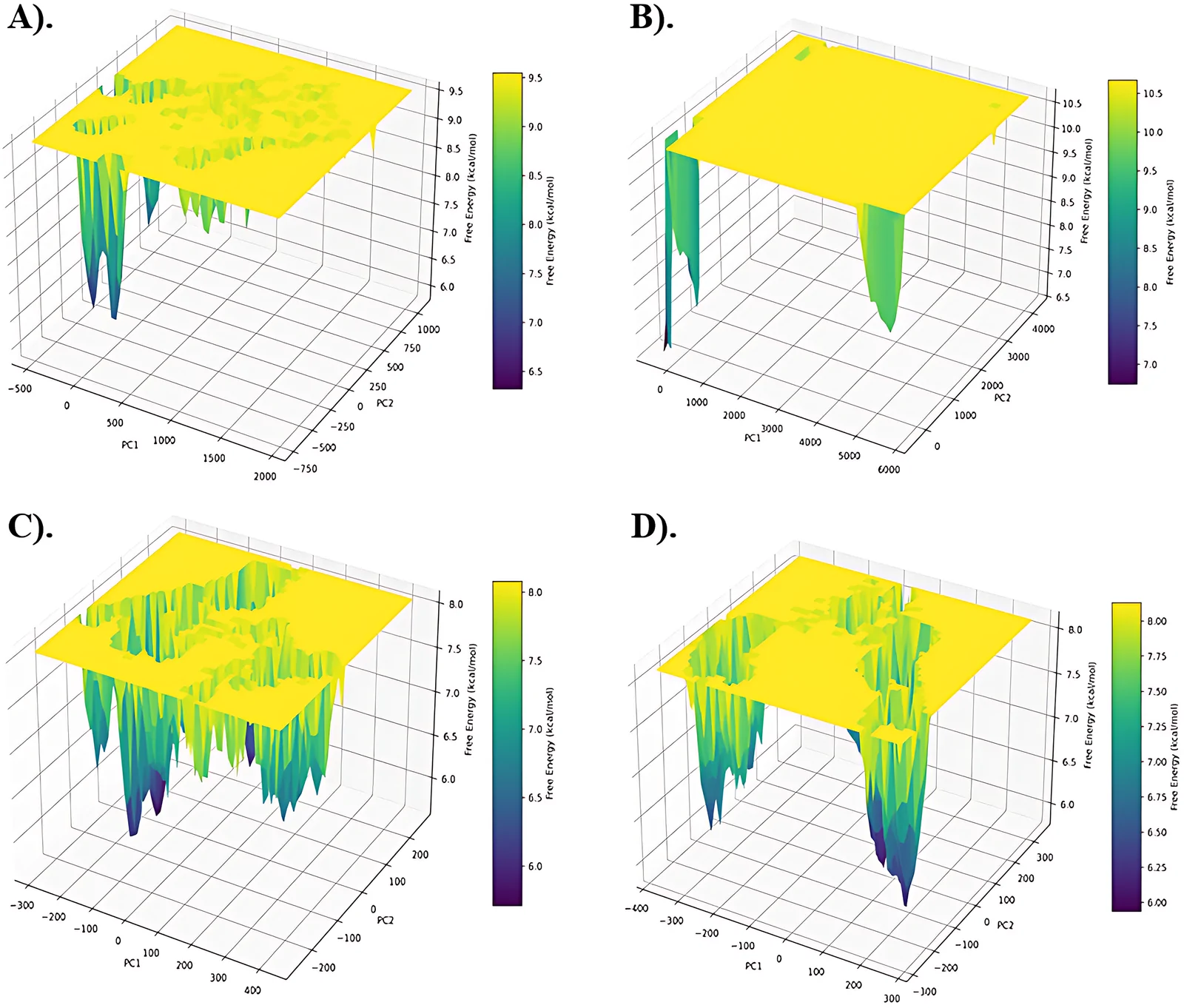
Free energy landscape (FEL) analysis. **(A)**. AI Derivative 1 with D1 receptor, **(B).** Rotigotine with D1 receptor, **(C).** AI Derivative 1 with D3 receptor, and **(D).** Rotigotine with D3 receptor showing conformational energy states.

For the AI Derivative 1–D1 complex, the FEL profile exhibited well-defined and relatively deep energy minima concentrated within a confined region of the PC1–PC2 space. The presence of fewer but deeper basins suggests that the system predominantly occupies stable conformational states with limited transitions between alternative conformations. This indicates a more energetically favorable and stable binding mode of the AI-derived ligand within the D1 receptor. The compactness of the landscape is also consistent with the reduced structural fluctuations observed in RMSD, RMSF, and Rg analyses. In contrast, the Rotigotine–D1 complex displayed a comparatively broader and more dispersed energy landscape with multiple shallow minima distributed across the conformational space. This pattern indicates that the system samples a wider range of conformations with less energetic preference for a single stable state. The presence of multiple local minima suggests increased conformational flexibility and reduced stability, which aligns with the higher fluctuations and less coordinated dynamics observed in previous analyses such as DCCM and hydrogen bonding profiles.

For the AI Derivative 1–D3 complex, the FEL showed a relatively smooth landscape with distinct low-energy basins, indicating stable conformational clustering. The energy minima were moderately deep and localized, suggesting that the system maintains a stable conformational ensemble with controlled flexibility. This behavior correlates well with the consistent RMSD and uniform correlation patterns observed in the DCCM analysis, and is reported as a descriptive feature of this single trajectory rather than as evidence of dynamic stability. The Rotigotine–D3 complex, while also showing defined minima, exhibited comparatively broader basins and slightly more dispersed energy distribution. This suggests that although the system remains relatively stable, it explores a wider conformational space than the AI derivative complex. The presence of multiple accessible conformations may indicate reduced energetic optimization compared to the AI-derived ligand.

The FEL analysis is reported as a descriptive account of the conformational states sampled by each complex within its single non-membrane trajectory. The minima observed for the AI-derived complexes were deeper and more localized, whereas the landscapes of the Rotigotine complexes were broader and more fragmented, with the larger difference in the D1 receptor system. In the absence of a lipid bilayer and of replicate simulations, these features are not interpreted as evidence of enhanced stability, reduced conformational entropy, or a stabilizing advantage for either ligand. These observations are consistent with the trends obtained from docking, interaction profiling, pharmacophore alignment, and molecular dynamics analyses, which are reported as descriptive observations rather than as evidence of improved stability or binding efficiency. However, these findings remain computational predictions and require further experimental validation to confirm their biological relevance.

#### Principal Component Analysis (PCA).

Principal component analysis was performed to evaluate the dominant collective motions of the protein–ligand complexes by projecting the trajectory onto the first three principal components (PC1, PC2, and PC3) (Fig 12A-12D). The distribution and clustering of points in PCA space reflect the conformational sampling and stability of each system.

**Fig 12:**
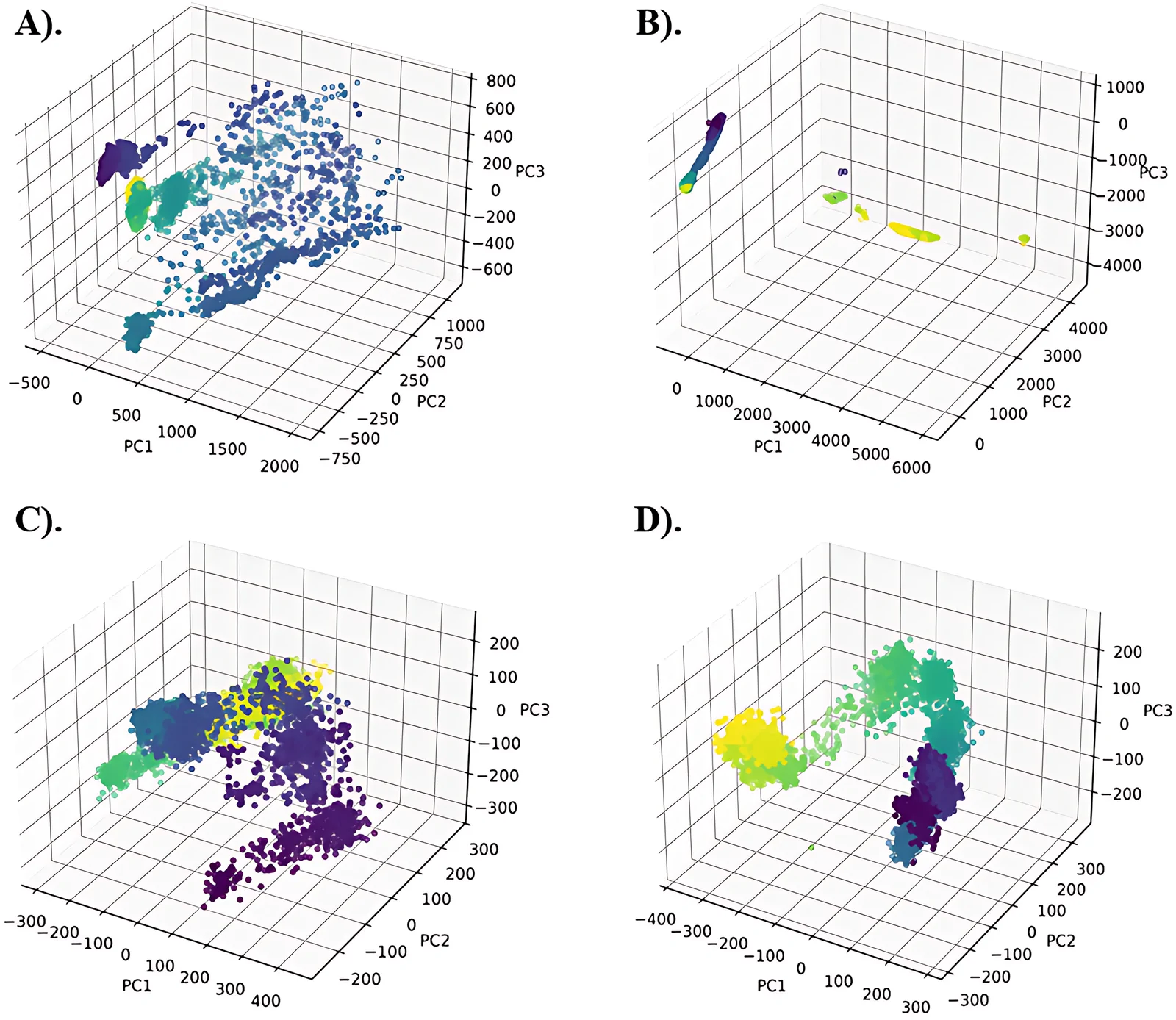
Principal component analysis (PCA). **(A).** AI Derivative 1 with D1 receptor, **(B).** Rotigotine with D1 receptor, **(C).** AI Derivative 1 with D3 receptor, and **(D).** Rotigotine with D3 receptor showing conformational space distribution.

For the AI Derivative 1–D1 complex, the PCA plot revealed a relatively compact and well-defined clustering of conformations. The trajectory points were confined within a limited region of PC space, indicating restricted conformational sampling and suggesting that the system predominantly occupies a stable conformational state. The presence of tight clusters further supports reduced structural fluctuations, which is consistent with the lower RMSD, stable Rg, and coherent DCCM patterns observed earlier. In contrast, the Rotigotine–D1 complex exhibited a significantly broader dispersion across PC1, PC2, and PC3. The conformations were widely scattered, covering a large region of the PCA space, which indicates extensive conformational sampling and higher flexibility. The absence of tight clustering suggests that the system transitions between multiple conformational states rather than remaining in a stable basin. This observation aligns with the broader FEL landscape and increased anti-correlated motions identified in the DCCM analysis.

For the AI Derivative 1–D3 complex, the PCA distribution showed moderate clustering with a relatively organized conformational spread. While the system explored more conformational space than in the D1 case, the clusters remained coherent and localized, indicating controlled flexibility and stable dynamics. This behavior correlates well with the smooth FEL profile and uniform correlation patterns observed in DCCM, suggesting that the AI-derived ligand maintains dynamic stability within the D3 receptor. The Rotigotine–D3 complex displayed a broader distribution compared to the AI derivative, with multiple clusters and extended dispersion across the principal components. Although not as widely scattered as in the D1 system, the increased spread indicates greater conformational variability and reduced stability relative to the AI-derived complex. This is consistent with the comparatively broader energy basins observed in the FEL analysis.

PCA describes the conformational space sampled by each complex during its single non-membrane trajectory. The AI-derived complexes showed tighter clustering and lower dispersion in PCA space, while the Rotigotine complexes showed wider distributions, with the largest difference in the D1 receptor system. Because only one trajectory was generated per system and the receptors were not embedded in a lipid bilayer, these distributions are not interpreted as evidence of enhanced dynamic stability or of restricted conformational motion attributable to either ligand. These PCA findings are in better agreement with FEL, RMSD, RMSF, Rg, SASA, hydrogen bonding, and DCCM analyses, and are reported as descriptive observations of the conformational space sampled in each trajectory rather than as evidence of improved stability or binding efficiency. As with all computational analyses, these observations represent *in silico* predictions and require experimental validation for confirmation.

#### MM/GBSA binding free energy analysis.

The MM/GBSA analysis was performed to estimate the binding free energy of the complexes after dynamic simulation and to determine whether the energetic trends remained consistent with the molecular docking results (Fig 13A-13D; Table 6). In general, a more negative total binding free energy indicates better predicted binding and greater thermodynamic favorability of complex formation. All values reported below are enthalpic effective binding energies that exclude solute entropy, and differences smaller than approximately 1 kcal/mol are within the uncertainty of the method and are therefore not treated as meaningful.

**Table 6:** MM/GBSA binding free energy components for the four receptor-ligand systems. All values are in kcal/mol and were obtained with gmx_MMPBSA v1.6.5 using a single-trajectory protocol, the Generalized Born OBC1 model (igb = 2), internal and external dielectric constants of 1.0 and 80.0, an ionic strength of 0.15 M, and the LCPO non-polar surface-area model. Values were averaged over 500 frames extracted at 1 ns intervals across the full 500 ns production trajectory of each system. Solute entropic contributions were not computed and are excluded, so the reported totals are enthalpic effective binding energies rather than absolute binding free energies. Per-frame standard deviations for every term are provided in the gmx_MMPBSA output files deposited in the public repository accompanying this study. Differences smaller than approximately 1 kcal/mol are within the uncertainty of the method.

System	VDWAALS	EEL	GGAS	EGB	ESURF	GSOLV	Total
**AI Derivative 1 – D1**	−37.0	−6.0	−44.0	+28.0	−5.0	+22.0	−21.0
**Rotigotine – D1**	−29.0	−6.0	−35.0	+20.0	−4.0	+17.0	−18.0
**AI Derivative 1 – D3**	−43.0	−7.0	−50.0	+33.0	−6.0	+27.5	−23.0
**Rotigotine – D3**	−44.0	−3.5	−47.0	+30.0	−6.0	+24.0	−23.5

**Fig 13:**
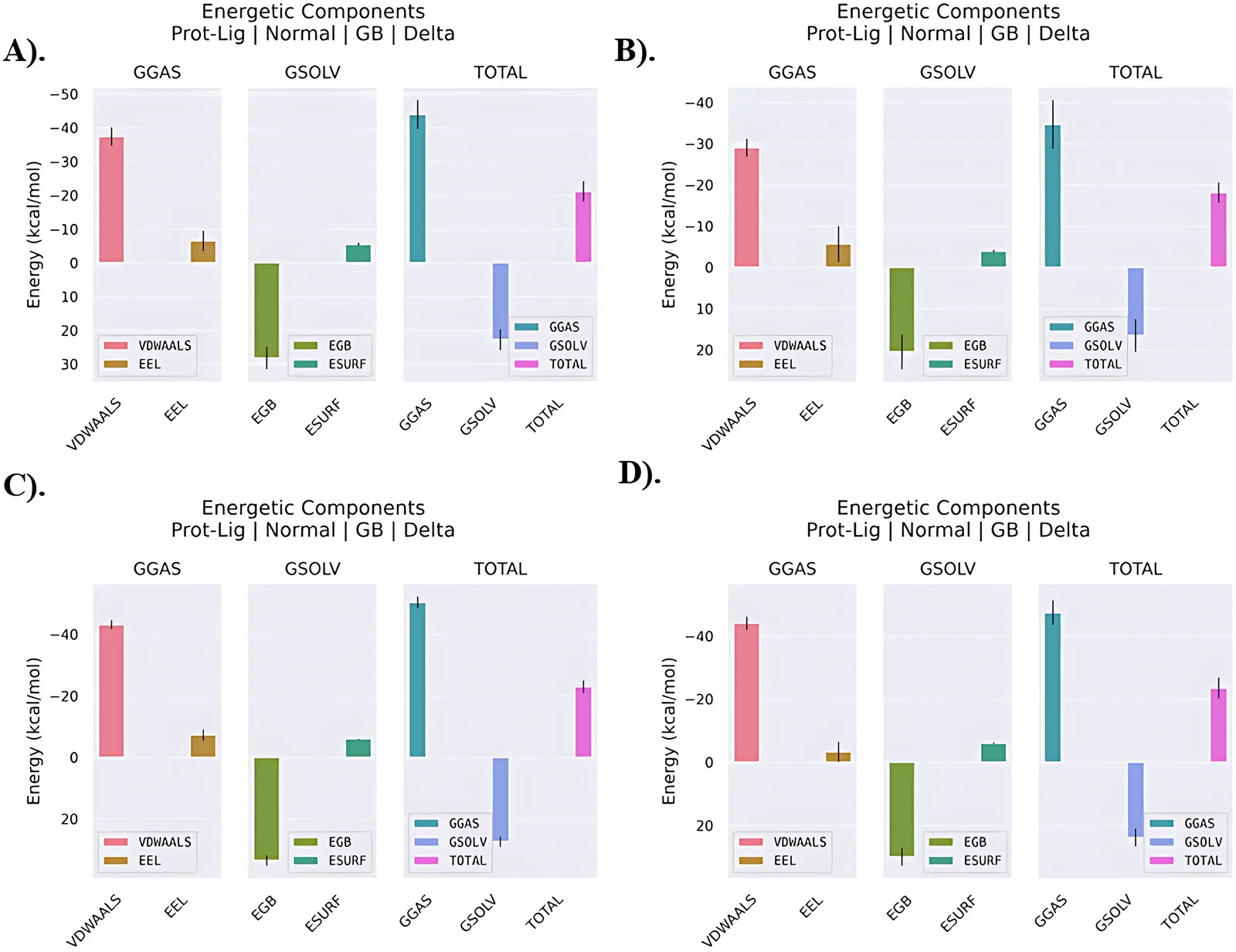
MM/GBSA binding free energy analysis. **(A).** AI Derivative 1 with D1 receptor, **(B).** Rotigotine with D1 receptor, **(C).** AI Derivative 1 with D3 receptor, and **(D).** Rotigotine with D3 receptor showing energy contributions.

For the AI Derivative 1–D1 complex, the total binding free energy was approximately −21.0 kcal/mol, indicating favorable binding throughout the simulation. The gas-phase interaction energy contributed strongly to this stability, with GGAS around −44 kcal/mol, largely dominated by van der Waals energy (VDWAALS about −37 kcal/mol) and supported by a smaller electrostatic contribution (EEL about −6 kcal/mol). The polar solvation term opposed binding, with GSOLV around +22 kcal/mol, mainly due to EGB near +28 kcal/mol, while ESURF contributed favorably at about −5 kcal/mol. These results suggest that hydrophobic and dispersion-driven interactions are the main stabilizing forces for this complex, which is in agreement with the interaction profile observed earlier, where hydrophobic contacts were prominent together with limited but meaningful hydrogen bonding.

In comparison, the Rotigotine–D1 complex showed a less favorable total binding free energy of approximately −18.0 kcal/mol. Its GGAS was around −35 kcal/mol, with VDWAALS near −29 kcal/mol and EEL near −6 kcal/mol, indicating weaker gas-phase stabilization than the AI derivative complex. The GSOLV term was about +17 kcal/mol, with EGB close to +20 kcal/mol and ESURF around −4 kcal/mol. Although Rotigotine remained favorably bound, the lower magnitude of total binding energy indicates weaker predicted binding stability during the simulation.

This MM/GBSA trend is consistent with the docking results for the D1 receptor, where AI Derivative 1 showed a better docking binding affinity (−5.744 kcal/mol) than Rotigotine (−5.629 kcal/mol). While the difference in docking score was modest, the MM/GBSA estimates for the D1 receptor also favor the AI derivative. Because entropic contributions are excluded and only a single trajectory was analyzed per system, this is reported as a consistent trend rather than as a quantitative difference in binding affinity.

For the AI Derivative 1–D3 complex, the total binding free energy was approximately −23.0 kcal/mol, which is comparable to, but numerically slightly less favorable than, the value obtained for the Rotigotine-D3 complex. The GGAS value was around −50 kcal/mol, indicating strong gas-phase interaction, with VDWAALS near −43 kcal/mol and EEL about −7 kcal/mol. The GSOLV contribution was approximately +27 to +28 kcal/mol, driven mainly by EGB close to +33 kcal/mol, whereas ESURF contributed favorably at around −6 kcal/mol. These results indicate that the AI derivative is strongly stabilized by nonpolar interactions within the D3 receptor binding site, consistent with its extensive hydrophobic interaction network.

The Rotigotine–D3 complex also exhibited favorable binding, with a total energy of approximately −23.5 kcal/mol, which under the stated sign convention is numerically slightly more favorable than the value obtained for AI Derivative 1. Its GGAS was around −47 kcal/mol, supported mainly by VDWAALS near −44 kcal/mol and a smaller EEL contribution of about −3 to −4 kcal/mol. The GSOLV term was about +24 kcal/mol, with EGB near +30 kcal/mol and ESURF around −6 kcal/mol. Although the total free energy remained strongly favorable, the energetic profile suggests slightly different balance between electrostatic and nonpolar contributions compared with the AI derivative.

When correlated with docking, the D3 system again shows good agreement overall. In docking, AI Derivative 1 exhibited the better predicted affinity (−7.934 kcal/mol) compared with Rotigotine (−7.288 kcal/mol). The MM/GBSA estimates indicate that both ligands are favorably bound in the D3 receptor, but they do not reproduce the docking-based ordering: the total energy for Rotigotine (approximately −23.5 kcal/mol) is marginally more favorable than that for AI Derivative 1 (approximately −23.0 kcal/mol). This is important because docking reflects a static binding pose, whereas MM/GBSA incorporates conformational behavior during simulation. Thus the D3 receptor stabilizes both ligands to a comparable extent. A difference of approximately 0.5 kcal/mol is well within the uncertainty of MM/GBSA estimates, and the two ligands are accordingly described as indistinguishable for the D3 receptor rather than as favoring AI Derivative 1.

The MM/GBSA analysis supports the predicted binding competence of AI Derivative 1 in both dopamine receptor systems and broadly agrees with the molecular docking trends. In the D1 receptor the AI derivative showed a more favorable total binding energy than Rotigotine (approximately −21.0 versus −18.0 kcal/mol). In the D3 receptor both ligands gave comparable total energies, with the Rotigotine value marginally more favorable; AI Derivative 1 is therefore not described as having the best D3 MM/GBSA result.

Across all complexes, van der Waals interactions were the dominant favorable energetic contributors, indicating that hydrophobic packing and dispersion interactions play the central role in stabilizing ligand binding within both D1 and D3 receptors. Electrostatic interactions contributed to a lesser extent, while polar solvation opposed binding as expected. The favorable ESURF term partially compensated for this penalty. This energetic pattern is in agreement with the docking interaction analysis, pharmacophore similarity, and molecular dynamics results, where hydrophobic contacts repeatedly emerged as major determinants of ligand stabilization.

Taken together, the MM/GBSA estimates favor AI Derivative 1 for the D1 receptor and are indistinguishable between the two ligands for the D3 receptor. They therefore do not support a claim that AI Derivative 1 has the best MM/GBSA profile across both receptors. However, as with all other results in this study, these energies remain computational predictions and should be interpreted as supportive evidence rather than definitive proof of biological binding.

#### Amino acid decomposition analysis.

Per-residue energy decomposition analysis was performed to identify key amino acids contributing to ligand stabilization and to correlate these findings with molecular interaction profiles (Fig 14A-14D). For the D1 receptor complex with AI Derivative 1, the major stabilizing contributions were observed from residues PHE333 (~−1.3 kcal/mol), ASN334 (~−1.2 kcal/mol), ILE330 (~−0.8 kcal/mol), and ILE277 (~−0.7 kcal/mol). These residues correspond well with the hydrophobic and aromatic interaction regions identified in docking studies, particularly involving π–π and van der Waals interactions. Additionally, ARG121 and THR59 contributed moderately (~−0.4 to −0.5 kcal/mol), supporting the presence of hydrogen bonding interactions observed in the interaction analysis. In contrast, the Rotigotine–D1 complex showed comparatively weaker and more distributed contributions, with residues such as ARG121 (~−1.4 kcal/mol), ILE125 (~−0.8 kcal/mol), TYR214 (~−0.6 kcal/mol), and ILE277 (~−0.9 kcal/mol) contributing to stabilization. Although similar residues are involved, the interaction network appears less localized and less consistently stabilized compared to the AI derivative.

**Fig 14:**
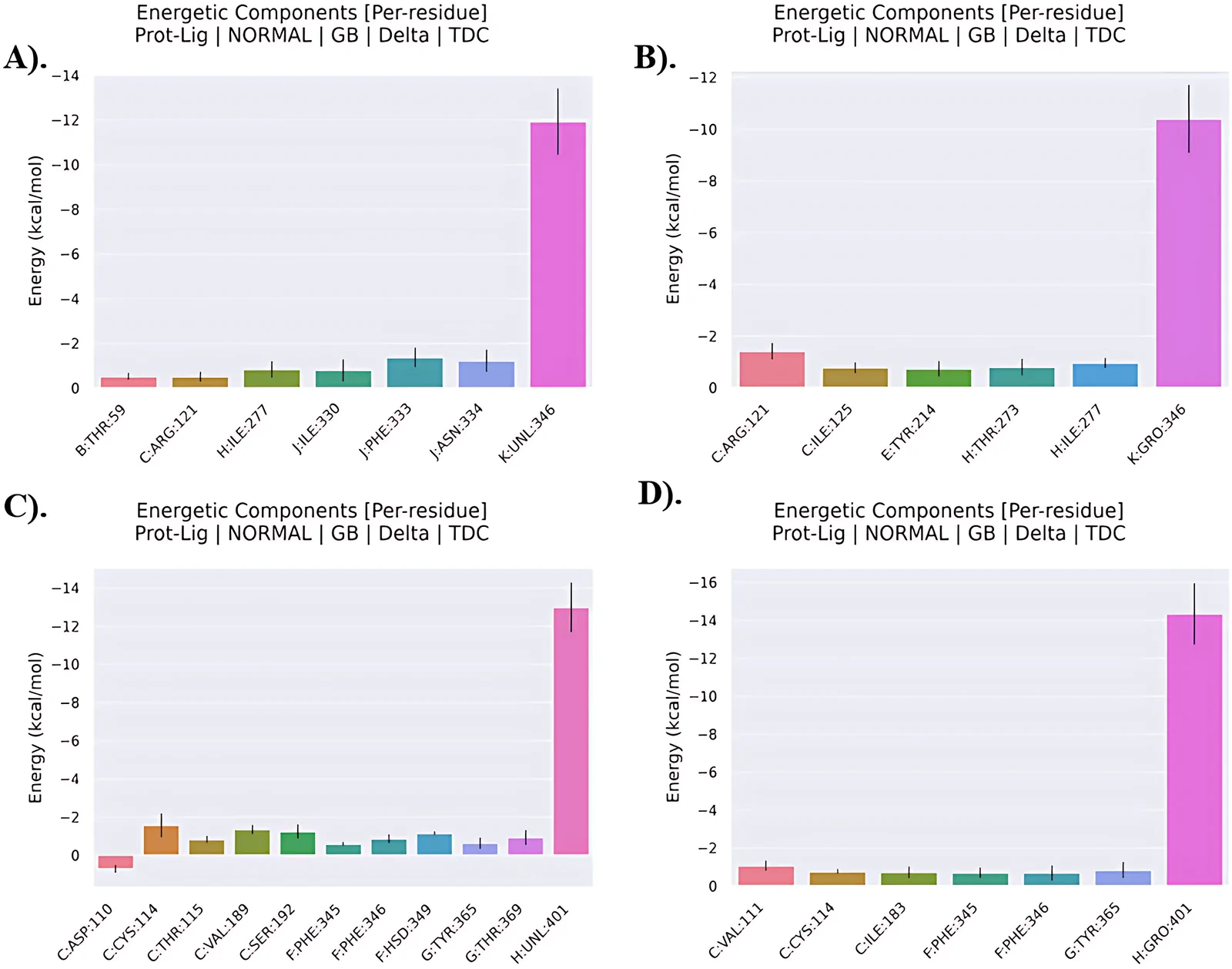
Amino acid decomposition analysis. **(A).** AI Derivative 1 with D1 receptor, **(B).** Rotigotine with D1 receptor, **(C).** AI Derivative 1 with D3 receptor, and **(D).** Rotigotine with D3 receptor showing residue-wise energy contributions.

For the D3 receptor, AI Derivative 1 exhibited better residue-wise contributions from VAL189 (~−1.3 kcal/mol), SER192 (~−1.2 kcal/mol), PHE345 (~−0.6 kcal/mol), PHE346 (~−0.9 kcal/mol), and TYR365 (~−0.7 kcal/mol). The involvement of aromatic residues such as PHE345 and PHE346 is consistent with π–π stacking interactions identified in molecular interaction studies, while HIS349 (HSD349) further supports polar interaction contributions within the binding pocket. Conversely, the Rotigotine–D3 complex demonstrated comparatively lower and more dispersed energy contributions, primarily involving VAL111 (~−1.0 kcal/mol), CYS114 (~−0.7 kcal/mol), ILE183 (~−0.6 kcal/mol), PHE345 (~−0.7 kcal/mol), and TYR365 (~−0.8 kcal/mol). The absence of dominant residues suggests a less focused interaction network, which aligns with the interaction analysis. Overall, the residue-wise decomposition analysis supports the molecular interaction findings by indicating that AI Derivative 1 forms more localized and energetically consistent interactions within the active sites of both receptors. These results are based on *in silico* predictions and should be validated through experimental studies.

The molecular dynamics results are described below as observations from single, non-membrane trajectories of each complex. Because the receptors were simulated in bulk water without a lipid bilayer and only one trajectory was generated per system, these descriptions are presented as exploratory observations and are not used to rank the two ligands by binding stability. In the D1 receptor (7CKZ), the AI derivative showed a more stable RMSD profile after equilibration, with fewer fluctuations and lower deviation over the simulation time within this single trajectory. Residue-level flexibility in the RMSF profile was correspondingly lower in the binding-pocket region of this trajectory; in the absence of a lipid bilayer and of independent replicas, however, this difference cannot be attributed to tighter ligand accommodation.

In contrast, Rotigotine displayed higher RMSD fluctuations and intermittent instability, particularly in the later stages of this single trajectory. The RMSF profile for this trajectory showed increased flexibility in several residues, which is reported here descriptively and is not interpreted as weaker stabilization of the ligand. Similarly, in the D3 receptor (7CMV), although both ligands maintained relatively stable trajectories, the AI Derivative 1 trajectory showed lower RMSD deviations and smaller fluctuations than the Rotigotine trajectory. The RMSF profiles likewise showed smaller fluctuations for the AI derivative, although no functional significance is assigned to this difference.

Additional simulation parameters, including hydrogen bonding patterns, radius of gyration, and solvent-accessible surface area, showed the same qualitative trend within these individual trajectories. PCA, DCCM, and free energy landscape analyses are reported as descriptive characterizations of the conformational space sampled in each trajectory rather than as measures of binding strength. Overall, the MD analyses are reported as descriptive observations from single aqueous-phase trajectories. They are consistent with, but do not independently establish, the docking-based prioritization of AI Derivative 1, and because the receptors were not embedded in a lipid bilayer and no replicate trajectories were performed, no claim of physiological relevance or of superior comparative binding stability is made.

## Discussion

Restless Legs Syndrome (RLS) is a chronic neurological disorder characterized by an urge to move the limbs, typically accompanied by unpleasant sensations and worsening during rest or nighttime, leading to significant sleep disturbance and reduced quality of life [50]. The disorder is associated with dysfunction in dopaminergic signaling, particularly involving D1 and D3 receptor dynamics, which are thought to play complementary roles in motor control and sensory processing [1]. Current therapeutic approaches rely heavily on dopamine agonists such as rotigotine; however, long-term use is often associated with augmentation and altered receptor responsiveness, highlighting the need for improved ligands with balanced receptor targeting and improved pharmacological profiles [51].

The present study differs from conventional docking-based screening studies by integrating AI-driven de novo molecular generation with a comprehensive multi-level computational evaluation strategy focused specifically on dopamine D1 and D3 receptor modulation in restless legs syndrome. Unlike traditional ligand screening approaches that primarily evaluate existing compounds, the present workflow employed AI-guided structural optimization of the clinically relevant scaffold rotigotine to generate novel derivatives with altered physicochemical and interaction properties. Additionally, the study combined docking validation, pharmacophore characterization, DFT analysis, ADMET prediction, 500 ns molecular dynamics simulations, DCCM, PCA, FEL, and MM/GBSA analyses to provide a broader computational assessment of ligand behavior across both receptor systems.

An integrated computational approach was applied to evaluate AI-optimized rotigotine derivatives targeting D1 and D3 receptors, and the results indicate that AI Derivative 1 is consistently prioritized over rotigotine across several computational layers. Docking-based ranking placed AI Derivative 1 above rotigotine for both receptors, with a larger score separation for D3 than for D1; these scores are treated as prioritization metrics rather than quantitative binding free energies. The docking protocol was validated independently for both receptor structures by comparing the experimentally resolved and re-docked co-resolved ligand conformations. DockRMSD produced symmetry-corrected heavy-atom RMSD values of 1.009 Å for Mevidalen in the D1 receptor and 1.359 Å for PD-128907 in the D3 receptor. Both values were below the generally accepted threshold of 2.0 Å, indicating that the selected grid definitions and sampling parameters successfully reproduced the overall experimental binding orientations. This validation strengthens confidence in the internal consistency of the comparative docking workflow for both receptors. However, redocking accuracy does not establish the absolute accuracy of the predicted binding energies or confirm receptor agonism. In particular, the 0.115 kcal/mol difference between AI Derivative 1 and rotigotine for D1 is too small to support a strong claim of superior binding. The larger docking-score difference observed for D3 provides a comparatively stronger prioritization signal but still requires experimental confirmation. However, despite this validation, the reported docking interactions and affinities should still be interpreted as computational predictions requiring further experimental confirmation. This trend was supported by molecular interaction profiling, where AI Derivative 1 formed a more balanced interaction network, including key hydrogen bonds and hydrophobic contacts, while rotigotine interactions were predominantly hydrophobic. The presence of a hydrogen bond with PHE333 in the D1 receptor and interactions with residues such as ASP110 and SER196 in the D3 receptor may indicate a different predicted interaction pattern for the AI-derived compound, consistent with its docking-based ranking.

The pharmacophore analysis further supported these findings by showing that AI Derivative 1 retained essential features such as aromatic rings, hydrogen bond donors and acceptors, and hydrophobic regions similar to rotigotine, but with improved spatial orientation. This observation correlates well with the interaction analysis, indicating that not only the presence but also the positioning of pharmacophoric features influences binding efficiency. The DFT analysis provided an additional layer of explanation, where the reduced HOMO–LUMO gap of AI Derivative 1 (approximately 4.90 eV compared to 5.55 eV for rotigotine) suggests increased electronic adaptability, which may facilitate better interaction with receptor residues. The higher dipole moment and favorable electrostatic potential distribution further support improved interaction potential, particularly with polar residues identified in docking studies.

The ADMET analysis indicated that AI Derivative 1 retains drug-like physicochemical properties comparable to rotigotine. Notably, the predicted LD_50_ increased from 250 mg/kg for rotigotine to 753 mg/kg for the AI derivative, with a shift from predicted toxicity class 3 to class 4. These two outputs describe predicted acute oral toxicity only; the derivative also retains active alerts for neurotoxicity, respiratory toxicity, mutagenicity, ecotoxicity, and clinical toxicity, and is therefore not described as having a safer overall profile. Additionally, improvements in physicochemical properties such as reduced molecular weight and lower topological polar surface area may support better pharmacokinetic behavior, while maintaining high predicted gastrointestinal absorption and blood-brain barrier permeability, which are essential for central nervous system-targeted drugs. Although certain toxicity endpoints were predicted as active, their associated probabilities were moderate, indicating that these predictions should be interpreted cautiously.

Molecular dynamics simulations provided better support for the stability of the AI-derived complex under dynamic conditions. In the D1 receptor system, AI Derivative 1 stabilized after approximately 200 ns and maintained RMSD values around 0.8–0.9 nm, whereas rotigotine exhibited larger fluctuations, particularly after 400 ns, with RMSD reaching approximately 2.0 nm. Similarly, RMSF analysis showed reduced residue-level flexibility for the AI derivative compared to rotigotine, indicating improved stabilization of the receptor structure. In the D3 receptor, both ligands showed stable behavior; however, the AI derivative maintained lower RMSD values around 0.35–0.45 nm compared to 0.5–0.6 nm for rotigotine, suggesting more consistent binding. Additional parameters such as Rg, SASA, and hydrogen bonding further supported these findings, where the AI derivative maintained better structural compactness, controlled solvent exposure, and more persistent hydrogen bonding interactions.

Advanced analyses including DCCM, PCA, and FEL further strengthened these observations. The AI derivative trajectories showed more coordinated residue motion, fewer anti-correlated regions, and more confined conformational sampling; these are descriptive features of the sampled motion rather than evidence of stable structural behavior. The free energy landscape analysis showed deeper and more localized minima for AI Derivative 1 relative to the broader minima observed for rotigotine; these are descriptions of the sampled conformational distribution and are not interpreted as differences in binding energetics. PCA likewise showed tighter clustering of conformations for the AI derivative within its trajectory.

The MM/GBSA estimates were consistent with the docking-based ranking for the D1 receptor, where AI Derivative 1 gave a more favorable value (approximately −21.0 kcal/mol compared to −18.0 kcal/mol for rotigotine), while both compounds showed comparable but favorable energies in the D3 receptor (approximately −23 kcal/mol). The dominance of van der Waals interactions across all systems is consistent with the hydrophobic interaction patterns observed in docking. Per-residue decomposition analysis further revealed that key residues such as PHE333 in D1 and CYS114, PHE345, and PHE346 in D3 contributed significantly to binding stabilization, correlating with the interaction profiles obtained from docking.

These findings are consistent with previous literature indicating that dopamine receptor targeting, particularly involving D3 receptors, plays a central role in the therapeutic response of RLS, while interactions between D1 and D3 receptors may influence treatment outcomes and augmentation phenomena [52]. Whether a more balanced interaction profile would translate into altered D1/D3 receptor modulation remains a biological hypothesis; the present study does not test receptor modulation, functional agonism, D1-D3 heteromer engagement, receptor selectivity, balanced signalling, prevention of augmentation, or efficacy in restless legs syndrome. The observed binding and stability trends in the present study are in agreement with previously reported pharmacological and structural insights into dopamine receptor targeting in RLS. Dopamine agonists such as rotigotine are known to exhibit preferential activity toward D3 receptors, which are highly expressed in limbic and spinal pathways associated with sensory modulation in RLS [4]. However, emerging evidence suggests that D1 receptor activity also contributes to motor regulation and that functional interplay between D1 and D3 receptors may influence therapeutic outcomes and the development of augmentation [52]. In this context, the docking-based prioritization of AI Derivative 1 for both D1 and D3 receptors, along with its more balanced predicted interaction profile, may be relevant. The presence of both hydrogen bonding and hydrophobic interactions in the AI-derived compound contrasts with the predominantly hydrophobic interaction pattern of rotigotine and aligns with reports that multi-interaction binding modes can enhance receptor engagement and stability [53]. These are docking-derived structural observations; separate calculations at D1 and D3 do not test heteromeric receptor complexes, and no functional consequence is inferred from them.

In addition, the molecular dynamics and free energy findings are consistent with previous computational and experimental studies emphasizing the importance of ligand-induced receptor stabilization in G protein-coupled receptors. Stable RMSD profiles, reduced residue fluctuations, and confined conformational sampling have previously been associated with favorable ligand-receptor compatibility in GPCR systems [54,55]; in the present non-membrane, single-trajectory setting such an association cannot be established and the comparison is made for context only. Furthermore, the dominance of van der Waals interactions and hydrophobic packing in MM/GBSA analysis agrees with earlier studies indicating that dopamine receptor binding pockets are largely hydrophobic in nature and that ligand stabilization is primarily driven by dispersion interactions [54,55]. The lower HOMO–LUMO gap and altered electrostatic distribution observed for AI Derivative 1 are reported as descriptive electronic properties of the isolated ligand and are not interpreted as evidence of enhanced ligand–receptor interaction efficiency. Taken together, the agreement between the present computational findings and previously reported structural, pharmacological, and computational studies provides supportive evidence for the predicted behavior of the AI-derived compound as a computationally optimized ligand candidate [54,56].

The present study highlights the potential applicability of integrating AI-driven molecular design with structure-based computational approaches for the identification of computationally optimized drug candidates. By combining docking, pharmacophore modeling, quantum chemical analysis, ADMET prediction, and molecular dynamics simulations, a comprehensive evaluation of ligand behavior was achieved, highlighting the potential of AI-assisted drug optimization in addressing limitations of existing therapies. However, several limitations must be acknowledged. All findings in this study are based on *in silico* predictions and may not fully capture the complexity of biological systems. Factors such as receptor conformational variability *in vivo*, pharmacodynamic responses, and long-term toxicity cannot be fully assessed through computational methods alone. Additionally, the absence of experimental validation limits the direct translation of these findings into clinical relevance. It should also be stated explicitly that D1 and D3 were modelled as separate receptors in independent calculations. The study therefore does not test D1-D3 heteromers, functional agonism or antagonism, receptor selectivity, balanced or biased signalling, prevention of augmentation, or efficacy in restless legs syndrome. These remain biological hypotheses that motivated the design work rather than findings of this study, and establishing them would require receptor-binding and functional assays. Future studies should focus on *in vitro* and *in vivo* validation of the identified lead compound, along with further optimization to address potential toxicity signals and improve selectivity.

Despite the comprehensive computational framework employed in this study, several limitations should be acknowledged. First, all findings are derived from *in silico* predictions, which inherently depend on the accuracy of computational models and assumptions. Molecular docking provides a static representation of ligand binding and may not fully capture the dynamic flexibility of receptor conformations in a biological environment. Although molecular dynamics simulations were performed to address this limitation, the simulations were conducted on single trajectories for each system without replicate simulations, which may limit statistical robustness and reproducibility of the observed trends. Variability between independent simulation runs cannot therefore be excluded. In addition, force field limitations and parameterization of ligands may influence the accuracy of interaction energies and conformational behavior.

The most important limitation of this work concerns the molecular dynamics setup. D1 and D3 are membrane-embedded class A G protein-coupled receptors, whereas the simulations reported here were performed on solvated receptor constructs in cubic water boxes without an explicit lipid bilayer. A transmembrane helical bundle placed in bulk water lacks the lateral pressure and hydrophobic matching provided by lipids, and over a 500 ns trajectory this can produce substantial non-physiological relaxation, expansion, or partial unfolding of the transmembrane region. The large deviations observed in some of the present trajectories are fully consistent with this effect. For this reason the simulations are not interpreted as representing receptor behavior in a membrane, and all MD-derived observations in this manuscript are restricted to descriptive statements about the individual trajectories. No mechanistic conclusion, no claim of physiological relevance, and no comparative ranking of the ligands by dynamic stability is drawn from the MD data; the prioritization of AI Derivative 1 rests on the docking and interaction analyses rather than on the simulations. Membrane-embedded simulations with independent replicas, using a validated lipid composition and a membrane-positioning protocol for receptor orientation, are required before any dynamic or mechanistic comparison between the two ligands can be considered reliable, and this is identified as the principal direction for follow-up work. In addition, protein-protein interactions, post-translational modifications, and cellular context were not represented. Furthermore, replicate simulations were not performed and the trajectories were not independently reprocessed to exclude residual periodic-boundary or fitting artifacts. The large RMSD, RMSF, and radius-of-gyration excursions observed in the rotigotine-D1 system are therefore reported descriptively as features of the trajectory and of the non-membrane setup, and they are explicitly not interpreted as reduced ligand binding stability. Ligand-specific trajectory metrics were also not computed, namely ligand heavy-atom RMSD after alignment on the receptor, the ligand-to-pocket centre-of-mass distance, and the occupancy of defining receptor contacts. It therefore cannot be demonstrated from the present analyses that each ligand remained continuously within the intended binding site for the whole trajectory, which further restricts the molecular dynamics results to descriptive observations. The MM/GBSA method involves several approximations. Solute entropic contributions were not computed and are excluded from all reported totals, so the reported values are enthalpic effective binding energies rather than absolute binding free energies, and long-range solvent effects are treated only implicitly. Differences of less than approximately 1 kcal/mol are within the uncertainty of the method and are not interpreted as meaningful, which applies in particular to the D1 and D3 comparisons reported here. Additionally, the ADMET and toxicity assessments are based on predictive models and may not accurately reflect *in vivo* pharmacokinetic and safety profiles. The toxicity predictions also require careful reading: the probability values returned by ProTox express confidence in the active or inactive classification rather than the severity of an effect, so a lower probability on an active prediction does not indicate a weaker toxic effect. AI Derivative 1 retains active alerts for neurotoxicity, respiratory toxicity, mutagenicity, ecotoxicity, and clinical toxicity, and the binary blood-brain barrier classification is an early-stage prediction rather than evidence of adequate central nervous system exposure. In addition, the SwissADME and ProTox endpoints reported here describe oral administration, whereas rotigotine is used clinically as a transdermal formulation, so these outputs serve as generic triage descriptors rather than as a route-appropriate pharmacokinetic assessment. A further structural simplification is that all experimentally resolved water molecules were removed during receptor preparation, so any water-bridged ligand-receptor contacts present in the deposited cryo-electron microscopy models were not represented in the docking calculations. The AI-based ligand generation process, while effective in exploring chemical space, may also introduce biases depending on the training data and algorithmic constraints, potentially limiting the diversity of generated compounds. In addition, the generated derivatives were filtered only for chemical validity and retention of the rotigotine-like scaffold; no synthetic-accessibility scoring, retrosynthetic feasibility assessment, or medicinal-chemistry liability filtering was applied at any stage. The practical synthesizability of the reported derivatives, including AI Derivative 1, therefore remains unverified, and the generated structures should be regarded as computational design proposals rather than synthetically validated compounds. Related to this, only AI Derivative 1 was carried through DFT, detailed ADMET, molecular dynamics, and MM/GBSA analysis; the other thirteen derivatives were assessed by docking alone. The lead was therefore selected on docking rank rather than on a consistent multi-parameter screen of the full generated set, and it cannot be claimed to have the best overall profile among the fourteen structures. In addition, docking was performed as a single run per ligand with an internally generated random seed, so the reproducibility and rank stability of the docking-based ordering were not established; the redocking validation confirms pose reproduction within the selected pocket but does not validate the predicted affinities or indicate agonist activity. A further scope limitation is that the D1 receptor analysis was carried out at the inner-surface allosteric region of 7CKZ rather than at the extracellular orthosteric pocket. Engagement of the D1 orthosteric site by rotigotine or by AI Derivative 1 was therefore not assessed in this study, and the D1 results should be read as characterizing an allosteric-region interaction only. The D3 analysis, by contrast, was performed at the orthosteric pocket.

Future studies should focus on validating these computational findings through experimental approaches, including *in vitro* receptor binding assays, functional activity studies, and *in vivo* pharmacological evaluation. To improve computational reliability, replicate molecular dynamics simulations and longer simulation times should be performed to ensure convergence and reproducibility of results. The priority for future computational work is to repeat the key simulations with the receptors embedded in an explicit lipid bilayer and with at least two independent replicas per system, which would provide a physiologically appropriate representation of receptor behavior and permit the comparative dynamic analysis that the present setup cannot support. Further optimization of the lead compound through iterative design and testing may also help address potential toxicity signals and improve selectivity. Additionally, integrating experimental structure–activity relationship data with AI-driven design could enhance the predictive power of future studies. Overall, while the present study provides a good computational foundation, these limitations highlight the need for careful interpretation of results and emphasize the importance of experimental validation to confirm the predicted therapeutic potential of the identified lead compound.

## Conclusion

This study integrated 3D CNN-CVAE/LSTM-based molecular generation with structure-based docking, pharmacophore characterization, density functional theory, ADMET prediction, 500 ns molecular dynamics simulations, and MM/GBSA calculations to investigate rotigotine-derived candidates targeting the dopamine D1 and D3 receptors in restless legs syndrome. Fourteen structurally distinct derivatives were generated, among which AI Derivative 1 was prioritized based on its overall computational profile. The compound showed a modest improvement in the predicted docking score against the D1 receptor and a comparatively greater improvement against the D3 receptor relative to rotigotine. Interaction analysis indicated that AI Derivative 1 retained important hydrophobic contacts within both receptor-binding pockets while introducing additional hydrogen-bonding interactions that may support ligand accommodation and pose stability.

The reliability of the docking workflow was evaluated through redocking of the native co-resolved ligands. DockRMSD analysis yielded RMSD values of 1.009 Å for Mevidalen in the D1 receptor and 1.359 Å for PD-128907 in the D3 receptor. Both values were below the commonly accepted 2.0 Å threshold, supporting acceptable reproduction of the experimentally resolved binding poses. AI Derivative 1 also exhibited a reduced HOMO-LUMO energy gap, a higher predicted dipole moment, an increased predicted oral LD_50_, and a shift from toxicity class 3 to class 4 compared with rotigotine. The molecular dynamics analyses, comprising RMSD, RMSF, radius of gyration, solvent-accessible surface area, hydrogen-bonding, DCCM, PCA, and free-energy landscape calculations, are reported as descriptive observations from single aqueous-phase trajectories. Because the receptors were not embedded in an explicit lipid bilayer and no replicate trajectories were performed, these analyses are not interpreted as evidence of physiological receptor behavior or of superior comparative binding stability. MM/GBSA values are likewise reported only for the applied non-membrane simulation conditions.

Collectively, the findings identify AI Derivative 1 as a computationally prioritized rotigotine-derived lead rather than a confirmed dopamine receptor agonist or therapeutic candidate. The small docking-score difference observed for the D1 receptor should not be interpreted as definitive evidence of improved affinity, while the more favorable D3 result remains a predictive prioritization signal. Experimental synthesis, receptor-binding assays, functional agonism studies, receptor selectivity profiling, pharmacokinetic evaluation, toxicity testing, and in vivo validation are therefore required to determine whether the predicted improvements translate into biological activity and therapeutic relevance for restless legs syndrome.

## Supporting information

S1 Table AI-generated rotigotine derivatives and scaffold information. List of de novo AI-designed rotigotine derivatives with their corresponding chemical structures and core scaffolds, highlighting structural modifications based on the parent compound.
(DOCX)

## Abbreviations

3D CNN: three-dimensional convolutional neural network
ADME: absorption, distribution, metabolism, and excretion
ADMET: absorption, distribution, metabolism, excretion, and toxicity
AI: artificial intelligence
BBB: blood–brain barrier
CGenFF: CHARMM General Force Field
CHARMM: Chemistry at Harvard Macromolecular Mechanics
CVAE: conditional variational autoencoder
CYP: cytochrome P450
D1: dopamine D1 receptor
D3: dopamine D3 receptor
DCCM: dynamic cross-correlation matrix
DFT: density functional theory
FEL: free energy landscape
GPCR: G protein-coupled receptor
HOMO: highest occupied molecular orbital
LD50: median lethal dose
LINCS: linear constraint solver
LSTM: long short-term memory
LUMO: lowest unoccupied molecular orbital
MD: molecular dynamics
MEP: molecular electrostatic potential
MM/GBSA: molecular mechanics generalized Born surface area
NPT: isothermal–isobaric ensemble
NVT: canonical ensemble
PCA: principal component analysis
PDB: Protein Data Bank
PME: particle mesh Ewald
P-gp: P-glycoprotein
RLS: restless legs syndrome
RMSD: root mean square deviation
RMSF: root mean square fluctuation
SASA: solvent-accessible surface area
SMILES: Simplified Molecular Input Line Entry System
TPSA: topological polar surface area.
