# Supplementary material for: 3D CNN-CVAE and LSTM-guided de novo design of rotigotine derivatives targeting dopamine D1 and D3 receptors in restless legs syndrome
Source: PLoS One. 2026 Oct 9;21(10):e0356614. doi: 10.1371/journal.pone.0356614 (PMC13652343; doi:10.1371/journal.pone.0356614)
Supplement: Supplementary file 1 [file pone.0356614.s001.docx]

**Supplementary Table S1.** AI-generated rotigotine derivatives and scaffold information. List of de novo AI-designed rotigotine derivatives with their corresponding chemical structures and core scaffolds, highlighting structural modifications based on the parent compound.

| Sr. | Compound | IUPAC Name | 2D Structure | 3D Structure |
| --- | --- | --- | --- | --- |
| Scaffold | | | | |
| 1. | Rotigotine | (S)-6-(propyl(2-(thiophen-2-yl)ethyl)amino)-5,6,7,8-tetrahydronaphthalen-1-ol |  |  |
| AI Generated Derivatives | | | | |
| 2. | AI Derivative 1 | 3-((((2,3-dihydro-1H-inden-2-yl)methyl)(isopropyl)amino)methyl)phenol |  |  |
| 3. | AI Derivative 2 | (S)-3-(((1-phenylhexan-3-yl)amino)methyl)phenol |  |  |
| 4. | AI Derivative 3 | (S)-3-(((R)-1-(naphthalen-2-yl)butyl)amino)-2-phenylpropan-1-ol |  |  |
| 5. | AI Derivative 4 | (R)-3-(3-((1-phenylbutyl)amino)propyl)phenol |  |  |
| 6. | AI Derivative 5 | (S)-3-(((2-benzyl-3-methylbutyl)amino)methyl)phenol |  |  |
| 7. | AI Derivative 6 | (R)-N-(3-fluorobenzyl)-N-(indolin-3-ylmethyl)propan-2-amine |  |  |
| 8. | AI Derivative 7 | (S)-2-fluoro-6-(((1-phenylhexan-3-yl)amino)methyl)aniline |  |  |
| 9. | AI Derivative 8 | (R)-3-(((3-hydroxy-3-phenylpropyl)(isopropyl)amino)methyl)benzonitrile |  |  |
| 10. | AI Derivative 9 | (R)-2-phenyl-4-(((R)-1-phenylbutyl)amino)butan-2-ol |  |  |
| 11. | AI Derivative 10 | N-(3-(3-fluorophenyl)propyl)-2-((isopropyl(methyl)amino)methyl)aniline |  |  |
| 12. | AI Derivative 11 | 3-((isobutyl(3-phenylpropyl)amino)methyl)phenol |  |  |
| 13. | AI Derivative 12 | (R)-N1-((R)-1-(3-fluorophenyl)butyl)-3-phenylpropane-1,3-diamine |  |  |
| 14. | AI Derivative 13 | (S)-3-(((bicyclo[4.2.0]octa-1,3,5-trien-7-ylmethyl)(isopropyl)amino)methyl)phenol |  |  |
| 15. | AI Derivative 14 | (R)-N-(3-(1H-indol-3-yl)propyl)-1-(2-fluorophenyl)butan-1-amine |  |  |
