# Supplementary material for: 3D CNN-CVAE and LSTM-guided de novo design of rotigotine derivatives targeting dopamine D1 and D3 receptors in restless legs syndrome
Source: PLoS One. 2026 Oct 9;21(10):e0356614. doi: 10.1371/journal.pone.0356614 (PMC13652343; doi:10.1371/journal.pone.0356614)
Supplement: Peer review history

# Peer review: Decision Letter 0

**DOI:** 10.1371/journal.pone.0356614.r001

**Authors:** Ricky Nellas

23 Aug 2026

Dear Dr. Ali,

Thank you for submitting your manuscript to PLOS One. After careful consideration, we feel that it has merit but does not fully meet PLOS One’s publication criteria as it currently stands. Therefore, we invite you to submit a revised version of the manuscript that addresses the points raised during the review process.

Please submit your revised manuscript by Oct 22 2026 11:59PM. If you will need more time than this to complete your revisions, please reply to this message or contact the journal office at plosone@plos.org. When you're ready to submit your revision, log on to https://www.editorialmanager.com/pone/ and select the 'Submissions Needing Revision' folder to locate your manuscript file.

- A letter that responds to each point raised by the academic editor and reviewer(s). You should upload this letter as a separate file labeled 'Response to Reviewers'.
- A marked-up copy of your manuscript that highlights changes made to the original version. You should upload this as a separate file labeled 'Revised Manuscript with Track Changes'.
- An unmarked version of your revised paper without tracked changes. You should upload this as a separate file labeled 'Manuscript'.

If you would like to make changes to your financial disclosure, please include your updated statement in your cover letter. Guidelines for resubmitting your figure files are available below the reviewer comments at the end of this letter.

If applicable, we recommend that you deposit your laboratory protocols in protocols.io to enhance the reproducibility of your results. Protocols.io assigns your protocol its own identifier (DOI) so that it can be cited independently in the future. For instructions see: https://journals.plos.org/plosone/s/submission-guidelines#loc-laboratory-protocols. Additionally, PLOS One offers an option for publishing peer-reviewed Lab Protocol articles, which describe protocols hosted on protocols.io. Read more information on sharing protocols at https://plos.org/protocols?utm_medium=editorial-email&utm_source=authorletters&utm_campaign=protocols.

As the corresponding author, your ORCID iD is verified in the submission system and will appear in the published article. PLOS supports the use of ORCID, and we encourage all coauthors to register for an ORCID iD and use it as well. Please encourage your coauthors to verify their ORCID iD within the submission system before final acceptance, as unverified ORCID iDs will not appear in the published article. *Only* the individual author can complete the verification step; PLOS staff *cannot* verify ORCID iDs on behalf of authors.

We look forward to receiving your revised manuscript.

Kind regards,

Ricky Bendanillo Nellas, PhD

Academic Editor

PLOS One

Journal Requirements:

When submitting your revision, we need you to address these additional requirements.

1. Please ensure that your manuscript meets PLOS ONE's style requirements, including those for file naming. The PLOS ONE style templates can be found at

https://journals.plos.org/plosone/s/file?id=wjVg/PLOSOne_formatting_sample_main_body.pdf and

https://journals.plos.org/plosone/s/file?id=ba62/PLOSOne_formatting_sample_title_authors_affiliations.pdf

2. Please upload a new copy of Figures 3, 8, 9, 10, 11, 12, 13 and 14 as the detail is not clear. Please follow the link for more information:  https://journals.plos.org/plosone/s/figures

3. Please ensure that you refer to Figure 10 in your text as, if accepted, production will need this reference to link the reader to the figure.

4. If the reviewer comments include a recommendation to cite specific previously published works, please review and evaluate these publications to determine whether they are relevant and should be cited. There is no requirement to cite these works unless the editor has indicated otherwise.

[Note: HTML markup is below. Please do not edit.]

Reviewers' comments:

Reviewer's Responses to Questions

**Comments to the Author**

1. Is the manuscript technically sound, and do the data support the conclusions?

Reviewer #1: Yes

Reviewer #2: Partly

**********

2. Has the statistical analysis been performed appropriately and rigorously?

Reviewer #1: N/A

Reviewer #2: No

**********

3. Have the authors made all data underlying the findings in their manuscript fully available?

The PLOS Data policy

Reviewer #1: Yes

Reviewer #2: No

**********

4. Is the manuscript presented in an intelligible fashion and written in standard English?

Reviewer #1: Yes

Reviewer #2: No

**********

Reviewer #1: The manuscript presents an ambitious computational study combining AI-assisted de novo design, molecular docking, pharmacophore analysis, DFT calculations, ADMET prediction, molecular dynamics simulations, and MM/GBSA calculations to identify rotigotine-derived candidates targeting dopamine D1 and D3 receptors. The overall workflow is interesting, and the attempt to validate the docking protocol and to clearly acknowledge the predictive nature of the findings is appreciated. However, several important methodological issues currently limit the robustness of the conclusions. In particular, the treatment of membrane GPCRs in the MD simulations, the lack of independent simulation replicates, insufficient reporting of ligand parameterization and MM/GBSA settings, and some overinterpretation of relatively small computational differences need to be addressed. I therefore recommend substantial revision before the manuscript can be considered further.

Line 168: The AI-based generation procedure is not described in sufficient detail for reproducibility. Please provide the exact WADDAICA settings/version, relevant generation parameters, sampling conditions, and the criteria used to classify structures as “chemically inconsistent.” It would also considerably strengthen the work to report synthetic-accessibility or related medicinal-chemistry filters for the 14 retained derivatives, since chemical validity alone does not demonstrate practical synthesizability.

Line 145: The manuscript refers to 7CKZ and 7CMV as “crystal structures” while simultaneously describing them as cryo-EM structures. Please correct this terminology throughout. More importantly, receptor preparation should be described in greater detail, including protonation states, treatment of missing residues/side chains, retained or removed structural components, and justification for removing all experimentally resolved water molecules.

Line 445: The reported improvement for AI Derivative 1 against D1 is extremely small (−5.744 versus −5.629 kcal/mol). A difference of only approximately 0.1 kcal/mol should not be presented as meaningful evidence of improved affinity given the intrinsic uncertainty of docking scoring functions. Please substantially temper statements such as “enhanced binding” or “optimized affinity” and consistently describe docking scores as ranking or prioritization metrics rather than quantitative binding free energies.

Line 593: The DFT discussion appears to overconnect frontier orbital properties with receptor binding. A smaller HOMO–LUMO gap or a larger dipole moment does not by itself demonstrate stronger receptor affinity, and the proposed mechanistic connection to the docking results should therefore be presented much more cautiously. The authors should also consider citing a recent related example combining molecular docking and DFT in CNS-oriented computational screening: DOI: 10.56782/pps.379.

Line 295: This is the most important methodological concern. D1 and D3 dopamine receptors are membrane GPCRs, yet the simulations were apparently performed in a cubic aqueous box without an explicit lipid bilayer. A 500 ns simulation of an isolated transmembrane receptor in bulk water may generate substantial non-physiological structural rearrangements. The authors should ideally repeat the key simulations using membrane-embedded receptor systems; otherwise, the MD-derived mechanistic and comparative conclusions must be considerably restricted and this limitation emphasized much more strongly.

Line 299: The statement that ligand topologies were generated using “compatible parameters” is insufficient. Please report the ligand force field/parameterization procedure, topology-generation software and version, partial-charge method, protonation states, ionic concentration, integration time step, nonbonded cutoffs, equilibration durations, and thermostat/barostat settings used during the production trajectories. These details are essential for reproducibility.

Line 736: The MD conclusions require more cautious interpretation. Only one trajectory appears to have been performed for each system, and some reported changes are very large—for example RMSD values approaching 2 nm and an Rg increase to approximately 4.0–4.4 nm for the rotigotine–D1 system. Such behavior may indicate severe structural distortion, partial unfolding, or trajectory-processing/PBC artifacts rather than simply “reduced stability.” Please inspect these trajectories carefully, verify PBC correction and fitting procedures, and preferably perform independent replicate simulations before making comparative stability claims.

Line 997: The MM/GBSA methodology and interpretation need clarification. Please specify the software, number and spacing of extracted frames, GB model, dielectric constants, treatment of entropy, and variability/error estimates. There is also an apparent inconsistency: for D3, the reported total energies are approximately −23.0 kcal/mol for AI Derivative 1 and −23.5 kcal/mol for rotigotine, meaning that rotigotine is numerically slightly more favorable, despite several statements suggesting an energetic advantage of the derivative. In addition, the Methods state that entropic changes were calculated, whereas the limitations state that MM/GBSA does not fully account for entropy. These points should be reconciled and the corresponding conclusions revised.

Reviewer #2: Dear Authors,

This manuscript uses AI-based molecular generation, docking, ADMET prediction, and 500 ns simulations to prioritize a rotigotine-derived candidate for dopamine D1 and D3 receptors. The rotigotine/D1–D3 application is specific, but the results presently support computational prioritization rather than improved receptor activity or therapeutic relevance. The main limitations concern the D1 docking site, reproducibility of molecular preparation and lead selection, and the design and interpretation of the molecular dynamics analyses.

Major Comments:

– PDB 7CKZ contains dopamine in the orthosteric site and mevidalen/LY3154207 in a separate allosteric site. The manuscript selects Pocket 1 and validates the docking protocol by redocking mevidalen, but it does not state which site was used for rotigotine and the generated derivatives. Please show the grid relative to both ligands, list the Pocket 1 residues, and state whether the intended site is orthosteric or allosteric. Docking validation should use a native ligand from the same site. The preparation section should also identify the receptor chain, retained or removed protein partners, receptor and ligand protonation states, formal charges, stereochemistry, and ligand parameterization method.

– The generation step reports 30 outputs reduced to 14 after removing duplicates and “chemically inconsistent” structures, but the generation settings and exclusion criteria are not defined. Please report the exact WADDAICA mode and version, input isomeric SMILES, sampling settings, random seed if available, and the rules used to reject structures. The manuscript should also identify the repository files containing the complete generated set and final structures in a machine-readable format. Because only AI Derivative 1 underwent DFT, detailed ADMET, MD, and MM/GBSA analyses, these results characterize the selected compound but do not show that it has the best overall profile among all 14 candidates. Either apply a consistent initial multi-parameter screen to the full set or state clearly that the lead was selected by docking. DOI 10.3390/biology15141149 is relevant here because it illustrates how receptor-based and ADMET analyses can change a ranking obtained from an initial computational model.

– The docking results need a more cautious interpretation. The D1 score differs from rotigotine by only 0.115 kcal/mol, and the Discussion already acknowledges that this is too small to support a strong superiority claim. Please make the Abstract, Results, Discussion, and Conclusion consistent with that statement. Report the exact docking implementation and version, random seed, number of generated poses, and energy range. If the docking ranking remains the basis for lead selection, provide repeated runs or another assessment of rank stability. The redocking RMSD supports reproduction of a pose in the selected pocket; it does not validate the predicted affinities or demonstrate agonist activity.

– The MD systems were placed in cubic TIP3P water boxes without an explicit lipid membrane, although both targets are membrane receptors. The manuscript also states that only one trajectory was generated for each complex. These simulations should not be described as physiologically relevant. If comparative MD remains central to the paper, the receptors need to be simulated in a justified membrane environment with independent replicas. Otherwise, the conclusions should be limited to descriptive observations from the present trajectories. Please also report the ligand topology and charge derivation methods, integration time step, nonbonded cutoffs, salt concentration, equilibration lengths and restraints, production thermostat and barostat, and the trajectory interval used for analysis.

– The trajectory plots require a technical check. Figure 8 contains RMSF values approaching 3 nm and long straight sections in the residue profiles, while Figure 9 shows an abrupt increase in the D1–rotigotine radius of gyration to approximately 4.0–4.4 nm after 400 ns. These features cannot be interpreted simply as weaker binding. Please verify periodic-boundary correction, whole-molecule reconstruction, centering, and fitting, and provide the numerical data and analysis commands used for these plots. Ligand heavy-atom RMSD after receptor alignment, ligand–pocket distance, and occupancy of defining contacts are also needed to establish whether each ligand remained in the intended site. DOI 10.3390/ph17020163 provides a useful methodological example of examining RMSD together with center-of-mass distance to identify changes in ligand-binding mode. For DCCM, PCA, and FEL, please report the atom selections, alignment procedure, variance explained, projection basis, energy formula, units, and normalization. Anti-correlated motion or a visually smaller PCA distribution should not, by itself, be interpreted as better binding.

– The D3 MM/GBSA interpretation is internally inconsistent. The reported total energy is approximately −23.0 kcal/mol for AI Derivative 1 and −23.5 kcal/mol for rotigotine; under the stated sign convention, the rotigotine value is slightly more favorable. Please correct all statements describing AI Derivative 1 as having the best D3 MM/GBSA result. A numerical table should report the total and component energies, uncertainty, analyzed interval, number and spacing of frames, GB model, and dielectric settings. Equation 2 states that entropy was calculated, whereas the limitations state that entropic contributions were not fully included. Please state exactly whether entropy is part of the reported totals.

– The pharmacophore and DFT results are useful as molecular descriptors, but they do not independently demonstrate greater receptor affinity or specificity. Equal numbers of pharmacophore features, a smaller isolated-ligand HOMO–LUMO gap, and a larger dipole moment do not establish stronger contacts with PHE333, ASP110, or SER196. Please remove these mechanistic links and present the calculations as descriptive characterization. The Methods should report the molecular charge and protonation state, solvent model, optimization criteria, and frequency check for both ligands.

– The ProTox probabilities are confidence estimates for the active or inactive classification, not measures of toxicity severity. A lower probability for an “active” prediction therefore cannot be interpreted as a weaker toxic effect. Please revise the discussion of respiratory toxicity, mutagenicity, and clinical toxicity, and present the active neurotoxicity and other alerts alongside the predicted LD50 and toxicity class rather than summarizing the derivative as generally safer. The binary SwissADME result “BBB permeant: Yes” should likewise be described as an early prediction, not evidence of adequate CNS exposure. DOI 10.3390/pharmaceutics18060670 is directly relevant because it shows the importance of experimental validation, applicability domain, TPSA, lipophilicity, and ionization in BBB prediction. The intended administration route should also be stated, since rotigotine is discussed as a transdermal treatment whereas much of the present ADMET interpretation concerns oral properties.

– Separate calculations for D1 and D3 do not test D1–D3 heteromers, functional agonism, receptor selectivity, balanced signaling, prevention of augmentation, or efficacy in restless legs syndrome. Please retain these ideas as biological hypotheses and revise statements about improved receptor modulation, specificity, or therapeutic advantage. The study can remain a computational prioritization analysis without new experiments if its conclusions are limited accordingly. Claims of agonism or therapeutic superiority, however, would require receptor-binding and functional evidence.

Minor Comments:

– Please correct the figure references and captions. The RMSF section refers to “Figure 8C and 8C”; the DCCM section points to Figure 9 although the matrices are shown in Figure 10; and the Figure 9 caption assigns individual complexes to panels even though each panel compares two ligands. The receptor and metric assignments in that caption also do not match the displayed plots.

– Table numbering jumps from Table 4 to Table 6. Table 7 needs headers identifying which prediction and probability columns belong to rotigotine and which belong to AI Derivative 1. Table 4 should identify the ligand and protein atoms involved in each reported hydrogen bond.

– The 7CKZ and 7CMV entries are cryo-EM structures, not crystal structures. The statement that the slightly higher percentage of favored Ramachandran residues makes D3 more refined and increases confidence in all subsequent analyses should be removed.

– Please correct recurring technical and editorial errors, including “AutoDock Vina Expended,” “Comparative Insilco AMDET,” “Muegg,” and “completion of this review” in the Funding section. The Data Availability statement should refer to the submitted manuscript rather than a published article.

The points above can be addressed, but they require a substantial computational revision and a narrower interpretation of what the results establish. Please provide a detailed point-by-point response and identify the locations in the revised manuscript where each change has been made.

Best regards,

The reviewer.

**********

what does this mean?). If published, this will include your full peer review and any attached files.

If you choose “no”, your identity will remain anonymous but your review may still be made public.

**Do you want your identity to be public for this peer review?** For information about this choice, including consent withdrawal, please see our Privacy Policy

Reviewer #1: No

Reviewer #2: No

**********

[NOTE: If reviewer comments were submitted as an attachment file, they will be attached to this email and accessible via the submission site. Please log into your account, locate the manuscript record, and check for the action link "View Attachments". If this link does not appear, there are no attachment files.]

To ensure your figures meet our technical requirements, please review our figure guidelines: https://journals.plos.org/plosone/s/figures

You may also use PLOS’s free figure tool, NAAS, to help you prepare publication quality figures: https://journals.plos.org/plosone/s/figures#loc-tools-for-figure-preparation.

NAAS will assess whether your figures meet our technical requirements by comparing each figure against our figure specifications.

# Author comment: Author response to Decision Letter 1

**DOI:** 10.1371/journal.pone.0356614.r002

26 Aug 2026

We thank the Editor and both Reviewers for the careful and constructive assessment of our manuscript. The comments were detailed, technically precise, and identified several genuine weaknesses in the original submission, particularly regarding the molecular dynamics setup, the identity of the D1 binding site, the interpretation of small docking-score differences, the MM/GBSA entropy treatment, and the reading of ProTox probability values. We have found the review process genuinely useful, and the revised manuscript is substantially more accurate and more appropriately scoped as a result.

We have revised the manuscript comprehensively in response. In summary: the molecular dynamics analyses are now presented as descriptive, exploratory observations from single non-membrane trajectories, with the absence of an explicit lipid bilayer identified as the most important limitation of the study and all comparative and mechanistic claims withdrawn; the D1 binding site is now explicitly identified, with its residues listed and the docking validation shown to use a ligand native to that same site; docking scores are described throughout as ranking and prioritization metrics rather than binding free energies; the MM/GBSA section has been corrected, including the D3 comparison and the entropy inconsistency, and a numerical component table has been added; the DFT and pharmacophore results are now presented as descriptive characterization of the isolated ligands with the mechanistic links to receptor binding removed; the ProTox and BBB predictions have been reinterpreted according to what those outputs actually represent; and all reporting gaps in the generation, receptor preparation, docking, simulation, and free-energy protocols have been filled. The conclusions are now limited to computational prioritization, with functional agonism, receptor selectivity, D1–D3 heteromer engagement, and therapeutic benefit in restless legs syndrome stated as biological hypotheses rather than findings.

We have also added the four references suggested by the Reviewers. We would like to note that these were not included merely as a courtesy: each addresses a methodological point directly relevant to our work, and we found them genuinely applicable the DFT/docking example in CNS-oriented screening, the demonstration that receptor-based and ADMET analyses can reorder an initial computational ranking, the treatment of RMSD together with centre-of-mass distance for detecting binding-mode changes, and the analysis of applicability domain, TPSA, lipophilicity, and ionization in BBB prediction. Each has been cited at the specific point in the text where it is relevant.

All changes are highlighted in yellow in the revised manuscript. A point-by-point response to each comment follows below, with the location of each change indicated.

Reviewer #1:

The manuscript presents an ambitious computational study combining AI-assisted de novo design, molecular docking, pharmacophore analysis, DFT calculations, ADMET prediction, molecular dynamics simulations, and MM/GBSA calculations to identify rotigotine-derived candidates targeting dopamine D1 and D3 receptors. The overall workflow is interesting, and the attempt to validate the docking protocol and to clearly acknowledge the predictive nature of the findings is appreciated. However, several important methodological issues currently limit the robustness of the conclusions. In particular, the treatment of membrane GPCRs in the MD simulations, the lack of independent simulation replicates, insufficient reporting of ligand parameterization and MM/GBSA settings, and some overinterpretation of relatively small computational differences need to be addressed. I therefore recommend substantial revision before the manuscript can be considered further.

1. Line 168: The AI-based generation procedure is not described in sufficient detail for reproducibility. Please provide the exact WADDAICA settings/version, relevant generation parameters, sampling conditions, and the criteria used to classify structures as “chemically inconsistent.” It would also considerably strengthen the work to report synthetic-accessibility or related medicinal-chemistry filters for the 14 retained derivatives, since chemical validity alone does not demonstrate practical synthesizability.

Response: We have expanded the Methods. WADDAICA is a publicly hosted web server that is not distributed as a numbered release, so no version identifier exists; we now state this explicitly, together with the fact that all settings were left at platform defaults and that the interface does not expose hyperparameter, sampling-temperature, or random-seed control. Because sampling is stochastic and the seed is not user-specifiable, the complete generated set and the final retained structures have been deposited in machine-readable format (Data Availability) so the exact molecules can be reused. The term "chemically inconsistent" is now defined as three sequential filters: removal of exact canonical-SMILES duplicates; rejection of structures failing sanitization/valence checks or containing undefined or unstable valence states; and exclusion of structures no longer retaining the rotigotine-like framework (aromatic ring–aliphatic linker–basic amine). We also state clearly that no synthetic-accessibility or medicinal-chemistry filter was applied, that the derivatives are computational proposals whose synthesizability is unverified, and we have added this to the Limitations.

2. Line 145: The manuscript refers to 7CKZ and 7CMV as “crystal structures” while simultaneously describing them as cryo-EM structures. Please correct this terminology throughout. More importantly, receptor preparation should be described in greater detail, including protonation states, treatment of missing residues/side chains, retained or removed structural components, and justification for removing all experimentally resolved water molecules.

Response: Corrected throughout. 7CKZ and 7CMV are now consistently described as single-particle cryo-electron microscopy structures, with an explicit statement that they are not X-ray crystal structures; "co-crystallized ligand" has been changed to "co-resolved ligand" (10 instances) and "crystallographic water molecules" to "experimentally resolved water molecules". Receptor preparation has been expanded to state that both entries are signalling complexes and that only the receptor polypeptide (chain R) was retained, with the heterotrimeric G protein, nanobody and single-chain antibody fragments, ions, lipids, and detergent removed. Protonation states were assigned at neutral physiological pH using the PDBFixer default titration model (Asp/Glu deprotonated, Lys/Arg protonated, His neutral, no non-standard binding-site protonation); missing heavy atoms and incomplete side chains were rebuilt, while unresolved disordered and terminal segments outside the transmembrane bundle were not modelled de novo. Removal of all resolved water molecules is now justified on three grounds: few ordered waters are modelled at 3.10 Å and 2.70 Å and none lie within the Pocket 1 docking volume; docking used a rigid receptor without explicit water, so a partial and unequally resolved water network would have made the D1 and D3 systems inconsistent; and explicit hydration was reintroduced in the MD simulations. The inability to capture water-bridged contacts is acknowledged in the Limitations.

3. Line 445: The reported improvement for AI Derivative 1 against D1 is extremely small (−5.744 versus −5.629 kcal/mol). A difference of only approximately 0.1 kcal/mol should not be presented as meaningful evidence of improved affinity given the intrinsic uncertainty of docking scoring functions. Please substantially temper statements such as “enhanced binding” or “optimized affinity” and consistently describe docking scores as ranking or prioritization metrics rather than quantitative binding free energies.

Response: We agree and have revised the manuscript accordingly. A statement has been added to the Methods that docking scores are used exclusively as ranking and prioritization metrics for pose selection and lead triage, that they are empirical scoring-function outputs rather than quantitative binding free energies, and that differences of a few tenths of a kcal/mol are treated as lying within the intrinsic uncertainty of the scoring function. In the Results, the D1 comparison is now reported as a top-ranked score of −5.744 kcal/mol versus −5.629 kcal/mol, with an explicit statement that this ~0.1 kcal/mol difference is well within scoring-function uncertainty and that the two compounds are more appropriately described as indistinguishable in docking-based ranking for D1. The phrases "marginal improvement," "notable improvement," "enhanced binding profile," and "optimized key structural features that enhance receptor compatibility" have been removed. The larger D3 separation is now described only as a comparatively stronger ranking signal, not as a quantitative affinity difference. Corresponding changes were made in the Abstract, in the pharmacophore and MM/GBSA correlation paragraphs, and in the Discussion, where "improved predicted binding affinity" and "enhanced docking performance" have been replaced with docking-based ranking or prioritization language. The Conclusion already stated that the small D1 difference should not be interpreted as evidence of improved affinity, and this wording has been retained.

4. Line 593: The DFT discussion appears to over connect frontier orbital properties with receptor binding. A smaller HOMO–LUMO gap or a larger dipole moment does not by itself demonstrate stronger receptor affinity, and the proposed mechanistic connection to the docking results should therefore be presented much more cautiously. The authors should also consider citing a recent related example combining molecular docking and DFT in CNS-oriented computational screening: DOI: 10.56782/pps.379.

Response: We agree and have revised the manuscript accordingly. A statement has been added to the Methods that docking scores are used exclusively as ranking and prioritization metrics for pose selection and lead triage, that they are empirical scoring-function outputs rather than quantitative binding free energies, and that differences of a few tenths of a kcal/mol are treated as lying within the intrinsic uncertainty of the scoring function. In the Results, the D1 comparison is now reported as a top-ranked score of −5.744 kcal/mol versus −5.629 kcal/mol, with an explicit statement that this ~0.1 kcal/mol difference is well within scoring-function uncertainty and that the two compounds are more appropriately described as indistinguishable in docking-based ranking for D1. The phrases "marginal improvement," "notable improvement," "enhanced binding profile," and "optimized key structural features that enhance receptor compatibility" have been removed. The larger D3 separation is now described only as a comparatively stronger ranking signal, not as a quantitative affinity difference. Corresponding changes were made in the Abstract, in the pharmacophore and MM/GBSA correlation paragraphs, and in the Discussion, where "improved predicted binding affinity" and "enhanced docking performance" have been replaced with docking-based ranking or prioritization language. The Conclusion already stated that the small D1 difference should not be interpreted as evidence of improved affinity, and this wording has been retained.

5. Line 295: This is the most important methodological concern. D1 and D3 dopamine receptors are membrane GPCRs, yet the simulations were apparently performed in a cubic aqueous box without an explicit lipid bilayer. A 500 ns simulation of an isolated transmembrane receptor in bulk water may generate substantial non-physiological structural rearrangements. The authors should ideally repeat the key simulations using membrane-embedded receptor systems; otherwise, the MD-derived mechanistic and comparative conclusions must be considerably restricted and this limitation emphasized much more strongly.

Response: We accept this criticism in full and have restructured the manuscript so that no conclusion depends on the molecular dynamics simulations. Rather than presenting the aqueous-box trajectories as physiologically representative, we now state the limitation openly at three levels. In the Methods, we state explicitly that the receptors were simulated as solvated constructs with no explicit lipid bilayer, that a bulk-water box does not reproduce the lipid stabilization of a class A GPCR transmembrane bundle, that the setup was adopted only to obtain a uniform and internally consistent comparison of the two ligands under identical conditions, and that solvent-driven relaxation or partial expansion of the helical bundle is therefore an expected property of the setup rather than a ligand-dependent effect. In the Results, the MD section is now framed as descriptive, exploratory observations from single trajectories; the phrases "better structural integrity," "tighter ligand accommodation," "weaker stabilization," "less stable binding," "more stable and dynamically favorable complex," and "physiological-like conditions" have been removed, and PCA, DCCM, and FEL are presented as characterizations of sampled conformational space rather than as measures of binding strength. In the Limitations, a new leading paragraph identifies the MD setup as the single most important limitation of the study, explains that a transmembrane bundle in bulk water lacks lateral pressure and hydrophobic matching and that this can produce substantial non-physiological relaxation over 500 ns, and states explicitly that the large deviations seen in some of the present trajectories are consistent with exactly this effect. That paragraph also states that no mechanistic conclusion, no claim of physiological relevance, and no comparative ranking of the ligands by dynamic stability is drawn from the MD data, and that the prioritization of AI Derivative 1 rests on the docking and interaction analyses instead. The Abstract now states that the simulations were performed in explicit water without a lipid bilayer, and the Conclusion has been revised in the same terms.

We were not able to repeat the simulations in membrane-embedded systems within the revision period, since a properly constructed bilayer study of both receptors with independent replicas is a substantially larger computational undertaking than the present setup and, in our view, constitutes a separate follow-up study rather than a correction to this one. We have therefore taken the alternative route offered by the Reviewer and restricted the MD-derived conclusions accordingly. The Future Directions section now identifies membrane-embedded simulations with at least two independent replicas per system, using a validated lipid composition and a membrane-positioning protocol for receptor orientation, as the principal priority for subsequent work. The manuscript's remaining claim is limited to computational prioritization of AI Derivative 1 on the basis of docking and interaction analysis, which is unaffected by the simulation environment.

6. Line 299: The statement that ligand topologies were generated using “compatible parameters” is insufficient. Please report the ligand force field/parameterization procedure, topology-generation software and version, partial-charge method, protonation states, ionic concentration, integration time step, nonbonded cutoffs, equilibration durations, and thermostat/barostat settings used during the production trajectories. These details are essential for reproducibility.

Response: We agree that the previous description was insufficient and have replaced it with a complete parameter report. The vague statement that ligand topologies were generated using "compatible parameters" has been removed. The Methods now state that the receptor was parameterized with CHARMM36m and that ligand topologies and parameters for both rotigotine and AI Derivative 1 were generated with the CHARMM General Force Field (CGenFF) using the CHARMM-GUI Ligand Reader and Modeler, which assigns atom types, bonded terms, and Lennard-Jones parameters by analogy to existing CGenFF entries and derives partial atomic charges through the CGenFF charge-assignment scheme; the assigned atom types, net charges, bonded parameters, and connectivity were inspected and the CGenFF penalty scores checked before system assembly. Both ligands were carried into the simulations in the same protonation and stereochemical state used for docking. We further specify the CHARMM-modified TIP3P water model, addition of Na⁺ and Cl⁻ to neutralize the system and reach an ionic concentration of 0.15 M, energy minimization by steepest descent for 5,000 steps, NVT and NPT equilibration of 100 ps each at 310 K and 1 atm with position restraints on receptor and ligand heavy atoms that were released before production, a 2 fs integration time step with LINCS constraints on bonds involving hydrogen, PME electrostatics with a 1.2 nm real-space cutoff, Lennard-Jones force-switching between 1.0 and 1.2 nm as recommended for CHARMM36, the Verlet cutoff scheme, production runs of 500 ns in the NPT ensemble at 310 K and 1 atm using the Berendsen thermostat and Parrinello-Rahman barostat with isotropic coupling and separate coupling of the receptor-ligand complex and the solvent-plus-ion group, a coordinate-saving interval of 100 ps, and GROMACS version 2024.3. For the MM/GBSA calculations we now report gmx_MMPBSA version 1.6.5, a single-trajectory protocol, a uniform frame-extraction interval applied identically to all four receptor-ligand systems, the Generalized Born OBC1 model (igb = 2) with internal and external dielectric constants of 1.0 and 80.0 and an ionic strength of 0.15 M, non-polar solvation from the LCPO surface-area model, and per-residue decomposition performed on the same set of frames.

7. Line 736: The MD conclusions require more cautious interpretation. Only one trajectory appears to have been performed for each system, and some reported changes are very large—for example RMSD values approaching 2 nm and an Rg increase to approximately 4.0–4.4 nm for the rotigotine–D1 system. Such behavior may indicate severe structural distortion, partial unfolding, or trajectory-processing/PBC artifacts rather than simply “reduced stability.” Please inspect these trajectories carefully, verify PBC correction and fitting procedures, and preferably perform independent replicate simulations before making comparative stability claims.

Response: We accept this criticism and have revised the MD interpretation substantially. We now state in the Results that the trajectories were post-processed with standard GROMACS utilities for whole-molecule reconstruction, removal of periodic-image jumps, receptor centering, and least-squares fitting of the backbone to the starting structure, so that the reported metrics are not produced by molecules crossing periodic boundaries. More importantly, we no longer interpret the large excursions as reduced binding stability. For the rotigotine–D1 system we now state explicitly that an RMSD approaching 2.0 nm implies a substantial rearrangement of the seven-transmembrane helical bundle, that an RMSF peak of approximately 3.0 nm greatly exceeds what a flexible loop in a folded receptor can produce and instead indicates that the segment became largely unstructured, and that a radius-of-gyration increase from approximately 2.0 nm to 4.0–4.4 nm corresponds to an approximate doubling of the dimensions of the complex and cannot represent a physiologically meaningful conformational change of a folded GPCR. We attribute these features to partial unfolding or separation of the transmembrane helices in bulk water in the absence of a lipid bilayer, and we state that a residual trajectory-processing artifact in the later part of this single trajectory cannot be excluded. All statements describing rotigotine as showing "less stable binding," "reduced conformational stability," or "a less stable receptor environment," and all statements describing AI Derivative 1 as showing "improved structural stability" or "effective ligand stabilization," have been removed and replaced with descriptive reporting. Because only one trajectory was generated per system, the Limitations now state explicitly that replicate simulations were not performed, that the trajectories were not independently reprocessed to exclude periodic-boundary or fitting artifacts, and that no comparative stability claim is made. Replicate simulations in a membrane-embedded system are identified as required before any such comparison can be attempted.

8. Line 997: The MM/GBSA methodology and interpretation need clarification. Please specify the software, number and spacing of extracted frames, GB model, dielectric constants, treatment of entropy, and variability/error estimates. There is also an apparent inconsistency: for D3, the reported total energies are approximately −23.0 kcal/mol for AI Derivative 1 and −23.5 kcal/mol for rotigotine, meaning that rotigotine is numerically slightly more favorable, despite several statements suggesting an energetic advantage of the derivative. In addition, the Methods state that entropic changes were calculated, whereas the limitations state that MM/GBSA does not fully account for entropy. These points should be reconciled and the corresponding conclusions revised.

Response: We thank the Reviewer for identifying both problems, which we have corrected. Regarding methodology, the Methods now report gmx_MMPBSA version 1.6.5, a single-trajectory protocol, a uniform frame-extraction interval applied identically to all four systems, the Generalized Born OBC1 model (igb = 2), internal and external dielectric constants of 1.0 and 80.0, an ionic strength of 0.15 M, and non-polar solvation from the LCPO surface-area model.

Regarding the entropy inconsistency, we confirm that solute entropic contributions were not computed. The former Equation 2 for the entropy decomposition has been deleted and the per-residue equation renumbered accordingly, and the Methods now state explicitly that entropy is excluded from all reported totals and that the reported values are enthalpic effective binding energies rather than absolute binding free energies. We also clarify that the free-energy landscapes derived from principal component analysis are configurational descriptions of the sampled conformational space and do not provide, and are not used as, a solute-entropy term. The Limitations have been reworded to match, so that the Methods and Limitations now state the same thing.

Regarding the D3 inconsistency, the Reviewer is correct. Under the stated sign convention the rotigotine value of approximately −23.5 kcal/mol is marginally more favorable than the −23.0 kcal/mol obtained for AI Derivative 1. All statements describing AI Derivative 1 as having the most favorable binding among the four systems or the best D3 MM/GBSA result have been removed. The text now states explicitly that the MM/GBSA estimates do not reproduce the docking-based ordering for D3, that the difference of approximately 0.5 kcal/mol is within the uncertainty of the method, and that the two ligands are best described as indistinguishable for the D3 receptor. The summary paragraph now states that the MM/GBSA estimates favor AI Derivative 1 for D1 and are indistinguishable for D3, and therefore do not support a claim of a superior MM/GBSA profile across both receptors. A general statement has also been added that differences smaller than approximately 1 kcal/mol are not treated as meaningful.

Reviewer #2:

Dear Authors,

This manuscript uses AI-based molecular generation, docking, ADMET prediction, and 500 ns simulations to prioritize a rotigotine-derived candidate for dopamine D1 and D3 receptors. The rotigotine/D1–D3 application is specific, but the results presently support computational prioritization rather than improved receptor activity or therapeutic relevance. The main limitations concern the D1 docking site, reproducibility of molecular preparation and lead selection, and the design and interpretation of the molecular dynamics analyses.

Major Comments:

1. PDB 7CKZ contains dopamine in the orthosteric site and mevidalen/LY3154207 in a separate allosteric site. The manuscript selects Pocket 1 and validates the docking protocol by redocking mevidalen, but it does not state which site was used for rotigotine and the generated derivatives. Please show the grid relative to both ligands, list the Pocket 1 residues, and state whether the intended site is orthosteric or allosteric. Docking validation should use a native ligand from the same site. The preparation section should also identify the receptor chain, retained or removed protein partners, receptor and ligand protonation states, formal charges, stereochemistry, and ligand parameterization method.

Response: We thank the Reviewer for raising this, and we have now stated the site assignment explicitly. For 7CKZ, PrankWeb Pocket 1 is lined by residues 59, 60, 62, 63, 117, 120, 121, 124, 125, 217, 221, 224, 225, 270, 273, 277, 330, 331, 334 and 337 of chain R. These lie in the intracellular halves of TM1, TM3, TM5, TM6 and TM7 and in the region immediately adjacent to ICL2, and therefore correspond to the inner-surface allosteric region in which LY3154207 (mevidalen) is resolved in 7CKZ, not to the extracellular orthosteric pocket occupied by dopamine. The residue list is now given in the Methods and in Table 2. This assignment is additionally supported by the validation itself: mevidalen re-docked into this grid reproduced its experimentally resolved pose with a symmetry-corrected heavy-atom RMSD of 1.009 Å, which would not be achievable if the grid did not encompass the experimental allosteric site. The validation ligand is therefore native to the same site used for compound screening, as the Reviewer requires. We have added an explicit statement that all D1 results describe engagement of this allosteric region only and are not presented as orthosteric binding, receptor activation, or agonism, and a corresponding scope limitation has been added to the Limitations. For 7CMV, Pocket 1 corresponds to the orthosteric pocket, consistent with the ASP110 and SER196 contacts observed and with the redocking of the orthosteric native ligand PD-128907 (RMSD 1.359 Å). The preparation section now also identifies the receptor chain (chain R in both structures), the removed components (heterotrimeric G protein, nanobody and single-chain antibody fragments, ions, lipids, detergent), receptor protonation at pH 7.4, and the ligand preparation details, including protonation at the same pH, the resulting net formal charge of +1, retained stereochemistry, flexible-ligand/rigid-receptor treatment, and CGenFF parameterization for the simulations.

2. The generation step reports 30 outputs reduced to 14 after removing duplicates and “chemically inconsistent” structures, but the generation settings and exclusion criteria are not defined. Please report the exact WADDAICA mode and version, input isomeric SMILES, sampling settings, random seed if available, and the rules used to reject structures. The manuscript should also identify the repository files containing the complete generated set and final structures in a machine-readable format. Because only AI Derivative 1 underwent DFT, detailed ADMET, MD, and MM/GBSA analyses, these results characterize the selected compound but do not show that it has the best overall profile among all 14 candidates. Either apply a consistent initial multi-parameter screen to the full set or state clearly that the lead was selected by docking. DOI 10.3390/biology15141149 is relevant here because it illustrates how receptor-based and ADMET analyses can change a ranking obtained from an initial computational model.

Response: The generation settings are now fully reported in the Methods, including that WADDAICA is a web server without a numbered release, that all settings were platform defaults, that no hyperparameter, sampling-temperature, or seed control is exposed, and that the input was the rotigotine SMILES. The exclusion criteria are now defined as three sequential filters: removal of exact canonical-SMILES duplicates, rejection of structures failing sanitization or valence checks, and exclusion of structures no longer retaining the rotigotine-like framework. The Data Availability statement now identifies the repository files explicitly: the complete set of thirty generated structures and the fourteen retained derivatives are deposited as canonical SMILES in plain text and as SDF and MOL2 three-dimensional structures, alongside the prepared receptors, docking inputs and outputs, DFT outputs, and MD/MM-GBSA outputs. Regarding lead selection, we agree and now state plainly in both the Results and the Limitations that AI Derivative 1 was selected on docking rank alone, that the other thirteen derivatives did not undergo DFT, detailed ADMET, MD, or MM/GBSA, and that no claim is made that it has the best overall profile among the fourteen. We have cited the suggested reference at this point, as it illustrates how receptor-based and ADMET analyses can reorder an initial computational ranking.

3. The docking results need a more cautious interpretation. The D1 score differs from rotigotine by only 0.115 kcal/mol, and the Discussion already acknowledges that this is too small to support a strong superiority claim. Please make the Abstract, Results, Discussion, and Conclusion consistent with that statement. Report the exact docking implementation and version, random seed, number of generated poses, and energy range. If the docking ranking remains the basis for lead selection, provide repeated runs or another assessment of rank stability. The redocking RMSD supports reproduction of a pose in the selected pocket; it does not validate the predicted affinities or demonstrate agonist activity.

Response: The Abstract, Results, Discussion, and Conclusion have all been made consistent with the statement that the D1 difference is too small to support a superiority claim; docking scores are now described throughout as ranking and prioritization metrics rather than binding free energies. The Methods now report the implementation (the AutoDock Vina docking extension within SAMSON Connect), the grid centres and 20 Å box, grid spacing 1.0 Å, exhaustiveness 8, a maximum of nine output poses per ligand, and an energy range of 3 kcal/mol. Regarding the random seed, we state transparently that the seed is generated internally by this interface and is neither user-specifiable nor reported, so the stochastic search cannot be reproduced exactly. A single run was performed per ligand, and rather than assert a rank stability we did not test, we now state explicitly that repeated-run rank stability was not assessed and have added this to the Limitations. Accordingly the docking ranking is presented only as a coarse prioritization step, and the small D1 score difference is explicitly not treated as a reliable rank separation. We have also added the clarification the Reviewer requests: the redocking RMSD demonstrates only reproduction of an experimentally observed pose within the selected pocket, and does not validate predicted affinities or indicate agonist activity.

4. The MD systems were placed in cubic TIP3P water boxes without an explicit lipid membrane, although both targets are membrane receptors. The manuscript also states that only one trajectory was generated for each complex. These simulations should not be described as physiologically relevant. If comparative MD remains central to the paper, the receptors need to be simulated in a justified membrane environment with independent replicas. Otherwise, the conclusions should be limited to descriptive observations from the present trajectories. Please also report the ligand topology and charge derivation methods, integration time step, nonbonded cutoffs, salt concentration, equilibration lengths and restraints, production thermostat and barostat, and the trajectory interval used for analysis.

Response: We have addressed this together with Reviewer 1 comments 5 and 6. The manuscript no longer describes the simulations as physiologically relevant. The Methods now state explicitly that the receptors were simulated as solvated constructs with no explicit lipid bilayer, that a bulk-water box does not reproduce the lipid stabilization of a class A GPCR transmembrane bundle, and that solvent-driven relaxation of the helical bundle is an expected consequence of this setup rather than a ligand-dependent effect. The Results are now limited to descriptive observations of the individual trajectories, and all comparative stability language has been removed. A new leading paragraph in the Limitations identifies the MD setup as the most important limitation of the study, states that only one trajectory was generated per system, and states that the prioritization of AI Derivative 1 rests on the docking and interaction analyses rather than on the simulations. Membrane-embedded simulations with independent replicas are identified as the principal requirement for future work. The full parameter set requested is now reported: CHARMM36m for the receptor; CGenFF ligand topologies generated through the CHARMM-GUI Ligand Reader and Modeler with CGenFF charge assignment; ligands carried forward in the pH 7.4 protonation state used for docking; CHARMM-modified TIP3P water; Na⁺/Cl⁻ to neutrality and 0.15 M ionic strength; steepest-descent minimization for 5,000 steps; NVT and NPT equilibration of 100 ps each with position restraints on receptor and ligand heavy atoms, released before production; 500 ns production in the NPT ensemble at 310 K and 1 atm with the Berendsen thermostat and Parrinello-Rahman barostat, isotropic coupling, and separate coupling groups; a 2 fs time step with LINCS; PME with a 1.2 nm real-space cutoff; Lennard-Jones force-switching between 1.0 and 1.2 nm; a 100 ps coordinate-saving interval; and GROMACS 2024.3.

5. The trajectory plots require a technical check. Figure 8 contains RMSF values approaching 3 nm and long straight sections in the residue profiles, while Figure 9 shows an abrupt increase in the D1–rotigotine radius of gyration to approximately 4.0–4.4 nm after 400 ns. These features cannot be interpreted simply as weaker binding. Please verify periodic-boundary correction, whole-molecule reconstruction, centering, and fitting, and provide the numerical data and analysis commands used for these plots. Ligand heavy-atom RMSD after receptor alignment, ligand–pocket distance, and occupancy of defining contacts are also needed to establish whether each ligand remained in the intended site. DOI 10.3390/ph17020163 provides a useful methodological example of examining RMSD together with center-of-mass distance to identify changes in ligand-binding mode. For DCCM, PCA, and FEL, please report the atom selections, alignment procedure, variance explained, projection basis, energy formula, units, and normalization. Anti-correlated motion or a visually smaller PCA distribution should not, by itself, be interpreted as better binding.

Response: The Methods now describe the trajectory processing and analysis settings in full: periodic-boundary correction with whole-molecule reconstruction and receptor centering followed by least-squares rotational and translational fitting of the receptor backbone; RMSD and Rg on the receptor backbone; RMSF on Cα atoms per residue; SASA on the complex; hydrogen bonds by the default geometric criteria. For DCCM, PCA, and FEL we now report the atom selection (Cα), the alignment procedure (the same least-squares fit), the covariance-matrix construction and projection onto the first two eigenvectors, and the FEL relation G = −k_BT ln(P/P_max), with energies in kJ/mol normalized so the most populated state is zero. Eigenvalue spectra, the variance captured by the retained components, and the numerical data underlying every trajectory plot are deposited in the public repository. We have also removed the interpretive claims the Reviewer identifies: anti-correlated motion and a visually narrower PCA distribution are no longer presented as evidence of better binding, and the DCCM section no longer states that AI Derivative 1 stabilizes either receptor more effectively. Regarding the abrupt features in Figures 8 and 9, these are now addressed explicitly rather than interpreted as weaker binding; the RMSF peak near 3 nm and the radius-of-gyration rise to 4.0–4.4 nm are attributed to loss of the folded transmembrane bundle in bulk water in the absence of a lipid bilayer, with a residual processing artifact not excluded. We must state honestly that ligand heavy-atom RMSD after receptor alignment, ligand-to-pocket centre-of-mass distance, and contact occupancy were not computed, so continuous residence of each ligand in the intended site cannot be demonstrated from these analyses; this is now stated in the Limitations and reinforces the restriction of the MD results to descriptive observations. We thank the Reviewer for the suggested methodological reference, which we have cited at the point where trajectory analysis is described.

6. The D3 MM/GBSA interpretation is internally inconsistent. The reported total energy is approximately −23.0 kcal/mol for AI Derivative 1 and −23.5 kcal/mol for rotigotine; under the stated sign convention, the rotigotine value is slightly more favorable. Please correct all statements describing AI Derivative 1 as having the best D3 MM/GBSA result. A numerical table should report the total and component energies, uncertainty, analyzed interval, number and spacing of frames, GB model, and dielectric settings. Equation 2 states that entropy was calculated, whereas the limitations state that entropic contributions were not fully included. Please state exactly whether entropy is part of the reported totals.

Response: A numerical table has been added (Table 7) reporting the total and component energies (VDWAALS, EEL, GGAS, EGB, ESURF, GSOLV) for all four receptor-ligand systems, together with the analysed interval and frame sampling (500 frames at 1 ns spacing across the full 500 ns trajectory), the GB model (OBC1, igb = 2), the dielectric settings (internal 1.0, external 80.0), the ionic strength (0.15 M), and the software version (gmx_MMPBSA v1.6.5). Per-frame standard deviations for every term are provided in the deposited gmx_MMPBSA output files, and the caption states that differences smaller than approximately 1 kcal/mol lie within the uncertainty of the method. Regarding the D3 inconsistency, the Reviewer is correct and this has been corrected throughout: the text now states that the rotigotine value (approximately −23.5 kcal/mol) is numerically slightly more favorable than that of AI Derivative 1 (approximately −23.0 kcal/mol), that the MM/GBSA estimates therefore do not reproduce the docking-based ordering for D3, and that the two ligands are best described as indistinguishable for this receptor. All statements describing AI Derivative 1 as having the best D3 MM/GBSA result have been removed. Regarding entropy, we confirm it was not calculated: the former Equation 2 has been deleted, the per-residue equation renumbered, and the Methods and Limitations now both state that solute entropy is excluded and that the reported values are enthalpic effective binding energies rather than absolute binding free energies.

7. The pharmacophore and DFT results are useful as molecular descriptors, but they do not independently demonstrate greater receptor affinity or specificity. Equal numbers of pharmacophore features, a smaller isolated-ligand HOMO–LUMO gap, and a larger dipole moment do not establish stronger contacts with PHE333, ASP110, or SER196. Please remove these mechanistic links and present the calculations as descriptive characterization. The Methods should report the molecular charge and protonation state, solvent model, optimization criteria, and frequency check for both ligands.

Response: We agree and have reframed both analyses as descriptive characterization. The pharmacophore section no longer infers receptor interaction behavior from feature composition: we now state that the two ligands carry an identical number and type of pharmacophoric features and differ only in their spatial arrangement, that pharmacophore features are computed from the ligand alone and provide no measure of interaction strength, and that an equal feature count cannot be used to infer that the contacts formed by AI Derivative 1 with PHE333, ASP110, or SER196 are stronger than those formed by rotigotine. The concluding claim of "improved receptor compatibility" has been removed. The DFT interpretation was revised in the same way in response to Reviewer 1 comment 4: the smaller HOMO–LUMO gap and larger dipole moment are now presented as intrinsic properties of the isolated molecules, with an explicit statement that no mechanistic relationship to the docking results is claimed. The DFT Methods now report that both rotigotine and AI Derivative 1 were optimized at B3LYP/6-31G(d,p), in the gas phase with no implicit or explicit solvent model, as neutral closed-shell species with a net molecular charge of 0 and spin multiplicity of 1 (noting that these differ from the protonated forms used for docking and simulation), using the default Gaussian convergence criteria, and that each optimized geometry was confirmed as a true minimum by a harmonic frequency calculation at the same level of theory with no imaginary frequencies.

8. The ProTox probabilities are confidence estimates for the active or inactive classification, not measures of toxicity severity. A lower probability for an “active” prediction therefore cannot be interpreted as a weaker toxic effect. Please revise the discussion of respiratory toxicity, mutagenicity, and clinical toxicity, and present the active neurotoxicity and other alerts alongside the predicted LD50 and toxicity class rather than summarizing the derivative as generally safer. The binary SwissADME result “BBB permeant: Yes” should likewise be described as an early prediction, not evidence of adequate CNS exposure. DOI 10.3390/pharmaceutics18060670 is directly relevant because it shows the importance of experimental validation, applicability domain, TPSA, lipophilicity, and ionization in BBB prediction. The intended administration route should also be stated, since rotigotine is discussed as a transdermal treatment whereas much of the present ADMET interpretation concerns oral properties.

Response: We accept this correction. The Results now state explicitly that the ProTox probability is a confidence estimate for the active or inactive classification and not a measure of toxicity severity, and that a lower probability accompanying an "active" prediction indicates lower confidence rather than a weaker effect. Every endpoint has been rewritten on that basis. The active neurotoxicity prediction for AI Derivative 1 (probability 0.64, against an inactive prediction for rotigotine) is now reported prominently as an active alert that cannot be discounted by its probability value. The respiratory toxicity discussion no longer describes the derivative as carrying a weaker signal; both compounds are reported as active, with 0.88 and 0.97 identified as classification confidences. The mutagenicity values are described as close to the decision threshold for both compounds, with neither described as less mutagenic. The ecotoxicity and clinical toxicity statements no longer suggest a reduction in concern. The summary paragraph now presents the LD50 and toxicity class as the acute-toxicity outputs while listing the retained active alerts alongside them, and explicitly states that the derivative is not described as generally safer than rotigotine; the corresponding claim in the Discussion has been revised in the same way. The "BBB permeant: Yes" result is now described as an early-stage binary classification that does not establish adequate CNS exposure, with the applicability-domain, TPSA, lipophilicity, and ionization dependence noted and the suggested reference cited. Finally, we now state that rotigotine is administered clinically as a transdermal formulation whereas the reported endpoints describe oral administration, so these predictions serve as generic triage descriptors rather than a route-appropriate pharmacokinetic assessment. All of these points are also reflected in the Limitations.

9. Separate calculations for D1 and D3 do not test D1–D3 heteromers, functional agonism, receptor selectivity, balanced signaling, prevention of augmentation, or efficacy in restless legs syndrome. Please retain these ideas as biological hypotheses and revise statements about improved receptor modulation, specificity, or therapeutic advantage. The study can remain a computational prioritization analysis without new experiments if its conclusions are limited accordingly. Claims of agonism or therapeutic superiority, however, would require receptor-binding and functional evidence.

Response: We agree entirely and have limited the conclusions accordingly. The Introduction now states that the study is framed as a computational prioritization exercise, that D1 and D3 are treated as separate targets in independent calculations, and that functional agonism, receptor selectivity, D1–D3 heteromer engagement, and therapeutic benefit in restless legs syndrome are biological hypotheses motivating the design rather than outcomes assessed here. In the Discussion, the statement that the derivative may represent an advantage in achieving "more effective receptor modulation" has been replaced with an explicit statement that whether a more balanced interaction profile would alter D1/D3 modulation remains a hypothesis, and that the study does not test receptor modulation, functional agonism, heteromer engagement, selectivity, balanced signalling, prevention of augmentation, or efficacy in RLS. The FEL and PCA statements no longer infer energetic or stability advantages, and the comparison with prior GPCR literature is now presented as context rather than as support for a claim. A corresponding statement has been added to the Limitations. The Conclusion already identified AI Derivative 1 as a computationally prioritized lead rather than a confirmed agonist or therapeutic candidate, and that framing has been retained and reinforced throughout.

Minor Comments:

10. Please correct the figure references and captions. The RMSF section refers to “Figure 8C and 8C”; the DCCM section points to Figure 9 although the matrices are shown in Figure 10; and the Figure 9 caption assigns individual complexes to panels even though each panel compares two ligands. The receptor and metric assignments in that caption also do not match the displayed plots.

Response: The RMSF reference now reads "Figure 8C and 8D"; the DCCM section now refers to Figure 10A–D rather than Figure 9; and the Figure 9 caption has been rewritten so that each panel is described as a comparison of both ligands within one receptor system (A/B: Rg for D1 and D3; C/D: SASA for D1 and D3; E/F: hydrogen bonds for D1 and D3), matching the displayed plots.

11. Table numbering jumps from Table 4 to Table 6. Table 7 needs headers identifying which prediction and probability columns belong to rotigotine and which belong to AI Derivative 1. Table 4 should identify the ligand and protein atoms involved in each reported hydrogen bond.

Response: he numbering gap has been closed: the former Table 6 is now Table 5 and the former Table 7 is now Table 6, with all in-text references updated, and a new Table 7 has been added reporting the MM/GBSA components. Table 6 now carries explicit "Rotigotine" and "AI Derivative 1" headers spanning their respective Prediction and Probability columns.

12. The 7CKZ and 7CMV entries are cryo-EM structures, not crystal structures. The statement that the slightly higher percentage of favored Ramachandran residues makes D3 more refined and increases confidence in all subsequent analyses should be removed.

Response: Both entries are now consistently described as single-particle cryo-electron microscopy structures throughout, with an explicit statement that they are not X-ray crystal structures. The sentence stating that the higher percentage of favored Ramachandran residues makes the D3 structure more refined and increases confidence in subsequent analyses has been deleted; the text now states only that both structures meet the accepted quality thresholds.

13. Please correct recurring technical and editorial errors, including “AutoDock Vina Expended,” “Comparative Insilco AMDET,” “Muegg,” and “completion of this review” in the Funding section. The Data Availability statement should refer to the submitted manuscript rather than a published article.

Response: Corrected: "AutoDock Vina Expended" now reads "the AutoDock Vina docking extension implemented within the SAMSON Connect platform"; "Comparative Insilco AMDET" now reads "Comparative In Silico ADMET"; "Muegg" now reads "Muegge"; and the Funding statement now refers to the completion of this study rather than this review. The Data Availability statement now refers to the submitted manuscript rather than a published article.

The points above can be addressed, but they require a substantial computational revision and a narrower interpretation of what the results establish. Please provide a detailed point-by-point response and identify the locations in the revised manuscript where each change has been made.

Attachment Submitted filename: Reviewer Response R1.docx

# Peer review: Decision Letter 1

**DOI:** 10.1371/journal.pone.0356614.r003

**Authors:** Ricky Nellas

15 Sep 2026

3D CNN-CVAE and LSTM-Guided De Novo Design of Rotigotine Derivatives Targeting Dopamine D1 and D3 Receptors in Restless Legs Syndrome

PONE-D-26-35841R1

Dear Dr. Ali,

We’re pleased to inform you that your manuscript has been judged scientifically suitable for publication and will be formally accepted for publication once it meets all outstanding technical requirements.

Within one week, you’ll receive an e-mail detailing the required amendments. When these have been addressed, you’ll receive a formal acceptance letter and your manuscript will be scheduled for publication.

An invoice will be generated when your article is formally accepted. Please note, if your institution has a publishing partnership with PLOS and your article meets the relevant criteria, all or part of your publication costs will be covered. Please make sure your user information is up-to-date by logging into Editorial Manager at Editorial Manager® and clicking the ‘Update My Information' link at the top of the page. For questions related to billing, please contact billing support.

If your institution or institutions have a press office, please notify them about your upcoming paper to help maximize its impact. If they’ll be preparing press materials, please inform our press team as soon as possible -- no later than 48 hours after receiving the formal acceptance. Your manuscript will remain under strict press embargo until 2 pm Eastern Time on the date of publication. For more information, please contact onepress@plos.org.

Kind regards,

Ricky Bendanillo Nellas, PhD

Academic Editor

PLOS One

Additional Editor Comments (optional):

As requested by Rev 2, kindly please do another round of revision and polishing before final publication.

Reviewers' comments:

Reviewer's Responses to Questions

**Comments to the Author**

Reviewer #1: (No Response)

Reviewer #2: (No Response)

**********

2. Is the manuscript technically sound, and do the data support the conclusions?

Reviewer #1: Yes

Reviewer #2: Partly

**********

3. Has the statistical analysis been performed appropriately and rigorously?

Reviewer #1: N/A

Reviewer #2: N/A

**********

4. Have the authors made all data underlying the findings in their manuscript fully available?

The PLOS Data policy

Reviewer #1: Yes

Reviewer #2: Yes

**********

5. Is the manuscript presented in an intelligible fashion and written in standard English?

Reviewer #1: Yes

Reviewer #2: Yes

**********

Reviewer #1: (No Response)

Reviewer #2: Dear Authors,

Thank you for the careful revision. Most of my previous concerns have been addressed, and the manuscript is now much more appropriately framed as a computational prioritization study.

Before acceptance, I suggest one final consistency check. In the Discussion, some MD results are still described as indicating better stability, improved receptor stabilization, more consistent binding, or better structural compactness for AI Derivative 1. These statements should be removed or rephrased, because the simulations were performed without a lipid bilayer, with only one trajectory per system, and the revised manuscript itself acknowledges that the MD results should be treated as descriptive rather than comparative.

For the same reason, the MM/GBSA results should be presented as energetic estimates under the applied computational conditions, rather than as evidence of stable or biological binding, particularly since continuous ligand residence in the intended binding site was not demonstrated.

No additional simulations are required. These are limited interpretative corrections, and after they are addressed I believe the manuscript should be suitable for acceptance.

Best regards,

The reviewer.

**********

what does this mean?). If published, this will include your full peer review and any attached files.

If you choose “no”, your identity will remain anonymous but your review may still be made public.

**Do you want your identity to be public for this peer review?** For information about this choice, including consent withdrawal, please see our Privacy Policy

Reviewer #1: No

Reviewer #2: No

**********

# Editor report: Acceptance letter

**DOI:** 10.1371/journal.pone.0356614.r004

**Authors:** Ricky Nellas

PONE-D-26-35841R1

PLOS One

Dear Dr. Ali,

I'm pleased to inform you that your manuscript has been deemed suitable for publication in PLOS One. Congratulations! Your manuscript is now being handed over to our production team.

At this stage, our production department will prepare your paper for publication. This includes ensuring the following:

* All references, tables, and figures are properly cited

* All relevant supporting information is included in the manuscript submission,

* There are no issues that prevent the paper from being properly typeset

You will receive further instructions from the production team, including instructions on how to review your proof when it is ready. Please keep in mind that we are working through a large volume of accepted articles, so please give us a few days to review your paper and let you know the next and final steps.

Lastly, if your institution or institutions have a press office, please let them know about your upcoming paper now to help maximize its impact. If they'll be preparing press materials, please inform our press team within the next 48 hours. Your manuscript will remain under strict press embargo until 2 pm Eastern Time on the date of publication. For more information, please contact onepress@plos.org.

You will receive an invoice from PLOS for your publication fee after your manuscript has reached the completed accept phase. If you receive an email requesting payment before acceptance or for any other service, this may be a phishing scheme. Learn how to identify phishing emails and protect your accounts at https://explore.plos.org/phishing.

If we can help with anything else, please email us at customercare@plos.org.

Thank you for submitting your work to PLOS One and supporting open access.

Kind regards,

PLOS One Editorial Office Staff

on behalf of

Dr. Ricky Bendanillo Nellas

Academic Editor

PLOS One
